# Characterization of a novel RXR receptor in the salmon louse (*Lepeophtheirus salmonis*, Copepoda) regulating growth and female reproduction

**DOI:** 10.1186/s12864-015-1277-y

**Published:** 2015-02-14

**Authors:** Christiane Eichner, Sussie Dalvin, Rasmus Skern-Mauritzen, Ketil Malde, Heidi Kongshaug, Frank Nilsen

**Affiliations:** Department of Biology, Sea Lice Research Centre, University of Bergen, Bergen, Norway; Institute of Marine Research, Bergen, Norway

**Keywords:** Ultraspiracle, Retinoid X receptor, Sea louse, Copepod, Atlantic salmon, RNAi, Microarray

## Abstract

**Background:**

Nuclear receptors have crucial roles in all metazoan animals as regulators of gene transcription. A wide range of studies have elucidated molecular and biological significance of nuclear receptors but there are still a large number of animals where the knowledge is very limited. In the present study we have identified an RXR type of nuclear receptor in the salmon louse (*Lepeophtheirus salmonis*) (i.e. LsRXR). RXR is one of the two partners of the Ecdysteroid receptor in arthropods, the receptor for the main molting hormone 20-hydroxyecdysone (E20) with a wide array of effects in arthropods.

**Results:**

Five different LsRXR transcripts were identified by RACE showing large differences in domain structure. The largest isoforms contained complete DNA binding domain (DBD) and ligand binding domain (LBD), whereas some variants had incomplete or no DBD. LsRXR is transcribed in several tissues in the salmon louse including ovary, subcuticular tissue, intestine and glands. By using Q-PCR it is evident that the LsRXR mRNA levels vary throughout the *L. salmonis* life cycle. We also show that the truncated LsRXR transcript comprise about 50% in all examined samples. We used RNAi to knock-down the transcription in adult reproducing female lice. This resulted in close to zero viable offspring. We also assessed the LsRXR RNAi effects using a *L. salmonis* microarray and saw significant effects on transcription in the female lice. Transcription of the major yolk proteins was strongly reduced by knock-down of LsRXR. Genes involved in lipid metabolism and transport were also down regulated. Furthermore, different types of growth processes were up regulated and many cuticle proteins were present in this group.

**Conclusions:**

The present study demonstrates the significance of LsRXR in adult female *L. salmonis* and discusses the functional aspects in relation to other arthropods. LsRXR has a unique structure that should be elucidated in the future.

**Electronic supplementary material:**

The online version of this article (doi:10.1186/s12864-015-1277-y) contains supplementary material, which is available to authorized users.

## Background

Nuclear receptors (NR) are ligand dependent transcription factors that regulate a range of different biological processes and they have been identified in all main groups of metazoan animals. The members of the nuclear receptor super family are characterized by a highly conserved DNA-binding domain (DBD) and a less conserved C-terminal ligand binding domain (LBD). After ligand binding the NR DBD domain can interact with DNA and activate transcription.

Retinoid X Receptors (RXRs), commonly known as ultraspiracle (USP) in insects [1], belong to the nuclear receptor superfamily and targets multiple signaling pathways. RXRs are involved in controlling ontogenetic development as well as processes in adult individuals including reproduction and metabolic pathways. RXRs can activate transcription either as homodimers or as obligate heterodimeric partners with other nuclear receptors. Among nuclear receptors the RXRs ability to form both homodimers and heterodimers is unique. A critical element in regulating gene expression is the conformational change in RXRs induced by ligand binding. This change results in increased ligand affinity and transcription machinery assembly. Whereas the ligand for RXR is known to be 9-cis retinoic acid [2] the ligands of USPs remain unknown.

In insects, USP forms a heterodimer with the ecdysteroid receptor (EcR) [3]. Ecdysteroids are a family of arthropod specific steroid hormones with 20-hydroxyecdysone (20E) as the main active form. The EcR and USP heterodimer binds to the hormone response elements present in the promoters of ecdysone response genes and regulate their transcription [4,5]. The EcR-USP heterodimer complex plays an important role in development, growth and molting and reproduction in arthropods. In *Drosophila,* a reduction of maternal and zygotic USP levels results in embryonic lethality [6-8]. By phenotypic analysis of USP mutants in *Drosophila*, Oro, et al. [6] showed that USP is needed in many developmental stages in various tissues, somatic as well as germ line. In addition, maternally derived USP is required for embryonic development and USP is necessary during oogenesis.

Growth and reproduction are two biological processes that are tightly regulated and linked in all animals. Sexual maturation and reproduction takes place after the animal has reached a certain size or age and then the ingested energy is directed towards production and maturation of gametes. Steroid hormones play a crucial role in both processes and crosstalk in terms of regulation is crucial. A reproductive hallmark in females is transcription of vitellogenins (Vg). Vgs are produced in large quantities and requires access to sufficient nutrients. It has been shown that nutrient sensing and hormone regulation are linked to induce and regulate Vg production in mosquitoes e.g. [9]. In insects like *Drosophila* and *Aedes* 20E-signaling through EcR-USP induce transcription of key yolk proteins like vitellogenins in adult females. In the red flour beetle (*Tribolium castaneum*) it has been shown that Juvenile Hormone (JH) is required for vitellogenin synthesis and that 20E-influenced oocyte maturation [10,11]. In mosquitoes it has been shown that target of rapamycin (TOR) plays a central role in mediating the nutritional signal from amino acids and this signal is required for initiating production of Vgs [12].

Crustacea is a large group of arthropods containing diverse groups such as malacostraca, brachiopoda and maxillipoda. Whereas the endocrine system has been well studied in the large malacostracans (e.g. crabs, lobsters, shrimps) limited knowledge exist for most of the other groups like the copepods. In a recent review by Nagaraju [13] the reproductive regulators in decapod crustacean were summarized. Ecdysteroids are present in decapods and their main function is related to growth (molting) and gonadal maturation.

The salmon louse (*Lepeophtheirus salmonis*) is an economical important parasitic copepod in the salmon aquaculture [14]. In addition to direct cost to the farming industry release of lice larva to the environment is an undesired environmental effect of salmon sea ranching [15]. The negative effects have spurred molecular research on the salmon louse in the last decade [16-22] and in recent studies we have examined the sexual maturation in female lice [18] and characterized the major egg-yolk proteins [16,17].

Here, in order to better understand the basic features of female reproduction in copepods, we have characterized an RXR type of nuclear hormone receptor from *L. salmonis* (LsRXR). Five different transcripts encoding an RXR type NR were identified and we have used RNAi to knock-down these transcripts in adult female lice to investigate its role in reproduction. Knockdown of LsRXR in adult female lice resulted in basically abolished larvae production. To further understand the significance of LsRXR in adult female lice we investigated transcription levels in these knock-down lice on a *L. salmonis* microarray with app. 9700 different transcripts. Transcription of a large number of genes was affected upon knocking-down LsRXR by RNAi demonstrating its crucial role in copepod reproduction. Although copepods and insects are distantly related arthropods the present study points to several similarities in RXR (USP) functions.

## Results

### Cloning and sequence analysis of LsRXR

**Figure 1:**
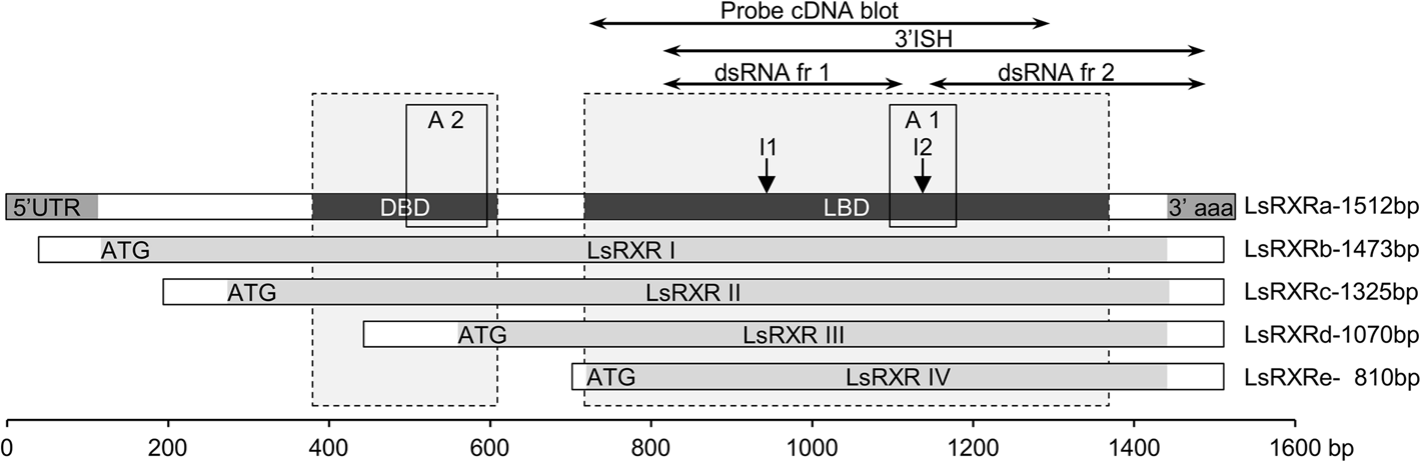
**Overview over the different forms of LsRXR.** The five different cDNAs (LsRXR a-e) shown in grey encode for four putative proteins (LsRXRI-IV). The two longest forms contains both, the DNA binding domain (DBD) and a ligand binding domain (LBD), while the two shortest transcripts only contain an intact LBD. The positions of the two fragments (fr) used for the RNAi (dsRNA fr 1 and dsRNA fr 2) are shown as well as the position of the *in situ* probe at 3′ end (3′ISH probe), the position of the cDNA blot probe (Probe cDNA blot), the two introns (I1 and I2) and the two Q-PCR assays, assay1 (A1) and assay2 (A2). The position of the southern blot probe is the same as that for dsRNA fr 2.

During our EST-sequencing effort we identified one clone with significant Blast hits with RXR/USP nuclear receptors from invertebrates. This clone was re-sequenced and 5′-RACE was performed to obtain the full length cDNA. The RACE experiment revealed five different transcripts ranging from 810 to 1512 bp (Figure 1). To further validate if there were more than one transcript we conducted a cDNA blot using mRNA from different developmental stages. The cDNA blot showed the presence of at least two different transcripts (see Additional file 1: Figure S1). The longest sequenced transcript was named LsRXR and contains a single open reading frame encoding a putative protein of 442 AA. We identified two introns in the genomic sequence of the LsRXR, but the different cDNA lengths obtained in the RACE experiment could not be linked to these. We performed a Southern blot with an LsRXR specific probe and only one band was detected (data not shown). The lack of additional copies of LsRXR in the salmon louse genome is further strengthened by scrutiny of assemblies of the salmon louse genome that is presently being sequenced.

BlastP search with the deduced protein sequence gave very strong hits with members of the nuclear hormone receptor NR2 family, particularly USP and RXR receptors from different arthropods species. The LsRXR protein does not contain a signal peptide (/www.cbs.dtu.dk/services/SignalP/).

LsRXR has all the typical features found in nuclear hormone receptors, including a well conserved DNA binding domain (DBD) and a ligand binding domain (LBD). The LsRXR has a unique row of 10 Asp residues just after the T-box [23] which to our knowledge is not present in any other species (i.e. no other similar sequences deposited in GenBank (nr, dbEST and wgs) except *L. salmonis* has this Asp repeat). According to the predictions made by Devarakonda, et al. [23] this region is an α-helix. We compared the predicted structure for the region containing the 10 Asp residues (from aa 155 – 200 LsRXR numbering) using the *Drosophila* USP sequence and LsRXR obtained by PSIPRED application (http://bioinf.cs.ucl.ac.uk/psipred/). The Drosophila USP showed a similar structure as determined by Devarakonda, et al. [23] whereas the predicted structure of LsRXR showed two helixes interrupted by a coil region, created by the Asp residues.

Our RACE experiment revealed five different cDNAs (i.e. 810 bp, 1070 bp, 1325 bp, 1473 bp and 1512 bp omitting the polyA tail). The three longest cDNAs contains complete DBD and LBD domains upon translation. In contrast, the two shortest cDNAs encodes a partial or no DBD and a complete LBD. The cDNA blot revealed two distinct bands (about 800 bp and 1500 bp) and indications of additional bands between these two main bands (see Additional file 1: Figure S1) that correlate with the length of clones obtained from the RACE experiment. The dsRNA fragments (fragment 1 and fragment 2) used in the RNAi experiment described below are both located at 3′ end to obtain knock-down of all forms.

### Phylogenetic analysis

**Figure 2:**
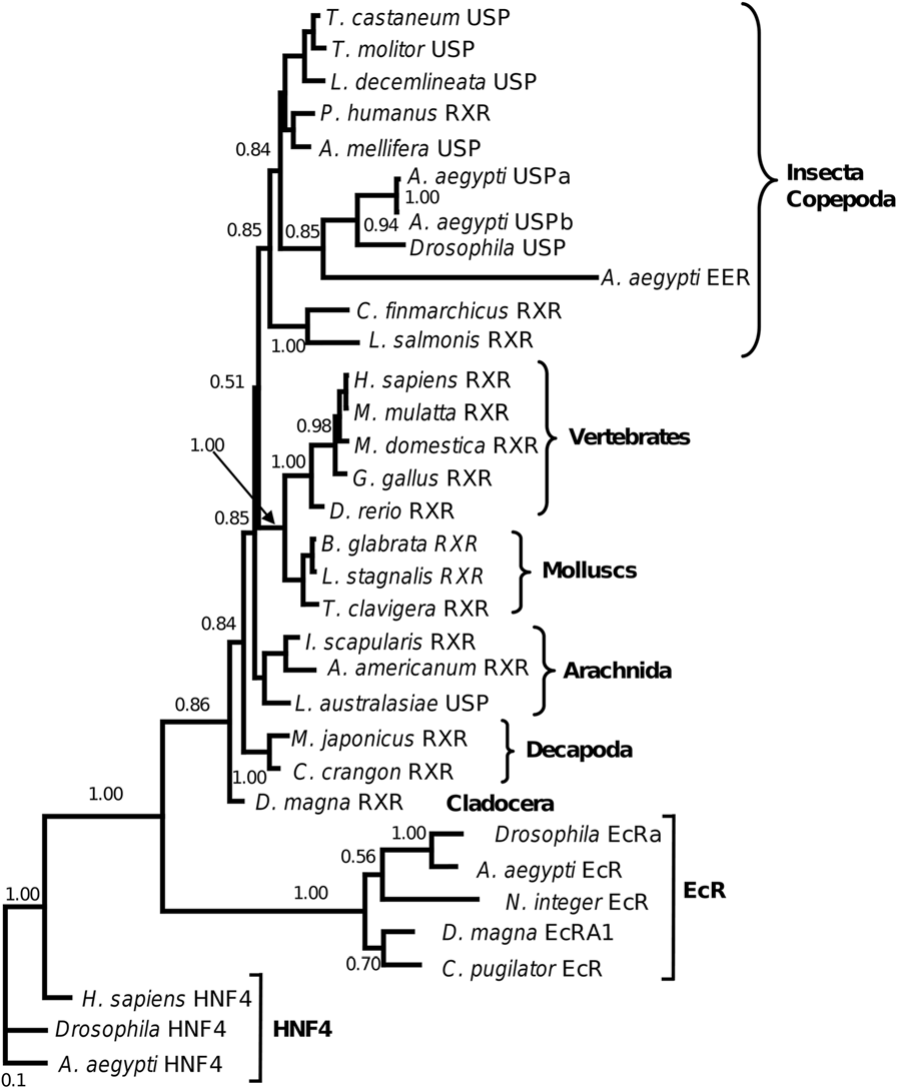
**Phylogenetic tree obtained after the byesin analysis.** The two included copepod species (i.e. *L. salmonis* and *C. finmarchicus*) occur as a sister group to the insects. We did not obtain evidence for monophyly for the arthropods since decapods and cladocera grouped outside the insect/copepod clade.

We conducted a phylogenetic analysis to investigate the relationship between LsRXR and other homologous sequences from a diversity of metazoans and the results from the Bayesian analysis is shown in Figure 2. In addition to USP/RXR sequences we included some EcR and Hepatocyte nuclear factor 4 (HNF4) proteins in our initial analysis. Like USP/RXR HNF4 also belongs to the nuclear subfamily 2, while EcR belongs to the nuclear receptor subfamily 1. The phylogenetic tree construction showed that all included USP/RXR occur within one group with good branch support (Figure 2) with the EcR as a sister group. In the USP/RXR group, vertebrates and mollusks occur as a monophyletic group and a sister group to the arthropods but it was not well supported. The USP/RXR from the included arthropods did not create a monophyletic group and the tree puzzle analysis gave poor/no significant support values for most of the basal nodes. To further asses the phylogenetic relationship between the USP/RXR sequences we removed all the HNF4 and EcR sequences and performed a new phylogenetic inference with only these sequences. This gave a significant support for the arthropod USP/RXR but the internal branching pattern did not obtain significant support in the tree puzzle analysis (data not shown). LsRXR grouped together with a putative RXR from the free living copepod *Calanus finmarchicus* in all our analysis. In none of our analyses did the copepods group together with the other included crustacean RXR sequences. The sequences annotated as USP did not form a monophyletic group in any of our analyses and to what extent USP should be used in initial annotation for this type of arthropod sequences could be questioned.

### Localization of LsRXR transcription by in situ hybridization

**Figure 3:**
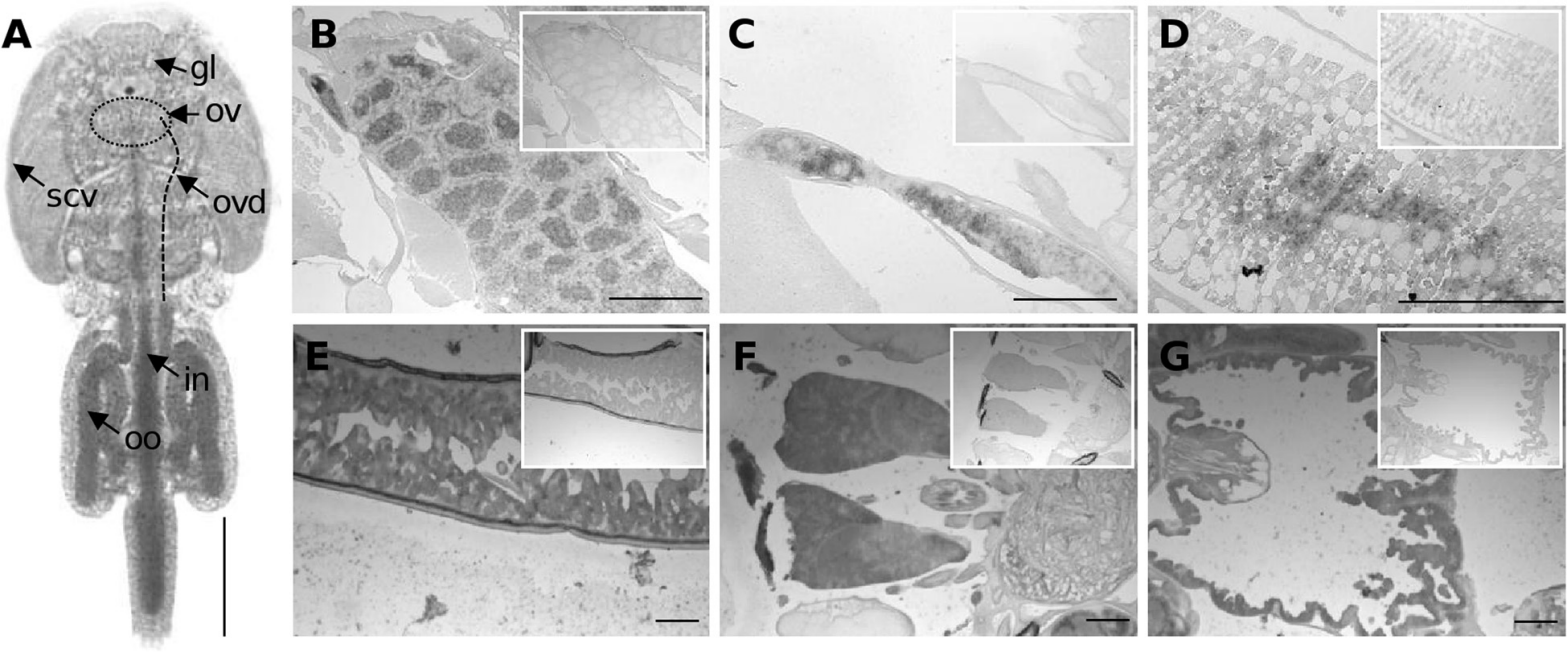
**Localization of** ***L. salmonis*** **RXR (LsRXR) transcripts in adult female salmon louse. (A)** Light microscopy of mature female indicating where the different tissues in the lice are located (gl = gland. Ov = ovary, ovd = oviduct, scv = subcuticular tissue, in = intestine, oo = oocytes). Figs. B to G shows hybridization using antisense probe (large picture) and sense (i.e.negative control) (small insert) of comparable sections. **(B)** shows positive hybridization in the ovary. The signal was detected in the lumen of the ovary tube. In **C**, a positive signal is detected in the cytoplasm of the immature oocytes in the oviduct and the more mature vitellogenic oocytes **(D)** in the genital segment are also positive. LsRXR is transcribed in the subcuticular tissue **(E)** and in several glands **(F)** and the intestine **(G)**.

In order to identify where LsRXR is transcribed in adult female lice we used a RNA probe for in situ hybridization. The probe was made in the 3-prime end of the transcript and would hybridize with all the different length variants of LsRXR. LsRXR is transcribed in several different tissues and cell types (see Figure 3). In the ovarium, LsRXR was detected in the lumen of the ovarium tubules (Figure 3B), in the oocytes after they have entered the oviduct (Figure 3C) and in the vitellogenic oocytes in the genital segment (Figure 3D). The subcuticular tissue also stained positive for LsRXR mRNA (Figure 3E) and the entire tissue transcribes LsRXR in adult females. LsRXR transcripts were also detected in the intestine (Figure 3G) and in some glands (Figure 3F). We also tempted to use an in situ probe localized in the 5′-region of LsRXR (i.e. a probe that would only identify the largest variant; located at 119-626bp) but we could not obtain a signal (data not shown). A likely reason for this would be that the longest transcript was present at an abundance below the detection limit for our in situ assay.

### Transcription of LsRXR in different developmental stages of *L. salmonis*

**Figure 4:**
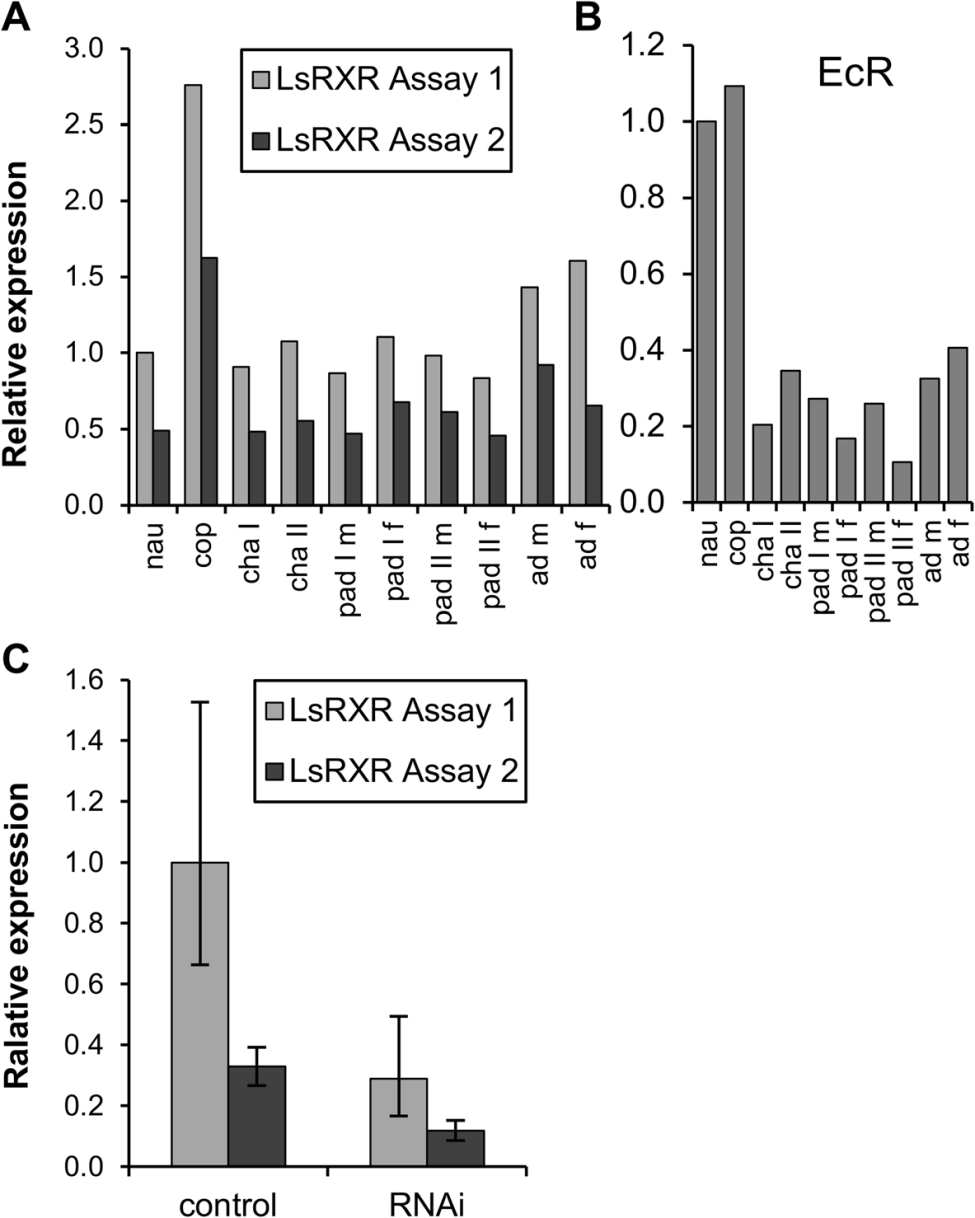
**Transcription of LsRXR in different developmental stages.** Two real time PCR assays were used where assay 1 measures all forms of LsRXR whereas assay 2 only measures LsRXR forms containing DBD and LBD (see Figure 1). **A)** LsRXR is highest transcribed in copepodids (all forms). The longest forms (i.e. those having complete DBD and LBD) comprise about 50% of the total LsRXR in all developmental stages examined. **B)** Expression pattern of EcR in the same samples as measured in A. **C)** Quantitative real time PCR demonstrates significant (T-test: p < 0.02) knock-down of LsRXR in the salmon louse.

We set up quantitative real time PCR to quantify the LsRXR transcription in key developmental stages in the salmon louse. Since there are two main types of LsRXR transcripts present, one type with a complete DBD and one type with incomplete (or lacking) DBD we made two real time PCR assays where assay 1 takes up all LsRXR forms and assay 2 only takes those with DBD (see Figure 1 for detailed location of the assays). LsRXR assay1 shows expression of all forms, while LsRXR assay2 shows the three longest forms only. As expected LsRXR assay1 being a sum of all forms of LsRXR, was expressed highest (Figure 4A). The longer forms were detected at levels of about 50% (between 41 and 64%) in all developmental stages. The shortest form, which did not include the DNA binding domain, was thus expressed about as high as all longer forms put together. Comparing all included developmental stages, strongest expression of LsRXR was found in free living copepodids. Apart from that it is expressed quite equally in all stages. We also identified EcR in the salmon louse draft genome sequence and developed a real time PCR assay for this key gene for comparative purposes (Table 1). While LsRXR is only expressed significantly higher in copepodids, EcR is expressed at the same level in nauplia and copepodids and shows more variability in the other stages (Figure 4B).

**Table 1:** **Primer- and probe sequences of TaqMan assays used for Q-PCR analysis**

**Transcript**	**Forward primer**	**Reverse primer**	**Probe**
LsRXR (assay1)	CCTAGTTGAACTCATCGCCAAAATG	TGAAGAGTATGATGGCTCGTAGACA	CCGCTTTGTCCATTTGCAT
LsRXR (assay2)	GCAGAGAAAATCGGGATTGTGTGAT	GCTTCTCTCTTCATTCCCGTATCG	CTGTCGCTATATGAAATGT
LsEcR	ACAAACAAAATGAATGCACAAAGTCCTT	TTAAAAACCGAAAATTGCCCCCATT	ATGTCCCAAATTTCC
Cl333Contig1	CTGCAACTATGCTGAAGACTCTTTG	ATTCAACGGCACCATAGAGAGATG	CCATGCTCTGAATCTC
Cl3250Contig1	CGCTTTGGTGCGCATGA	GGAGACAGACATAAAACTTGGAGCAA	TCGTCTTGCACATCCC
Cl3400Contig1	GCAACCTTGGCATCGATTACAATTA	TGCAAAAGGACCTCGAGATCATTG	CCCCTCTCCACAAACT
LsVit1	ACATCGACTACAAAGGAACTCAGAAC	GGAAGCATGTAACGAATGAACTCA	AGATTTTCTTTAGCTTCTGGATACAAACCTGCTCCA
LsVit2	AATGAGCAATTTAGTTGAGAAAACTTGT	CAATCTCGCTTTGAGCATCACA	TGGATAAATCACGTCAAGTTAGTTACCCTACCGC
LsYAP	GGCTTTAGGCTCTGATGTA AGCA	TGTTCACAAGTAAAAGCAGTGTCATTT	TCAATCACGATGGACCATTCACTGTTTTTG
LseEF1a	CATCGCCTGCAAGTTTAACCAAATT	CCGGCATCACCAGACTTGA	ACGTACTGGTAAATCCAC

The location of the different LsRXR assays (assay 1 and 2) in the gene is shown in Figure 1.

### Functional assessment of LsRXR by RNAi in adult female lice

In order to assess the function of LsRXR in adult female salmon louse, we performed an RNAi experiment injecting dsRNA from two different regions of LsRXR (see Figure 1) into newly molted adult female salmon lice (50 in each group) and a control group (50 lice) receiving dsRNA encoding a fragment of trypsin from Atlantic cod (see [16]). When the experiment was terminated a similar number of lice were recovered from each group (see Table 2 and Additional file 2) indicating that the effect on the adult females in the experiment was at a sub-lethal level. Down regulation of LsRXR by RNAi affected all variants (Figure 4C). Down-regulation for both fragments was on average 60% (Figure 4C, Table 2).

**Table 2:** **Summary of the results from knock down of LsRXR**

**Group**	**# female on fish at harvest**	**Length of egg strings (mm)**	**Hatching of egg (%)**	**# copepodites**	**Dead/deformed larvae (%)**	**Rel. expression of LsRXR (Q-PCR)**
**control**	38	16.8	80.5	412	1.4	1
		SD = 1.7, n = 25	SD = 12.8, n = 25	SD = 75, n = 18	SD = 1.8, n = 25	SD = 0.27, n = 18
**RNAiFr1**	38	8.8	13.3	5	84.5	0.37
		SD = 2.7, n = 35	SD = 20.5, n = 25	SD = 10, n = 20	SD = 24.9, n = 13	SD = 0.13, n = 29
**RNAiFr2**	39	8.3	9.1	1	85.0	0.47
		SD = 3.4, n = 24	SD = 8.6, n = 15	SD = 4, n = 15	SD = 29.1, n = 11	SD = 0.15, n = 19

Female lice were counted at the time point of harvesting. These are the remaining ones from the 50 female lice put on 6 fish in each tub after injection of double stranded RNA. Control group lice were injected with double stranded cod RNA (trypsin), RNAi Fr1 and Fr2 group lice were injected with double-stranded RNA fragment one or two (see Figure 1) respectively. The length of egg strings was measured at harvesting and the number of copepodids was reported at day nine after hatching. Photographs of harvested adult females and their egg strings are shown in Additional file 2: Figure S2. The two RNAi groups did not differ significantly in all measured parameters (all t-tests: p > 0.05) while significant differences between control group and the RNAi groups were evident in all measured parameters except number of female lice on fish (T-tests: length of egg strings: p < 1.98*10^−25^, hatching success p < 2.54*10^−25^, number of copepodids: p < 2.2*10^−14^, proportion of dead/deformed larvae. p < 9.7*10-14, relative expression: P < .1.7*10^−7^).

Table 2 summarizes the overall observations from the RNAi experiment with emphasis on egg production and the number of copepodids produced from the three different groups. The RNAi experiment clearly demonstrated that LsRXR is crucial for both egg production, embryogenesis and larval development since the number of produced eggs as well as development to copepodids were severely affected in both groups treated with dsRNA from LsRXR. Egg-string length in the LsRXR RNAi-lice was about half of that in control lice. In addition, the lice in both RNAi groups produced eggs with significantly decreased hatching success and a high proportion of the larvae that hatched were strongly deformed, resulting in close to zero normal copepodids (Table 2).

**Figure 5:**
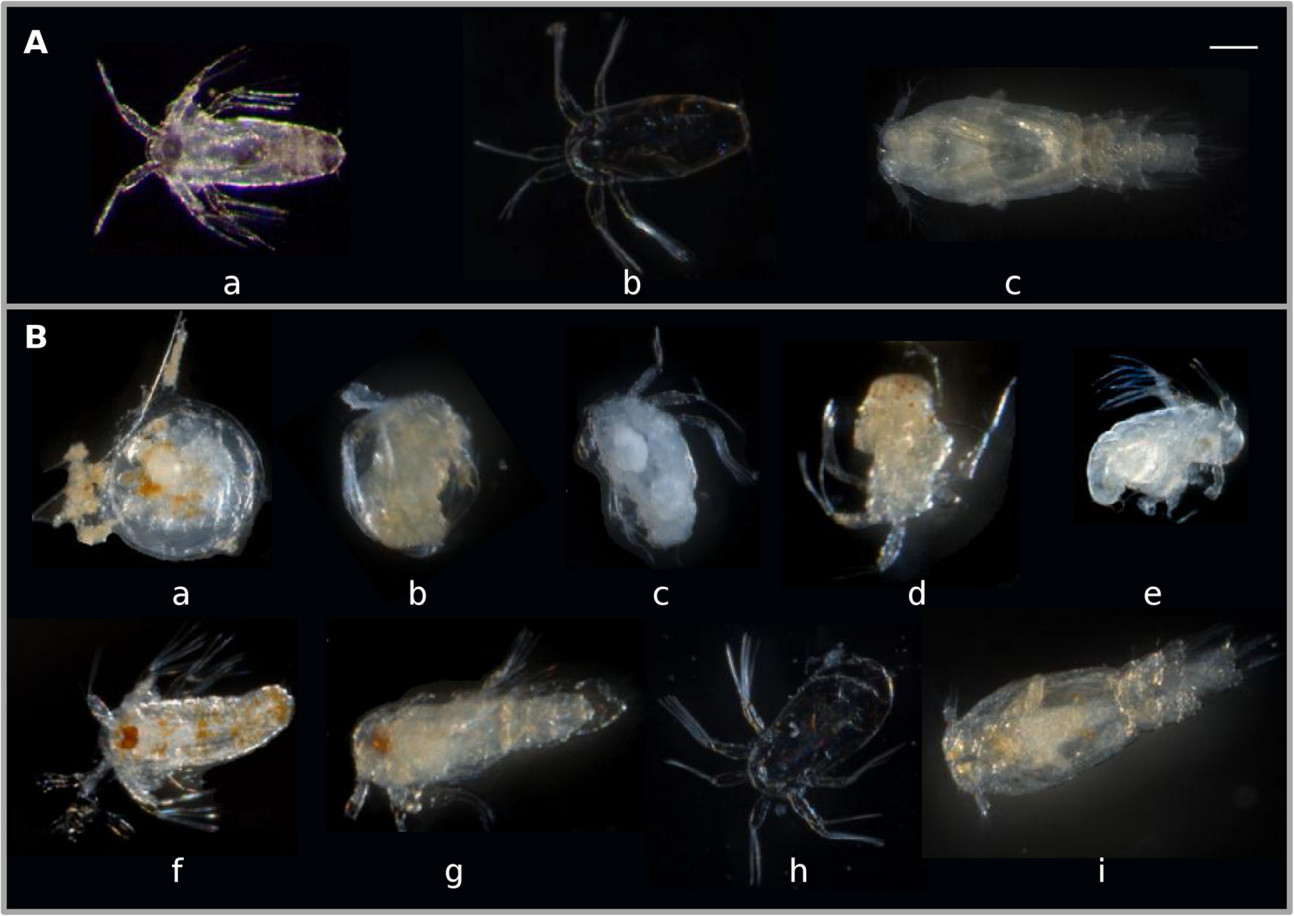
**Phenotype of salmon lice offsprings obtained after the LsRXR RNAi experiment.** In the control group **(A)** normal developed nauplia II (a) and copepodids (c) are found. At day nine the exuviae from nauplia II (b) are found in large amounts. In the RNAi groups development is disrupted, either by disrupted hatching **(Ba)**, or the nauplia are deformed **(B b-f)**. We also observed nauplia arrested in their development to copepodids (Bg). In a very few cases apparently normal looking copepodids (Bi) or exuvia (Bh) were observed. The bar in the upper right corner is 0.1 mm long.

A selection of representative phenotypes from offspring’s observed after the RNAi is displayed in Figure 5. In the control group, only 2.1% of the larvae were dead and no deformed larvae were found. In the LsRXR RNAi groups around 80% of the larvae that hatched were deformed or dead. A sign of normal molting between the larval stages is presence of normal exuvia (Figure 5 Ab). In the LsRXR RNAi groups normal exuvia were extremely rare indicating molting and developmental abnormality (Figure 5B c, d). In some cases a few apparently normally developed copepodids could be detected.

**Figure 6:**
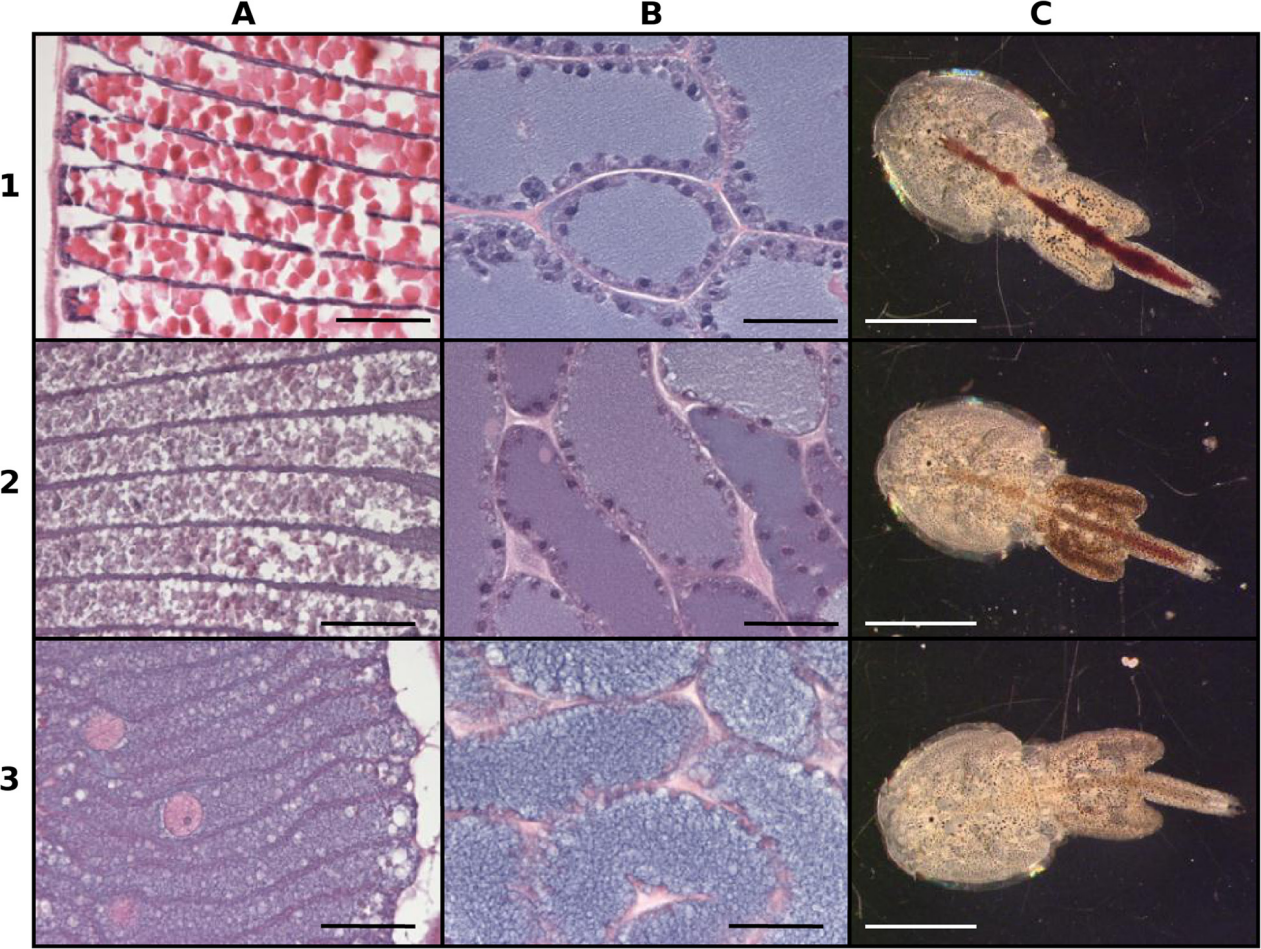
**Histological assessment of LsRXR RNAi.** HES (Haematoxylin-erythrosin-saffron) stained section (3μm) through oocytes **(A)** and ovary **(B)** of control lice (row 1) and LsRXR RNAi (row 2 and 3) (scaling bar: 50μm). (2) shows the section through a very mature louse (egg strings had started to hatch at time point of sampling, full genital segment) with relatively low RNAi effect (some copepodids hatched) while (3) shows a section through a strongly effected louse (no hatching of eggs). Note the less organized oocytes with no distinct vitellin crystals and the ovary did not contain any oogonia. The whole lice corresponding to sections in each row are shown in **(C)** (scaling bar: 5 mm).

To further assess the effects LsRXR RNAi has in adult female lice we sectioned lice to identify histological abnormalities. Two typical lice from the two types of RNAi lice were selected, type I representing female lice with a filled genital segment that produced eggs which hatched but produced larvae that developed poorly, and type II representing female lice with a relatively empty genital segment that produced eggs which did not hatch. The histological examination of the type I louse revealed less organized yolk granules in the maturing oocytes in the genital segment and indications of fewer oogonia in the ovary (Figure 6A2 and B2). In the type II louse the histological examination revealed more severe changes in the ovarium and the maturing oocytes in the genital segment. No oogonia could be observed in the ovarium follicles (Figure 6B3) and the follicular substance was less dense and filled with vacuoles. The oocytes present in the genital segment were highly atypical and irregularly shaped (Figure 6A3). The typical vitellin crystals [16] as seen in Figure 6A1 could not been observed.

### Egg laying cycle

Under the given conditions, it takes about nine days for the oocytes to mature inside the genital segment and to be extruded as a new pair of egg strings. As expected the harvested female lice were not synchronous in their egg laying cycle which is visible as differences in the thickness of the immature oocytes. A more precise measure is how many days it takes for the previous egg string pair to hatch, as egg strings are fertilized when they are extruded from the female lice and they are attached to the lice until hatching. By incubating the egg strings from lice to be analyzed, the time to hatching can be used as a measure for how mature the new oocytes in the genital segment of a female louse are. The shorter the time to hatching, the more mature the oocytes are. For further explanation see Additional file 3: Figure S3.

For the control group, the number of days to hatching for the harvested egg strings was five, six, seven, or eight days. In the RNAi group there was more diversity regarding the egg laying cycle and lice were divided into four different groups, depending on hatching time after harvest (hatching prior to harvest, early hatching, late hatching or no hatching).

### Functional assessment of LsRXR by microarray analysis

**Figure 7:**
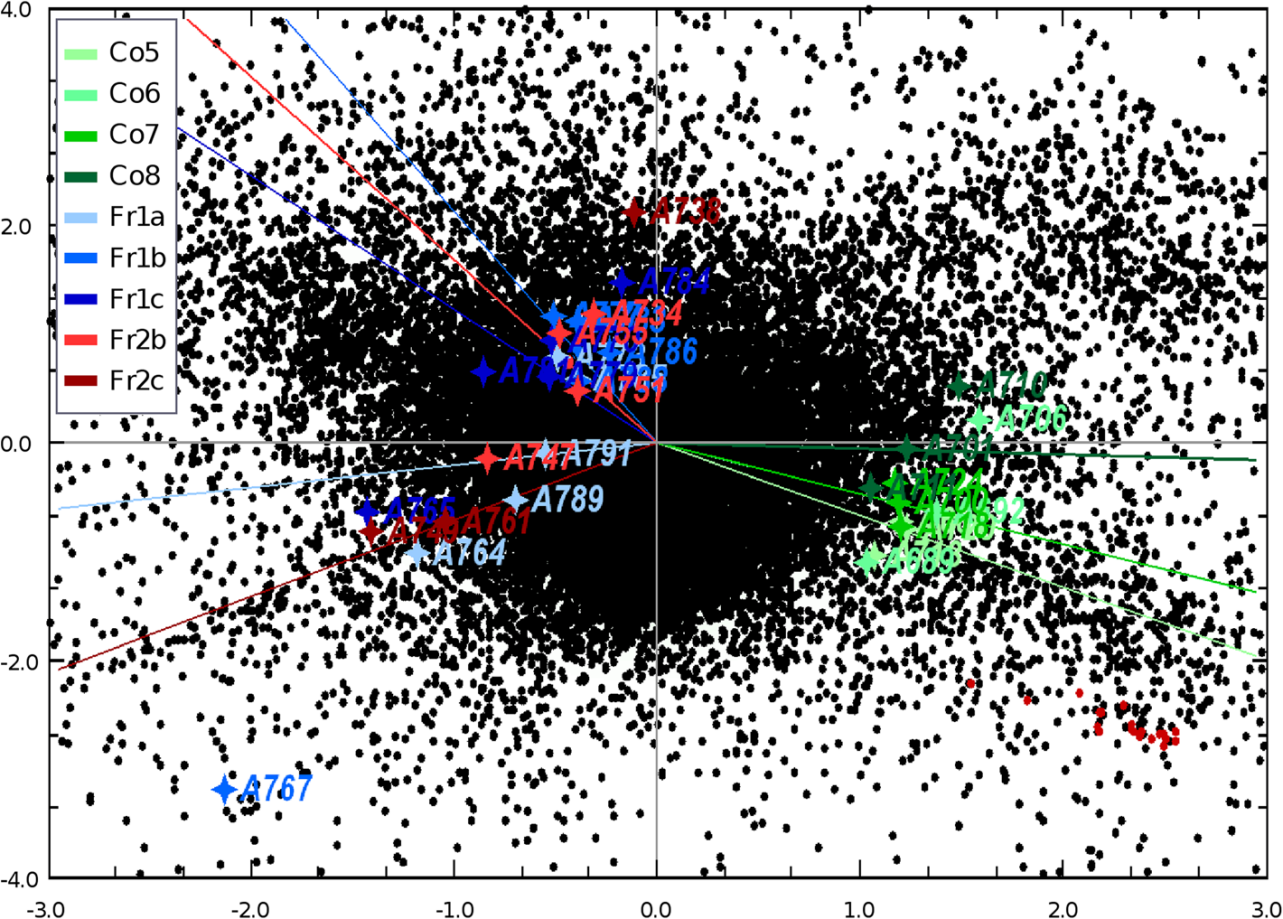
**Correspondence analysis plot.** Spots represent individual oligos and vectors represents sample group means. The red spots are the oligos created from the LsRXR sequence. Genes represented by oligos located near the plot origin are poorly correlated with any of the sampled groups. Oligos located further from the origin are stronger expressed in samples located in same direction and lower expressed in samples located in opposite direction. The distribution of single samples (marked with a star and an A-number) is shown in the color of the group (see legend) the sample belongs to. Single samples are grouped according to the fragment injected: fragment one (Fr1), fragment two (Fr2), control (Co) and according to position in the reproductive cycle. The control group is divided into Co5 to Co8 representing lice with eggs that hatched after five, six, seven, eight days after harvesting respectively. Fr1/Fr2 a, b, c representing lice injected with fragment one or two respectively hatching early (one to two days after harvesting) (a), late (six to nine days after harvesting) (b), or did not hatch (c). For further explanation of the position in egg laying cycle see Additional file 3: Figure S3.

To further gain information about function to LsRXR we conducted a microarray experiment using a 44K oligo-based microarray where we compared lice injected with dsRNA from LsRXR (the two different fragments) and lice injected with dsRNA from a control gen (cod trypsin). The 44k Agilent salmon louse oligo-microarray represents approximately 9740 different transcripts obtained from ESTs generated from a wide diversity of cDNA libraries (see Table 3). Correspondence analysis (CA) clearly demonstrated that the control group and the LsRXR RNAi group have distinct transcriptomes (Figure 7). As expected, RXR is down regulated in the RNAi lice. The oligos representing LsRXR (Figure 7 red spots) are strictly correlated to the control lice. The transcriptomes from the two different dsRNA fragments for LsRXR were completely overlapping. Furthermore, we could not identify any differences in transcription pattern related to egg maturation for the included lice (see above). In general, gene expression is more uniform in the control group than in the RNAi group (louse A767 seems to be an outlier) probably reflecting the variation in down regulation.

**Table 3:** **Libraries sequenced to yield the ESTSs used to design the 44k oligo array**

**Name**	**Type**	**Stage**	**Sex**	**# EST**	**% of all EST**
SB*	Subtracted	Adult	Female/male subtracted	135	0.4
FB*	cDNA	Colon intestine adult	Female	502	1.6
ME*	cDNA	Adult with blood	Female	3663	11.8
**L1T**	cDNA	T1 adult1	Female	1492	4.8
PU*	cDNA	Preadult II	Female	586	1.9
**LF**	cDNA	With filament, preadult I, preadult II	Female, male	1784	5.7
**LPU/LPA**	cDNA	Preadult stages	Female, male	1351	4.4
**CC**	cDNA	Chalimus 3	Female, male	117	0.4
**NA**	cDNA	Nauplii	Female, male	61	0.2
HA*	cDNA	Adult	Male	646	2.1
**LNO**	Normalized	Preadult I/II, adult, with filament preadult I/II	Female, male	14377	46.3
**LNC**	Normalized	Copepodite	Female, male	3217	10.4
**NLG**	Normalized	Egg strings	Female	3118	10.0

Libraries marked with * were used on a former cDNA array. [18].

Based on the microarray analysis LsRXR was down regulated 2.2 times for fragment 1 (n = 19) and 2.0 times for fragment 2 (n = 7). The knock-down of LsRXR was further confirmed by Q-PCR and showed a down regulation of 2.9 times for fragment 1 (n = 27) and 2.3 times for fragment 2 (n = 18) (see Additional file 4: Table S1). To further validate our microarray data we conducted Q-PCR on six regulated genes (three up regulated and three down regulated). These data confirmed the results from the microarray data (see Additional file 5: Figure S4). Expression of these six genes was also shown in other developmental stages to illustrate the effect of down- or up regulation compared to other developmental stages.

### Differentially expressed genes identified by significance analysis of microarrays (SAM)

We conducted a SAM analysis of the data derived from microarray analysis to identify differentially expressed genes between the LsRXR RNAi and the control group. A total of 247 genes were significantly down regulated and 552 genes up regulated upon knock-down of LsRXR (see Additional file 4: Table S2 and S3).

Of all 9740 different transcripts on our salmon louse array only 54% have a hit in UniRef and a large proportion of these hits give “unknown functions”, resulting in only 16% with a GO annotation. In our two sets of up- or down regulated genes about 60% have a hit in UniRef (61.8% for the down regulated transcripts and 60.5% for the up regulated transcripts) and about 20% have a GO annotation (23.5% for the down regulated and 19.8% for the up regulated transcripts). Nevertheless there is a clear enrichment of particular GO-groups in the two sets of up- or down regulated transcripts (see Additional file 4: Table S4). In the set of down regulated genes, lipid metabolism and lipid transport is a hallmark (see also Additional file 4: Table S5). In addition, yolk proteins are strongly down regulated (see Additional file 4: Table S2). Many metabolic processes (see Additional file 4: Table S4) were among the up regulated genes and they created a distinct signature. In particular many transcripts encoding ribosomal proteins and cuticle related transcript were up regulated (see Additional file 4: Table S3 and S4).

**Figure 8:**
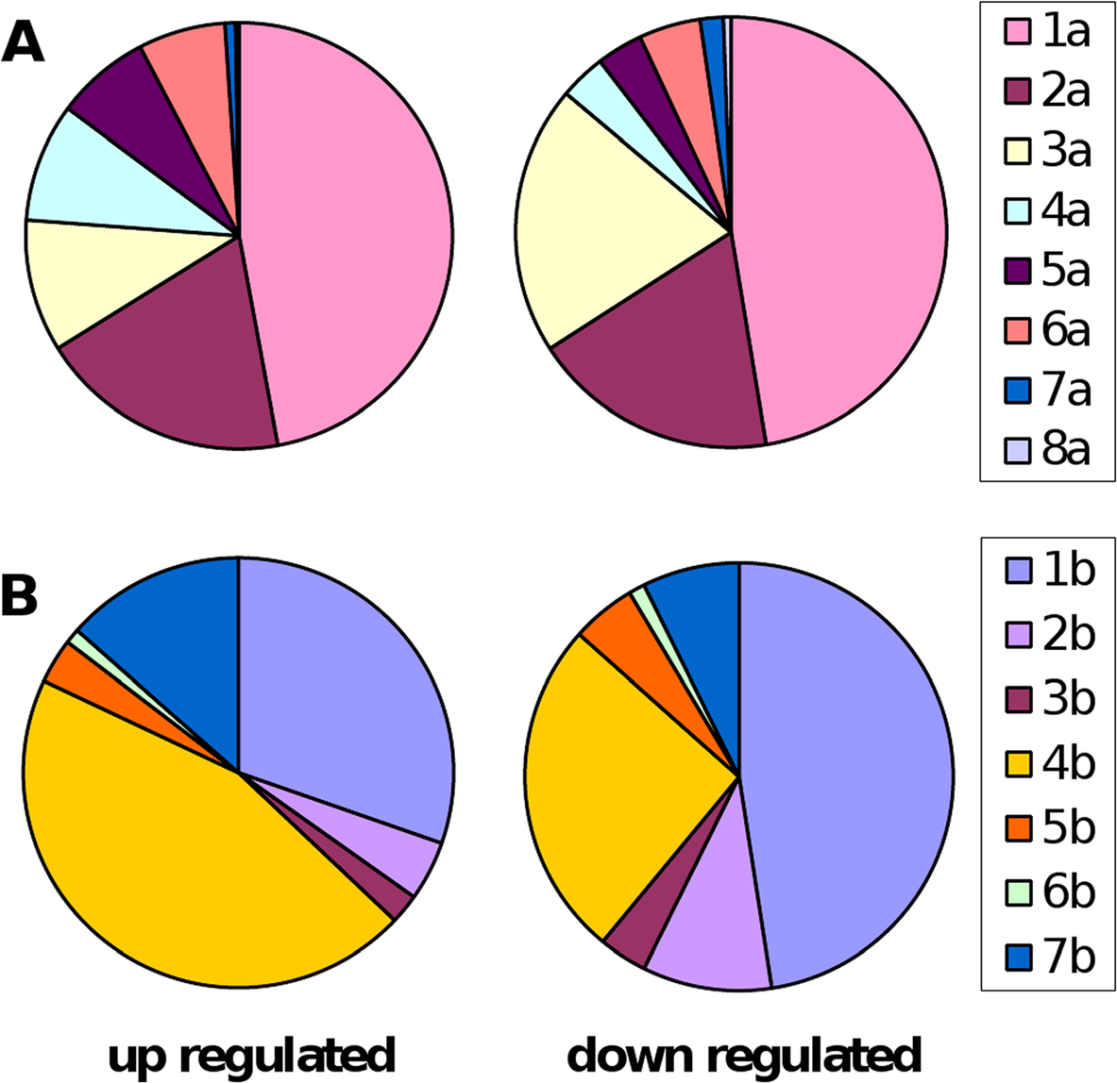
**Origin of ESTs from different libraries (for libraries see Table** 3 **).** 32% of the ESTs from the up regulated genes or 36% of the ESTs from the down regulated genes originated from the normalized library LNO, made from different developmental stages. In **(A)** origin of ESTs from other libraries than LNO is shown; 1a: early developmental stages; 2a: early developmental stages plus mature adult female; 3a: mature adult female; 4a: young and adult forms with males; 5a: young and adult forms, no male; 6a: immature adult female; 7a: adult female (mature and immature); 8a: adult (female/male). The largest share is the early developmental stages (1a). The early developmental stages are split up in **(B)**. 1b: copepodid; 2b: egg, copepodid; 3b: copepodid, immature adult female; 4b: preadult; 5b: preadult, immature adult female; 6b: egg, immature adult female; 7b: different young developmental stages.

Since a large number of the regulated transcripts on the microarray have no or poor annotation we conducted an analysis where origin of the probe sequences were considered (i.e. what developmental stage the cDNA sequences were isolated from). If the probes of interest are from one specific library (i.e. developmental stage) this may give indications towards function. Although many of the regulated transcripts originated from early developmental stages (Figure 8A, B) a clear difference was observed between the up and down regulated gene sets. The down regulated gene set was dominated by transcripts from the copepodid cDNA library (Figure 8B) and mature adult females (Figure 8A). For the up regulated genes an increased frequency of transcripts originating from preadults (Figure 8B) was evident. This is also seen when comparing only those transcripts originating from one library only (Additional file 5: Figure S4).

#### LsRXR regulates egg yolk protein transcription

Knock-down of LsRXR has a strong effect on transcription of the main egg yolk proteins and its regulators in the salmon louse. All three main egg yolk proteins (LsVit1, LsVit2 and LsYAP) are strongly down regulated in the present study and this was confirmed by Q-PCR (see Additional file 6: Figure S5A). A carboxypeptidase, similar to vitellogenic carboxypeptidase (VCP) in mosquitoes [24] is also strongly down regulated in the present study. In insects GATA transcription factors are regulators of vitellogenin transcription and it has been shown that the Vg-genes have binding sites for GATA factors ([25,26]). A putative GATA factor is up regulated in the present study pointing towards inhibition/down regulation of Vg-transcription. In the soft tick (*Ornithodoros moubata)* the Vitellogenin gene contains putative binding sites for GATA and the transcription factor Tramtrack [27]. A putative Tramtrack is 2.8 times up regulated in the present study. Lysosomes have been shown to be involved in vitellogenin regulation. In the mosquito fat body activity of some lysosomal enzymes are responsible for the termination of vitellogenin secretion by degrading organelles involved in the biosynthesis and secretion of vitellogenin [28]. In the present study genes with GO annotation lysosomes were in general up regulated (see Additional file 4: Table S4). A transcript with significant similarity to Neuroparsin was strongly up regulated (>1000 times) after LsRXR knock-down (Additional file 4: Table S2). In desert locust (*Schistocerca gregaria*) knock down of Neuroparsin resulted in increased Vg production [29].

#### Growth related processes are up regulated upon knocking-down of LsRXR

##### Cuticle proteins are up regulated

One of the signatures in the microarray assessment was a relatively large number of up regulated putative cuticle proteins. A total of 22 putative cuticle proteins were up regulated in female lice after knock-down of LsRXR (see Additional file 4: Table S2). Nine of these were strongly up regulated (>100 times). The transcription pattern for two of these (i.e. CL333Contig1 and Cl3250Contig1) was confirmed by Q-PCR (Additional file 5: Figure S4B). We also examined the transcription level of these two transcripts in several other developmental stages and for CL333Contig1 the transcription levels are high in most developmental stages compared to adult female lice receiving ds LsRXR RNA (see Additional file 5: Figure S4B). Furthermore, three chitin metabolism proteins are also up regulated. One transcript with hit to the barnacle cypris larva-specific gene *bcs-1*, a structural constituent of cuticle, is down regulated. In barnacle this is a gene expressed early after hatching [30]. Another structural protein, the Collagen α-1(XI) chain precursor isoform 1 transcript is up regulated (27 times).

##### Metabolic processes are up regulated

Based on GO annotation, metabolic processes are overrepresented among the up regulated transcripts and 28 up regulated transcripts are involved in metabolism (amino acid and other amines, and nucleoside/nucleotide metabolic, carbohydrates) or glycolysis/gluconeogenesis.

##### Some peptidases are up regulated

A range of trypsin-like peptidases have been identified in *L. salmonis* previously (see [19,20,31,21]. Only one of the previously characterized trypsin-like peptidases (i.e. LsTryp4 see [20]) is regulated in the present study. LsTryp4, which is transcribed in the intestine, is down regulated (four fold) in the present study. However, several other peptidases including serine peptidases are regulated as a result of knocking-down LsRXR. Seven of these peptidases are up regulated whereas 13 are down regulated. Among the strongly down regulated is a carboxypeptidase resembling vitellogenic carboxypeptidase from mosquito (see above). In addition, two transcripts resembling serine type endopeptidase inhibitors are up regulated.

#### Early developmental processes are up regul*ated* upon LsRXR knock-down

Many genes involved in development were up regulated in the present study (see Additional file 4: Table S2 and S6). Genes involved in neuronal development are up regulated and among them the transcription factor Tramtrack p69, the Integrin beta-PS and the Hypoxanthine phosphoribosyltransferase 1. Nogo-B is a negative regulator of axon-axon adhesion and growth and this gene is weakly down regulated. A Cofilin/actin-depolymerizing factor homolog, involved in many developmental processes (e.g. axoneogenesis in *Drosophila*) is up regulated as well.

Genes for regulating various signaling pathways involved in developmental processes are up regulated in the present study. The tumor suppressor protein “lethal (2) giant larvae” is required for the regulation of Notch, a signal protein preventing differentiation which is involved in many developmental processes in *Drosophila*, is up regulated. “Moesin/ezrin/radixin”, a regulator of the Hippo/SWH signaling pathway, restricting proliferation and promoting apoptosis is down regulated. In addition, “sprouty-related EVH1 domain containing 2” is up regulated and this protein is important for multicellular organismal development by inhibiting growth-factor-mediated activation of MAP kinase.

Several genes involved in regulation of embryonic polarity are regulated as a result of LsRXR RNAi. A transcript similar to “longitudinal lacking protein” is up regulated. This protein together with transcription factor GAGA are required for maintaining the repressed state of target genes including the homeotic genes sex combs reduced and ultrabithorax (both important for anterior-posterior axis formation in *Drosophila*). However, in the present study ultrabithorax is not clearly regulated due to LsRXR RNAi (data not shown). In addition, a short gastrulation homologue is up regulated, which is involved in the zygotic determination of anterior/posterior axis. Furthermore, twinstar known for affecting several different development processes like establishment of planar polarity and female gonad development is up regulated. The transmembrane protein nessy is a highly conserved gene and is controlled by Hox genes during *Drosophila* embryogenesis. A homologue in *L. salmonis* is strongly down regulated in the LsRXR RNAi-lice.

### Several cellular and molecular processes are affected by LsRXR knock-down

#### Ribosomal proteins are up regulated

Knocking-down the LsRXR by means of RNAi affected a wide range of molecular and cellular processes. A hallmark was a relatively large number of transcripts encoding ribosomal proteins that were consistently up regulated. In our dataset 33 transcripts with significant hit to ribosomal proteins were up regulated (around 2.3 times) as a result of knock-down of LsRXR. In addition, nine transcripts for translation regulation proteins are two to three times up regulated (elongation factor 1α/β2 and γ, eukaryotic translation initiation factor 3 subunit, 5A F-1, G-1 and subunit 6). Four spermine synthase transcripts are about 15 times up regulated. Spermine, involved in nucleic acid packaging is found in ribosomes.

#### Apoptosis inducer are down regulated

In the present experiment 14 of the regulated genes are involved in apoptosis (Additional file 4: Table S7). The six down regulated genes are mainly apoptosis inducer while the eight up regulated genes are (if direction known) suppressors of apoptosis implying that apoptosis is restrained in the LsRXR RNAi lice. An exception is the death inhibitor diap2 (down regulated in the LsRXR RNAi lice) which is known to be ecdysone regulated and repressed in *Drosophila* at the onset of histolysis in larval salivary glands [32]. Apoptosis is often induced through a caspase dependent pathway. One of the enzymes inducing apoptosis by this pathway, the apoptosis-inducing factor 3, is down regulated while the two caspases (caspase 9 or 6) are not regulated.

#### Fatty acid metabolism and transport is down regulated

Among the down regulated transcripts a large number are associated with lipid/fatty acid metabolism and transport. Furthermore, two prosaposin like transcripts, which are involved in lipid metabolism and transport are up regulated, but they probably act by isolating lipid substrate from the membrane, making them more accessible for degradation. A total of 14 transcripts involved in lipid metabolism and transport are down regulated (see Additional file 4: Table S3 and S5). Three of these are involved in lipid transport and five in fatty acid synthesis; among these is “Elongation of very long chain fatty acids protein” (UniProt: AAEL008004) which is a cholesterol responding acetoacetyl-CoA synthase. Six others are involved in metabolism and among them the sterol regulatory element binding protein 1 involved in regulating the transcription of the LDL receptor gene which is a key regulatory element in the promoter of several genes involved in cholesterol homeostasis. Several genes involved in steroid (including E20) synthesis or homeostasis are regulated in the present study (e.g. Niemann-Pick Type C-2, 7 dehydrocholesterol reductase, 24-dehydrocholesterol reductase).

#### Two ABC transporter are regulated

Many ATP-binding cassette (ABC) transporters and cytochrome P450 (CYPs) are target genes of RXR. Two putative ABC transporters are among the regulated transcripts (Additional file 4: Table S2, S3 and S8). One is strongly down regulated and this gene is involved in fatty acyl CoA transport. The other ABC transporter is up regulated and this transcript is a homologue to an ABC transporter involved in export of anions and drugs from the cytoplasm.

#### Genes involved in steroid biosynthesis are regulated

CYPs are either involved in development (e.g., retinoic acid or hormone metabolism, ecdysteroid biosynthesis) or in the metabolism of toxic compounds (such as heterocyclic amines or polyaromatic hydrocarbons). Of the eight different CYP which are regulated in the present study all except two are approximately four times up regulated. Three of them belong to the CYP3 group which is involved in drug and steroid metabolism. The other one is shadow (CYP315A1) with ecdysteroid C2-hydroxylase activity. In *Drosophila* this enzyme is known for synthesizing ecdysone from 2-deoxyecdysone.

### Assessment of known ecdysteroid target genes

**Figure 9:**
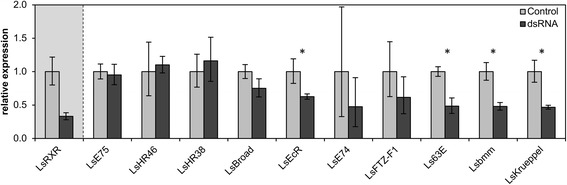
**Relative expression of genes not represented on the array in selected control- (light grey) and LsRXR knock-down-lice (dark grey).** Controls are used as calibrators. Confidence intervals (α = 0.05) are shown as error bars. LsRXR is down-regulated about three times by RNAi. Gene expression of genes known to be involved in the ecdysteroid cascade in other arthropods are shown: salmon louse forms of E74, 63E, EcR, HR46, HR38, E75, FTZ-F1, Broad, as well as the lipase Brummer, which is involved in the regulation of lipolysis in drosophila [33] and the gap gene Krueppel. Genes significantly down-regulated (T-test p-value < 0.05) in the knock-down group are marked with *.

Based on the preliminary genome assembly we identified and established Q-PCR assays for several of the genes known to be involved in the ecdysteroid cascade. We assessed the mRNA level of these genes in the LsRXR knock down lice in comparison to the control (see Figure 9). Of the included genes four of them were significantly down regulated in the LsRXR lice (i.e. EcR, 63E, bmm and Krueppel), see Figure 9).

### Comparative transcriptomics

In a previous transcriptomic study on salmon lice [18] gene regulation in immature and mature adult female lice was investigated. We examined the dataset from that study [18] and found that ten of the regulated transcripts are also regulated after RNAi knock-down of LsRXR (see Table 4). Six of these genes were down regulated in this study while four were up regulated. A putative cellular retinoic acid binding protein (i.e. FN1569 in Table 4) is among the up regulated transcripts. Currently there are several sea lice sequences in GeneBank with high similarity to this regulated transcript. However, to our knowledge no functional studies have been carried out to rule out the function or whether they actually can bind retinoic acid.

**Table 4:** **Overview over clusters and singletons regulated both by LsRXR knock down and during maturation of adult females [** 18 **]**

**Contig/singleton**	**Regulation in adult female lice (former study)**	**Regulation in this knock-down study**	**Annotation**
CL5Contig7	Down from T1-T6	Down regulated (3.8 times)	*Lepeophtheirus salmonis* clone LS0008 hypothetical protein mRNA, partial cds
CL5Contig8	Down from T1-T6	Down regulated (3.3 times)	*Lepeophtheirus salmonis clone LS0027 hypothetical protein mRNA, complete cds*
CL333Contig1	Down from T1-T6	Up regulated (30536 times)	Putative cuticle protein [*Lepeophtheirus salmonis*]
CL194Contig1	Down from T1-T6	Up regulated (2.3 times)	60S ribosomal protein L17 [Lepeophtheirus salmonis]
CL5Contig2	Down from T1-T6	Up regulated (3.9 times)	ABU41059.1 hypothetical protein [*Lepeophtheirus salmonis*]
CL12Contig1/2	Up from T1-T6	Down regulated (9 times)	vitellogenin 1 [Lepeophtheirus salmonis]
CL3Contig1/ FN825	Up from T1-T6	Down regulated (4.1 times)	vitellogenin 2 [*Lepeophtheirus salmonis*]
CL1Contig20	Up from T1-T6	Down regulated (1.55*10^24^ times)	LsYAP [*Lepeophtheirus salmonis*]
FN1569	Up from T1-T6	Up regulated (2.7 times)	Lepeophtheirus salmonis clone LS0038 putative cellular retinoic acid/retinol binding protein mRNA, complete cds
CL1632Contig1	Up only in preAII	Down regulated (3.2 times)	Lepeophtheirus salmonis clone PU676 hypothetical protein mRNA, complete cds

Arbeitman, et al. [34] examined transcription of *Drosophila* genes during the whole development cycle and identified genes expressed in different developmental stages. We compared the salmon louse genes to those identified by Arbeitman, et al. [34] with emphasis on reproduction and early development. We thus identified: early zygotic genes (one hit, not regulated), female germ line genes (58 hits, six up regulated), maternally rapidly degraded genes (19 hits, one up regulated), male and female germ line genes (two hits, none regulated), maternal rapidly reinduced genes (69 hits, four down-, two up regulated), maternal core germ line genes (49 hits, four down-, three up regulated), maternal gradual decline genes (67 hits, one down-, two up- regulated), genes changing during embryogenesis (49 hits, one down- two up regulated) and strict maternal genes (67 hits, two down-, three up regulated). Genes regulated in the present RNAi study are found in Additional file 4: Table S9). Most of these transcripts are only weakly regulated due to LsRXR RNAi. An exception is LNO3037 with significant hit to the zinc ion binding; nucleic acid binding protein FBpp0074410 with unknown function found in their table 13 (i.e. maternal gradual decline), which is over 100 times down regulated. LNO3037 has hit with a Novel KRAB box containing protein from *Mus musculus*. Among the up regulated transcripts four ribosomal proteins and other proteins involved in translation are present.

## Discussion

In salmon lice, LsRXR plays a crucial role in female reproduction as knock-down by RNAi resulted in close to zero viable offsprings. Although the female lice produced and extruded egg strings these eggs were either unable to hatch or produced severely deformed moribund larvae (see Figure 5). The LsRXR transcript levels were reduced about 2.5 times and this reduction had significant effect on transcription levels on a wide range of genes in the female lice. It is evident that these altered transcription levels influences a range of molecular and biological processes in the female lice and the consequences can be seen far into the embryonic and larval stages. The broad significance LsRXR has on gene expression of many functional different genes in *L. salmonis* could clearly be seen from our microarray assessment.

### LsRXR is a homologue of the insect nuclear receptor Ultraspiracle

LsRXR belongs to the nuclear hormone receptor family. The putative full length protein sequence contain a highly conserved DNA binding domain (DBD) and a well conserved ligand binding domain (LBD) in a typical context. Our phylogenetic analysis place LsRXR in a group of arthropod nuclear hormone receptors (see Figure 2). The conserved protein domain composition and the phylogenetic analysis strongly support that LsRXR is a homologue to the insect ultraspiracle (USP) nuclear receptor. Sequence analysis of the present *L. salmonis* RXR protein revealed a unique insertion of 10 Asp residues not found in any other similar sequences deposited in GenBank. These Asp residues clearly influence the structure of the predicted protein, but the significance of this is unknown. In insects and other arthropods, an important function for USP is through interaction with EcR to mediate ecdysteroid signaling. In a draft assembly of the salmon louse genome, we identified a putative EcR. Another partner for the *Drosophila* USP is DHR38, the homologue of the vertebrate NR4A [35-37]. A putative *L. salmonis* DHR38 homologue was also identified in the draft genome.

We identified five different LsRXR transcripts in our RACE experiments, which were identical in sequence but truncated resulting in transcripts differing in length (see Figure 1). The cDNA blot confirmed the presence of at least two distinct forms of LsRXR transcripts (see Additional file 1: Figure S1). The two major bands (i.e. about 1500 bp and 800 bp) identified in the cDNA blot corresponds to LsRXRa/b/c containing both DBD and LBD and a short form containing an incomplete or no DBD (LsRXRd/e). Our Southern Blot indicated only one copy of the *LsRXR* gene, and scrutinizing the draft *L. salmonis* genome assembly supports that LsRXR is a single copy gene. The *LsRXR* has two introns but the difference in transcript length was not related to alternative splicing. In other arthropods, RXR/USP can be present as different isoforms separated in time or space (i.e. transcribed in different cell types or at different developmental stages) e.g. [38-44]. In several species, multiple mRNAs have been detected for RXR/USP. Henrich, et al. [7] found two different *usp* transcripts (i.e. 2.7 kb and 1.2 kb) in *Drosophila* where the 2.7 kb transcript is corresponding to the full length *usp* transcript. Kapitskaya, et al. [45] found four different USP transcripts in the mosquito (*Aedes aegypti*). Guo, et al. [46] found a diversity of RXR-transcript in the ixodid tick (*Amblyoma americanum*). The function of most of these transcripts (of atypical length) is not known. Our real time quantitative PCR analysis of different developmental stages showed that the shortest transcript (i.e. LsRXR e) was equally abundant as the large forms (see Figure 4). It is obvious that variants lacking the DBD are unable to bind to DNA and act as a transcription regulator in that way, but it is possible that these LsRXR variants may modulate ligand levels by binding ligands. Recently, Montagne, et al. [47] identified a DHR3 variant that lacked the DBD in *Drosophila* and they showed that this particular variant was responsible for interaction with S6K.

Of the included developmental stages, the highest level of LsRXR (all variants) transcription is seen in copepodids. There is a marked increase from naupli II to copepodids and an equal decrease for the remaining developmental stages. This transcriptional pattern is not reflected in expression of EcR which exhibit equal levels in naupli II and copepodids followed by a reduction in the remaining developmental stages.

### LsRXR is regulating oogonia maintenance and yolk protein production

By knocking-down LsRXR in reproducing female lice effects can be seen directly in the female itself but also indirectly by affecting production of maternally components for incorporation in the oocytes. In addition, it is possible that knock-down could affect ligand levels (i.e. ecdysteroids) incorporated into the oocytes. This means that a range of possible effects could be the consequence of knocking-down LsRXR in reproducing female lice.

We could not discover any macroscopic abnormality or increased mortality in the female lice receiving dsRNA from LsRXR during the experimental period. However, histological examination revealed some significant effects at the cellular level both in the ovary and in the oocytes present in the genital segment. In the most severe case the ovarium did not contain any normal oogonia at all (see Figure 6). The consequence of this would be no ovulating oogonia and a complete interruption of the egg production. However, most lice produced and extruded fertilized eggs and the reason for this may be that low levels of LsRXR were present during the production of the first egg batches. In *Drosophila*, EcR is required for normal oogenesis [48] and EcR and USP are expressed in nurse and follicle cells throughout oogenesis [48,49]. Furthermore, it has been shown in *Drosophila* that oogenesis in EcR mutants are deficient [48] resulting in abnormal egg chambers and loss of vitellogenic egg stages. The present study shows that USP is necessary for oogenesis as well.

The histological examination revealed limited egg yolk deposition in the maturing oocytes (see Figure 6). The microarray and subsequent Q-PCR analysis confirmed a large reduction in the mRNA levels of the three major salmon louse yolk proteins, i.e. LsVit1 and LsVit2 (see [17]) and LsYAP (see [16]). It is common that vitellogenins like the LsVit1 and LsVit2 have binding sites for steroid hormone receptors in the gene promoter (e.g. [25,50]) and the present study support that these two *L. salmonis* vitellogenins and LsYAP are under control of LsRXR. LsYAP is also a major yolk protein in the salmon louse. The strongly reduced levels of yolk proteins can be a major factor explaining the poor development and survival of eggs produced by female lice receiving dsRNA treatment. However, other factors are needed to explain the reduced number of eggs produced by the lice when LsRXR was knocked-down.

There are no detailed studies on regulation of Vg-transcription in any copepods so far but many other arthropods have been studied. Female reproduction is energetically expensive and access to amino acids and other building blocks is necessary for production and maturation of gametes. This means that nutritional signaling is crucial in addition to hormone regulation (i.e. E20 and JH). In *A. aegypti,* Vg-transcription is regulated through nutrients (i.e. blood meal derived amino acids) and E20. The nutrient based signaling is signaled through TOR whereas E20 mediated signals are transmitted through EcR/USP. In *A. aegypti* at least two GATA factors are involved in mediating the signal where one act as a repressor [26] and one as an activator [51]. Translation of the latter is regulated by the TOR signaling pathway following a blood meal [52].In the present study TOR and the S6 kinase are unregulated but one GATA-factor was up regulated pointing towards an inhibitory effect for this particular transcription factor on vitellogenin production. In locusts neuroparsin inhibits vitellogenesis [52]. In the present study a neuroparsin homologue is strongly up regulated (1351 times) in the LsRXR RNAi lice. Neuroparsin is a multifunctional neuro hormone which is known to inhibit the effects of juvenile hormone in locust and to induce an increase in hemolymph lipid and trehalose levels. Hence, it is possible that the strong induction of neuroparsin in the salmon louse could be involved in down regulating yolk protein synthesis. However, further studies are required to rule out the function of neuroparsin and other potential transcription regulators for vitellogenesis in the salmon louse.

Although the main impression is that Vg synthesis is under direct control of ecdysteroids (i.e. E20) in athropods exceptions are known. In the beetle *T. castanneum* JH induce Vg synthesis but not 20E [11]. In *T. castanneum* vitellogenesis is only indirectly regulated by 20E by regulation of ovary growth and oocyte maturation [10] and knock-down of EcR or USP resulted in disruption of egg development [53,54]. This is similar to cockroach where vitellogenesis is JH dependent and independent of E20 [11,55]. Furthermore, in honeybee down regulation of USP did not influence Vg expression [56] and in crayfish [53,57] vitellogenins were not classified as ecdysteroid responsive.

It is well known that a wide diversity of mRNAs are deposited into oocytes during maturation. Some of these may be translated in the oocytes or the mature eggs prior to fertilization but a significant proportion are activated and utilized after fertilization. In particular, the period between fertilization and the onset of zygotic transcription, maternal provided mRNA and/or proteins are essential for proper early development. Our microarray contains 67 strictly maternal transcripts identified in *Drosophila* [34]. Of these two are down regulated and three are up regulated as a result of knocking-down LsRXR (see Additinal file 4: Table S9). This altered regulation may result in early developmental abnormalities after fertilization prior to the onset of zygotic transcription. From the females that produced eggs that hatched, a range of different phenotypes were evident and it is very difficult to point to abnormalities that could be explained by changed levels of a single transcript. Most likely the deformation observed is a combination of altered levels of several different transcripts. In addition, the phenotypes of the developing larvae will also be influenced by altered levels of maternally provided proteins, other than the yolk proteins.

Oro, et al. [6] showed that USP has multiple functions throughout the life cycle of *Drosophila*. In adult female *Drosophila* USP function is crucial in both germline as well as somatic tissues. LsRXR is transcribed in the ovarium and transcripts are present in the lumen of the ovarium follicles indicating a function in ovarian maintenance comparable to *Drosophila*. USP-transcripts are also present in oocytes and mature eggs. The maternal derived USP mRNA in *Drosophila* is required for production of cuticle in late embryos [6]. Our RNAi results points to similar function as hatching larvae were strongly deformed indicating a dysfunction in the exoskeleton.

### LsRXR is a regulator of cuticle production

The heterodimeric nuclear receptor consisting of EcR and USP in insects was originally shown to be key players for molting [58,59]. Increase in E20 levels precedes molting and the increased hormone level is mediated through the EcR-USP complex by binding directly to promoter of target genes, which includes several early and late response genes. Molting is a complex process where the net result is a new cuticle that will allow the animal to grow. A large number of cuticle genes have been identified in different arthropods. A hallmark in the present study was a large number of putative cuticle proteins being up regulated after knocking-down of LsRXR. Egg producing adult female lice do not grow post molting maturation (see [18]) and increased levels of cuticle proteins is unexpected. Hence, this points to induction of cuticle protein production when steroid hormone levels decrease. This agrees with results from *Drosophila* studies using USP mutants under pupuration. Instead of forming a pupa, USP mutant deposits a supernumerary cuticle similar to larval cuticle rather than a pupal cuticle [60]. Another characteristic for these USP mutants is that the programmed cell death in larval midgut and the following adult midgut proliferation was disrupted. In our experiment many anti apoptosis genes were up regulated and apoptosis genes were down regulated in the LsRXR RNAi lice.

## Conclusions

The LsRXR has a critical function during reproduction in female salmon louse. The sequence analysis revealed unique structural features due to insertion of 10 Asp residues in the T-box area and the significance of these additional residues should be elucidated in the future. Five different LsRXR transcripts different in length and domain composition were identified. The two shortest transcripts had incomplete or lacked the entire DBD. The shortest form showed highest abundance during our Q-PCR assessment. Our phylogenetic assessment put LsRXR in a group with USP and similar receptors from insects and not with the malacostracans. To further characterize LsRXR we knocked down LsRXR transcript levels by RNAi in adult female lice. This resulted in strong effects on female reproduction giving a strong reduction or complete stop in egg production. For those lice that produced eggs that were fertilized, close to zero viable larvae were evident. Histological examination of some of these female lice revealed significant effects in the ovary and the maturing oocytes. In the most severe case no oogonia was present in the ovary which would result in no egg-production. For those lice that produced and laid eggs the vitellin crystals were absent or strongly irregular. Our microarray assessment showed that by knocking-down LsRXR resulted in large effects on transcription on a diversity of genes. A main impression is that reproduction and lipid metabolism is down-regulated whereas growth related processes are up regulated. We show that LsRXR is crucial for both oogonia production and for providing sufficient building blocks for the oocytes both in terms of mRNA and proteins. The significant effects of knocking down LsRXR clearly makes this a target for salmon louse control. Chemicals interacting with the function of LsRXR are likely to give similar strong effects that we observed in the RNAi experiment. In addition, it is possible that some of the downstream regulated genes could serve as potential targets for lice control.

## Methods

### Handling and sampling of fishes and parasites

*L. salmonis* were cultured on Atlantic salmon (*Salmo salar* (Linnaeus)) as described previously [61]. All experimental procedures were done in accordance with national legislation. Salmon lice sampled for Southern blot analyses and genome walking were stored on 70% ethanol until DNA extraction or utilized immediately upon sampling. Salmon lice sampled for microarray, and Q-PCR analyses were immediately placed on dry ice and stored at −80°C until RNA extraction. Samples (see below) for cDNA blotting were collected and stored on RNA later. Experiments were carried out in strict accordance with Norwegian legislation and the experiment was approved by the Norwegian Animal Research Authority (permits nr. 2010/245410 and 2009/186329).

### Sequencing of gDNA and full-length cDNA

Expressed Sequence Tags (ESTs) from *L. salmonis* were obtained as described previously [19] and are described in Eichner, et al. [18]. LsRXR encoding EST clones were selected and completely sequenced on both strands using primer walking. Additional 5′ sequence was obtained by 5′ RACE as described previously [19]. Based on the cDNA sequences, primer pairs for genomic PCRs covering the entire CDS and most of the UTRs were constructed and PCR products were cloned and completely sequenced.

### Southern blot analysis

The number of LsRXR gene copies in the salmon louse genome was assessed using southern blot analysis as previously described [31] using seven restriction enzymes (ClaI, KpnI, PstI, SacI, XbaI, XhoI and XmnI) and loading five μg DNA well-1. Probes were generated against the 3′ end of the CDS (Figure 1). Randomly P32 labeled probes were synthesized using Amersham Rediprime II DNA Labeling Kit (GE Healthcare) in accordance with the manufacturer’s instructions. To generate a 349 bp long PCR product for the labeling procedure primers listed in Table 5 were used.

**Table 5:** **Primers used for dsRNA template synthesis, for RNA probe synthesis for in situ hybridisation (ISH), southern probe synthesis and probe synthsis for cDNA blotting**

**Type of use**	**Sense strand**	**Antisense strand**
dsRNAFr2, ISH 3′, southern	#CGGAATTGGGATGTCTACGAGCCATCATA	#CTTCCTCTGACTCACTATAGAAGCATA*
dsRNAFr1, ISH 3′	#CATGAGTGGAGGGGGTTCTATTGGGGATAT*	#GCGATGAGTTCAACTAGGACACGATCGAAT
ISH 5′	#GCCTCAACTCCTTGTTGTTCCTGCTT	#TCACACAATCCCGATTTTCTCTGCA
cDNA blot	CATCGAGAGGATCATTGAGGCAGAA	CGACTTGGCTCATTCTCATGAACAGA

*These primers were used for both, dsRNA template synthesis and RNA probe synthesis for in situ measurement.

#primers which were produced with and without T7 (TAATACGACTCACTATAGGG).

### cDNA blotting

In order to validate the RACE results a cDNA blot [62] was selected as standard Northern blot with non-radioactive labelling proved difficult for this transcript. Samples were obtained from eggs (1 egg string), nauplia (I + II in a 1:1 proportion, obtained from 1 egg string each), planktonic copepodids (from 1 egg string pair), preadult male (12 lice), preadult female (8 lice), adult male (4 lice) and adult female lice (3 lice) and used as template for the cDNA blotting.

Total RNA was isolated by Tri Reagent (Sigma Aldrich) according to manufacturer’s recommendations. Homogenization of the sample was carried out using 1.4 mm zirconium oxide beads (Precellys 24) and a TissueLyser LT (Qiagen) for three times two minutes at 50 Hz. Samples (3 μg) were reverse transcribed using Affinity Script qPCR cDNA Synthesis Kit (Stratagene) according to protocol with affiliated oligo-dT primers in 20μl reactions. Resulting RNA was ethanol precipitated (0.3 M Sodium acetate, pH 5.2, 3 volumes 100% EtOH) over night at −20°C, spinned at 21000 g for 30 min at 4°C. The pellet was washed with 70% ice cold EtOH, dried and resuspended in 10 μl ddH_2_O.

Some modifications to the protocol of Jaakola et al. [62] were implemented. To avoid secondary structures of the single stranded cDNA which are likely to change properties while running the gel, samples were run on a denaturating 1% agarose gel (12.3 M Formaldehyde, 1 × MOPS buffer) for 4 hours at 50V. Markers used were MassRuler Low Range DNA Ladder (Thermo Scientific) and 1 kb DNA Ladder (New England Bioloabs). The entire sample volume of each sample was added GelRed (1:100 in 6× loading buffer) [63]. After taking photographs (GelLogic), the gel was washed in H_2_O and two times in 10 × SSC buffer for 15 min. The Gel was blotted on a Hybond-N^+^ membrane for about 20 hours. The probe was covalent linked to the membrane by UV crosslinking (Hoefer VVC500 UV Cross linker, 70000 μJ cm^−2^).

A 567 bp long probe was synthesized with primers listed in Table 5 according to PCR DIG Probe Synthesis Kit (Roche). Hybridization was carried out at 42°C in ULTRAhyb® Ultrasensitive Hybridization Buffer according to manufacturer’s instructions. Probe detection was done as described in DIG Northern Starter kit (Roche).

### Quantitative real time PCR (Q-PCR) analysis

Q-PCR was performed and analyzed as described below using the established salmon louse eEF1α standard gene assay [64] and the TaqMan assays (FAM) listed in Table 1.

Eight dilutions of RNA were used for standard curve, for other measurements two or in some cases three dilutions. Standard curves were run on all selected stages as well as for RNAi lice for each assay used. All RNA was diluted 10 times or more with ddH_2_O to dilute all inhibitors which could interact with the cDNA synthesis. cDNA synthesis was performed with Quanta cDNA synthesis kit according to the manufacturer’s instructions and diluted four times before storage at −20°C until use.

Q-PCR was conducted in duplicates for each dilution. It was carried out independently but simultaneously for eEF1a and the test genes using same cDNA and master mix (TaqMan® Fast Universal PCR Master Mix; Applied Biosystems). Thermal cycling and quantification was done on the Applied Biosystems 7900 Fast Real-Time PCR System under standard conditions (initiation: 50°C 2 min, holding: 95°C 10min, 40 cycles of 95°C 15 seconds then 60°C 1 min).

Adult female lice from the knock down experiment were treated as individual samples; lice from all other stages were pooled in samples containing two to 10 individuals. LsRXR (assay1) was measured in all lice from the experiment, while LsRXR (assay2) was only measured in selected samples (three control lice, three RNAi lice and all different stages).

Relative quantification analysis was calculated as: eff^-ΔCT^, with ΔCT = CT_target_-CT_standard_, where eff = efficiency, CT = cycles at threshold fluorescent level. The efficiency was calculated by the slope of standard curves (Ct vs. log quantity) with eff = 10^(−1/slope)^. In case of several dilution curves for the same lice stage as done for lice in knock down experiment, a mean of efficiency was taken for calculation. The efficiencies for all assays used in this study were comparable.

Q-PCR analysis has been performed with 66 samples from the RNAi experiment; 18 from the control group, 29 from the group injected with fragment 1 and 19 from group injected with fragment 2. Q-PCR was also performed on lice from various developmental stages: Nauplia II, free swimming copepodids, chalimus I, chalimus II, preadult I females, preadult I males, preadult II females, preadult II males, and adult males.

### LsRXR transcript localization

The localization of LsRXR mRNA in adult female salmon lice was determined by *in situ* hybridization as described previously [20]. One 681bp and one 508bp RNA probe was synthesized on cDNA using the primers listed in Table 5. Hybridizations were performed with antisense probes to localize transcripts and sense probes as a negative control. Labeled probes were visualized using anti-digoxigenin (DIG) alkaline phosphatase fragment antigen binding (FAB) fragment (Roche) and a chromogenic substrate containing levamisol (Sigma–Aldrich), nitroblue tetrazolium (NTB; Roche) and 5-bromo-4-chloro-3-indolyl phosphate (BCIP; Roche).

### LsRXR knock down using RNAi

Knock down of LsRXR was done as previous described [16] with the exception that two dsRNA fragments covering different parts of the LsRXR transcripts were used separately to confirm knock down specificity. Primers used to generate templates for dsRNAs are listed in Table 5. DsRNA was generated using the Silencer® siRNA Construction Kit (Ambion).

The concentrations of these products were measured by spectrophotometry (Nanodrop ND-1000, Thermo Fisher Scientific). A final dsRNA concentration of 0.6 μg/μl was used. Fifty females in separate groups were injected with each of the two LsRXR dsRNA Fragments (Fragments 1 and 2, see Figure 1) or with control dsRNA (cod trypsin gene see [16]) and handled as previously described [16]. Ten lice were put on each fish (five injected females and five uninjected males). Groups of six fish were put into tanks and kept as previously described [61] at nine degree water temperature. RNA-injected females were recovered after appearance of the second pair of egg strings. Egg strings were removed from the lice with forceps, photographed for length measurement and placed in individual hatching containers. The hatching egg strings were inspected daily. Lice were after photographing sacrificed by directly freezing on dry ice and were kept on −80 degree until analysis. Five lice from the group with already hatched egg-strings (see below) were put back on fish again to assess the hatching success of this group of lice. Five RNAi lice were fixed in phosphate buffered 4% paraformaldehyde (pH 7.4), for histological analysis.

### Haematoxylin-erythrosin-saffron stained sections

Adult female lice were fixed in phosphate buffered 4% paraformaldehyde (pH 7.4) overnight and then dehydrated in Ethanol, cleared in xylene and embedded in paraffin wax. Sections, 3.0 μm thick (Leica Jung Biocut 2035), were incubated for 30 min at 65°C, dewaxed in xylene, rehydrated through a graded ethanol series and washed in distilled water. Sections were stained with Haematoxylin-erythrosin-saffron (HES). (Haematoxylin: 2.5 minutes, water: 4 minutes, 1% Erythrosin, pH: 6.5: 1.5 minutes, water, 96% ethanol and 2 × 100% ethanol for 1 minute each, alcoholic saffron: 20 seconds, 2 × 100% ethanol:1 minute each, 2 × xylene: 5 minutes each).

### Microarray analysis

#### General

Microarray analysis was performed on a total of 34 adult female *L. salmonis*, representing the three different groups: control group injected with double stranded control RNA, lice injected with fragment 1 and lice injected with fragment 2. Lice were chosen according to the time point of hatching of their larvae (see Table 6). 12 lice were chosen from the control group, 15 from the group injected with fragment 1 and seven from the group injected with fragment 2, as fragment 1 was used as the main group, and fragment 2 was used to confirm results from fragment 1.

**Table 6:** **Overview of treatments and classification of lice used for microarray hybridization**

**Louse no**	**# lice**	**Fragment injected**	**# days to hatch**	**Subgroup**
A687, A713, A720	3	Control	5	Control_5
A692, A689, A706	3	Control	6	Control_6
A700, A718, A724	3	Control	7	Control_7
A701, A710, A711	3	Control	8	Control_8
A764, A773, A785, A789, A791	5	Fragment 1	1-2	Fr1a: “Early hatching”
A767, A777, A783, A786, A788	5	Fragment 1	6-9	Fr1b: “Late hatching”
A765, A770, A779, A781, A784	5	Fragment 1	No	Fr1c: “No hatching”
A747, A755, A751, A734	4	Fragment 2	6-9	Fr2b:“Late hatching”
A749, A761, A738	3	Fragment 2	No	Fr2c: “No hatching”

### Microarray design and production

#### cDNA library construction and EST sequencing

13 different libraries have been constructed. For an overview of compounds see Table 3. Total RNA was extracted by Trizol (Sigma) or by RNeasy Mini Kit (Qiagen). PolyA RNA was enriched by Poly(A) Pure™ or Poly(A) Purist™ (Ambion) respectively. For description of construction of two cDNA libraries (FB and ME) which were constructed in lambda ZAP and two cDNA libraries (PU and HA) which were constructed directly in the pBluscript SK+ vector as well as the subtracted cDNA library (SB) see [18]. The three normalized libraries were made using Smart kit (Evrogen) in accordance with supplied protocols. All clones were blue-white screened and clones were picked randomly for plasmid purification. Bacteria were grown overnight in 96 well dishes (Millipore) and plasmids were purified according to the recommendations from the manufacturer (Millipore). Clones were sequenced using vector primers T3, m13f or m13r and BigDye chemistry (Applied Biosystems). All ESTs have been submitted to GenBank (see [65]).

#### Sequence processing

The reads were base-called using Phred [phred], which resulted in 35577 sequences after quality filtering (GenBank accession HO666803-HO702379). The reads were masked for E. coli contamination and vector sequences using UniVec by RBR [rbr] and SeqClean, and reads shorter than 90bp were removed. The remaining 31055 reads were processed with the TGICL [tgicl] pipeline, which uses BLAST [blast] to cluster reads and CAP3 [cap3] for assembling the clusters. This resulted in 21877 reads being clustered and assembled into 4150 contigs and 11141 singletons.

#### Annotation

The contigs and singletons were aligned against UniRef 90 [u90] using BLASTX with an E-value threshold of 10^−7^ and a seed word size (−W) of 4, assigning a putative protein to 3977 contigs and 5763 singletons. This resulted in annotation of 5253 of the probes. The BLASTX results were also used with GOA to assign GO annotations to 1517 of the probes.

#### Array design

A 44k Agilent oligo microarray was designed using the Agilent eArray program according to their instructions. The array was designed to use 60-mer oligos with a 3′ bias, with five oligos for each contig, and three oligos for each singleton. In addition, a set of probes were added for sequences of particular interest, for a total of 41130 probes. Besides the custom oligos, spikes and controls according to the Agilent recommendations were used. Additional linkers to attach the 60mer oligos have been chosen.

### RNA isolation for microarray hybridization and real time PCR

RNA was isolated from individual animals using the RNA easy Mini kit (Qiagen) according to the manufactures recommendations. Homogenization of lice was done with a handheld rotor stator tissue homogenizer (Ultrathorax). The isolated RNA was DNAse treated by TURBO DNA-free™ (Ambion) according to the supplied protocol and 0.5μl Superase (Ambion) was added. The RNA samples were frozen at −80°C until usage. One aliquot was used for RNA integrity and quantity analysis using the Agilent 2100 Bioanalyzer and NanoDrop Spectrophotometer (OD 260/280 and 260/230 ratios). Another aliquot was used for cDNA synthesis and labeling (Quick Amp Labeling kit, one color, Agilent).

### Microarray hybridization

RNA was amplified, labeled and hybridized after the instructions of Agilent for one-color Microarray-Based Gene Expression analysis with the Quick Amp Labeling kit. 700ng total RNA was used as input for the reaction. Labeling efficiency and quantity of labeled cDNA was determined using the NanoDrop Spectrophotometer. Slides were scanned directly using an Agilent scanner at a resolution of five μm with settings outlined in the protocol. Data were extracted using Agilent feature Extraction software 9.5.3 as described in instructions. Analysis of data has been performed in J-Express (version 2009) software package [66]. To identify genes that are significantly differentially expressed between groups significance analysis of microarrays (SAM) [67] has been used on log2 transformed data. Only genes with a q-value less than 0.05 were chosen. All oligos belonging to the same cluster/singleton were inspected and only clusters/singletons which comprised all or in case of very many oligos at least 70% of all oligos having a similar q-value were chosen for further analysis. Average of the expression of oligos from each differently expressed cluster/singleton was calculated for further analysis.

We provide MIAME-compliant description of the microarray study, available in the array express database (http://www.ebi.ac.uk/arrayexpress/), accession number: A-MEXP-2224.

### Phylogenetic analyses

The phylogenetic analyses were conducted as described elsewhere [17]. Prior to phylogenetic analysis the sequence set was aligned using ClustalW. The alignment was manually inspected to ensure that homologous regions were properly aligned. The alignment was used for phylogenetic inference using protml [68] and MrBayes [69]. In the protml analysis the Jones-Taylor-Thornton model of amino acid change was used together with a gamma rates variation with an alpha = 0.421656 (REF). MrBayes was run using a GTR + I + G model with four chains and it was run for 250000 generations. [EMBL: ACP19739] *Calanus finmarchicus*, [GenBank: ABF74729] *Daphnia magna*, [EMBL: CAL25729] *Tribolium castaneum*, [NCBI Reference Sequence: XP_002424949] *Pediculus humanus corporis*, [EMBL: CAB75361] *Tenebrio molitor*, [GenBank: BAF85823] *Liocheles australasiae*, [NCBI Reference Sequence: XP_002435070] *Ixodes scapularis*, [EMBL: BAD99298] *Leptinotarsa decemlineata*, [GenBank: AAU12572.1] *Reishia clavigera*, [Swiss-Prot: Q8T5C6] *Biomphalaria glabrata*, [Swiss-Prot: Q5I7G2] *Lymnaea stagnalis*, NCBI Reference Sequence: XP_001370409] *Monodelphis domestica*, [NCBI Reference Sequence: NP_008848] *Homo sapiens*, [2208436B] *Homo sapiens* (HNF4), [NCBI Reference Sequence: NP_001244539] *Macaca mulatta*, [GenBank: BAF75376] *Marsupenaeus japonicas*, [NCBI Reference Sequence: NP_990625] *Gallus gallus*, [NCBI Reference Sequence: NP_001011634] *Apis mellifera*, [EMBL: ACO44669] *Crangon crangon*, [EMBL: AAC15589] *Amblyomma americanum*, [NCBI Reference Sequence: NP_001155023] *Danio rerio*, [NCBI Reference Sequence: NP_476781] *Drosophila melanogaster*, [NCBI Reference Sequence: NP_001097126] *D. melanogaster* (HNF4), [EMBL: AAG24886] *Aedes aegypti* (USBa), [EMBL: AAG24887] *A. aegypti* (USBb), [EMBL: AAC72724] *A. aegypti* (HNF4), [NCBI Reference Sequence: XP_001663736] *A. aegypti* (ERR), [NCBI Reference Sequence: NP_724456] *D. melanogaster* (EcR), [Swiss-Prot: P49880] *A. aegypti* (EcR), [GenBank: BAF49029] *D. magna* (EcR-A1), [EMBL: AAC33432] *Celuca pugilator* (EcR), [EMBL: ACO92359] *Neomysis integer* (EcR), *Lepeophtheirus salmonis* (present study).

## Additional files

Additional file 1: Figure S1. cDNA blot showing LsRXR transcripts in a set of developmental stages. Several distinct bands were detected both at normal exsposure time of the blot **(A)** or in the over-exsposed blot **(B)**. In both A and B distinct bands at 800 and 1500 bp were evident. In the over exposed blot there is also a weak band at about 1100 bp in adult female (Af). Nau I + II = nauplius I and II, Cop free = freeliving copepodids, Pad m = preadult males, Pad f = preadult female, A m = adult male, A f = adult female. Additional file 2: Figure S2. Lice investigated in the microarray study and their egg-strings shown in full length (last column) and higher magnifications (middle column). Additional file 3: Figure S3. Illustration of sub-group classification of the adult female lice. The maturity of oocytes in the genital segment can be distinguished by investigating the time point when the eggs hatched. After extruding of egg strings new oocytes are maturing inside the genital segment. Hence, immediate hatching indicates very mature oocytes while late hatching indicates less mature oocytes in the genital segment. In the upper panel the phenotype of the mother louse is shown, and the lice with most mature oocytes are at the left side. In the lower panel the phenotype of egg strings is shown and the most mature egg strings (strongest pigmented) are at the left (i.e. one to two days to hatching). On the timescale (# of days to hatching for the eggs) the time point to hatching for the eggs from the lice coming from different sub groups is indicated (Co5 to Co8, “hatching before”, “early hatching”, “late hatching” and “no hatching”). Additional file 4: Table S1-Table S9. Expression of LsRXR in the different groups measured by qPCR and on microarray (mean values of each group). Additional file 5: Figure S4. Verification of the gene expression measured by microarray with Q-PCR. Six different genes found to be regulated in LsRXR RNAi by the microarray were selected and quantified by real time PCR. Gene expression was measured in three control lice (Control) and three LsRXR knock-down lice (Fr1) **(A)** as well as in different other lice developmental stages **(B)**. The genes are Vitellogenin1 (LsVit1) (on average 9.1 times down regulated on array), Vitellogenin2 (LsVit2) (on average 4.0 times down regulated on array), LsYAP (on average 1.5E + 26 times down regulated on array), two cuticle genes (CL333Contig1 (CL333) (on average 3.05E + 04 times up regulated on array) and Cl3250Contig1 (CL3500) (on average 1.14E + 06 times up regulated on array) and neuroparsin (CL3400) (on average 1348 times up regulated on array). Note the different scales in A and B figure of each gene. 0.01 was selected as scale for all eggyolk proteins. The vitellogenin genes are strongly down regulated, but still higher expressed than in other stages, while CL3250 and CL3400 are very strong up regulated, much higher than in any other stages. CL333 is strongly up regulated in knock-down lice, but this gene is very high expressed in younger stages. Additional file 6: Figure S5. Clusters/singletons which belong to the groups of up- or down regulated genes are shown with respect to libraries they originate from. Only clusters are shown, which are composed of EST from one of the following developmental stages: egg, nauplia, copepodid, chalimus, preadult, immature adult female, adult female and adult male.
