# Supplementary figures and images for: Characterization of a novel RXR receptor in the salmon louse (Lepeophtheirus salmonis, Copepoda) regulating growth and female reproduction
Source: BMC Genomics. 2015 Feb 14;16(1):81. doi: 10.1186/s12864-015-1277-y (PMC4333900; doi:10.1186/s12864-015-1277-y)

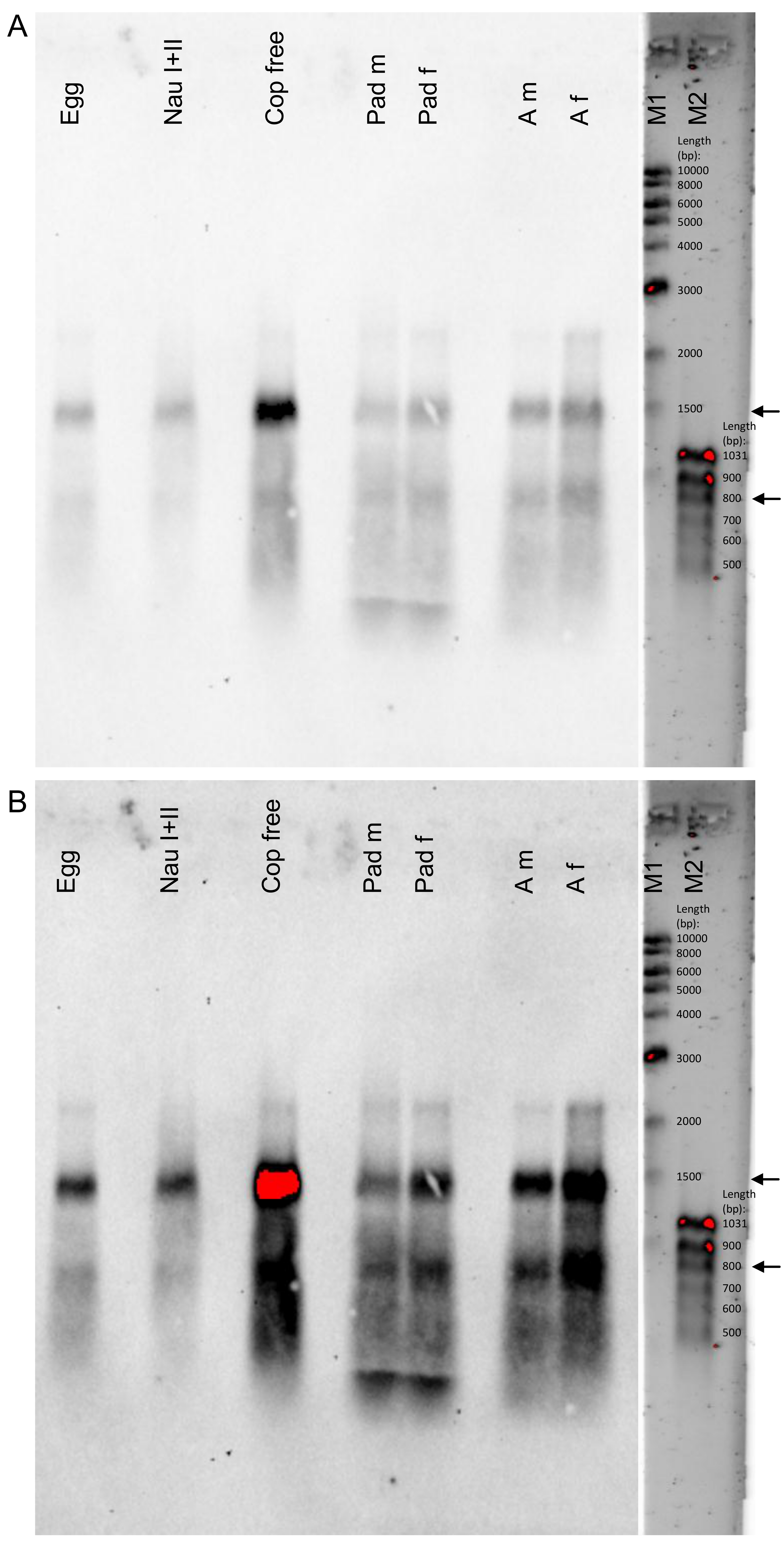

Supplement: Additional file 1: Figure S1. — cDNA blot showing LsRXR transcripts in a set of developmental stages. Several distinct bands were detected both at normal exsposure time of the blot (A) or in the over-exsposed blot (B). In both A and B distinct bands at 800 and 1500 bp were evident. In the over exposed blot there is also a weak band at about 1100 bp in adult female (Af). Nau I + II = nauplius I and II, Cop free = freeliving copepodids, Pad m = preadult males, Pad f = preadult female, A m = adult male, A f = adult female. [file 12864_2015_1277_MOESM1_ESM.tiff]

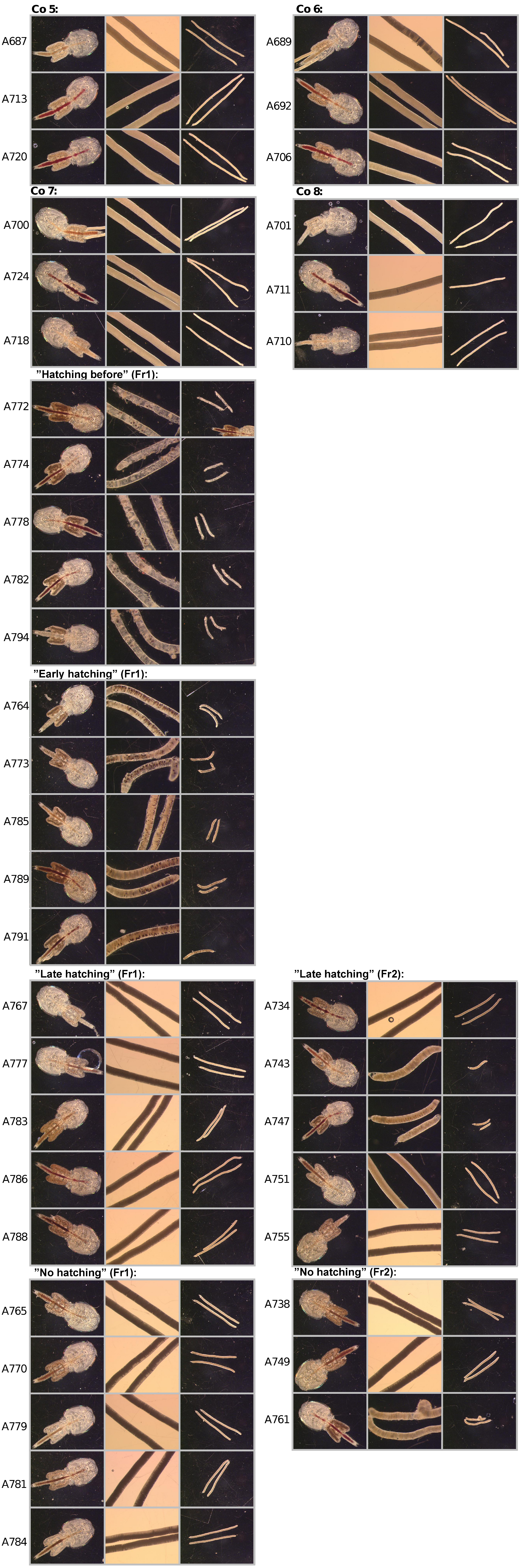

Supplement: Additional file 2: Figure S2. — Lice investigated in the microarray study and their egg-strings shown in full length (last column) and higher magnifications (middle column). [file 12864_2015_1277_MOESM2_ESM.tiff]

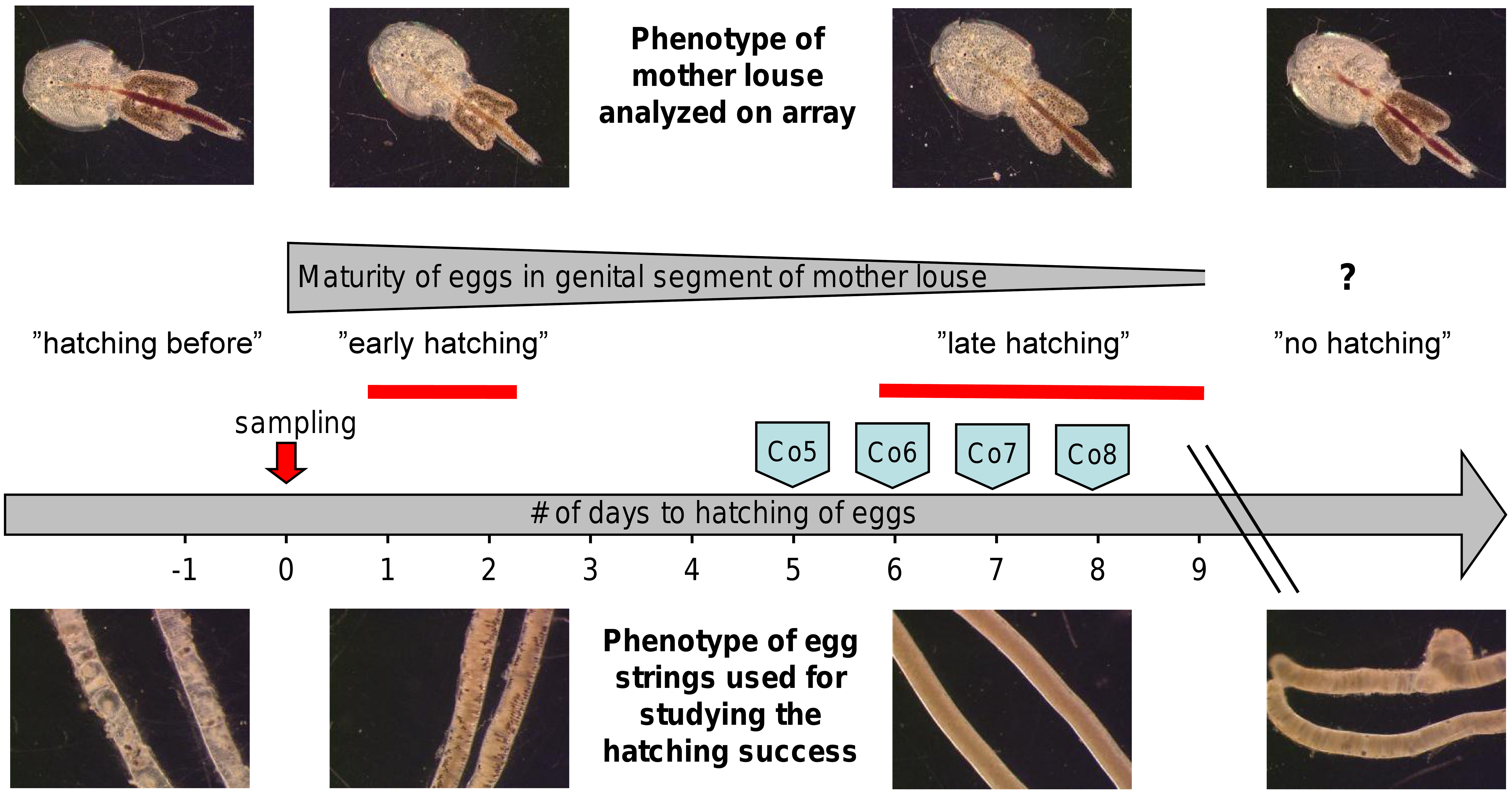

Supplement: Additional file 3: Figure S3. — Illustration of sub-group classification of the adult female lice. The maturity of oocytes in the genital segment can be distinguished by investigating the time point when the eggs hatched. After extruding of egg strings new oocytes are maturing inside the genital segment. Hence, immediate hatching indicates very mature oocytes while late hatching indicates less mature oocytes in the genital segment. In the upper panel the phenotype of the mother louse is shown, and the lice with most mature oocytes are at the left side. In the lower panel the phenotype of egg strings is shown and the most mature egg strings (strongest pigmented) are at the left (i.e. one to two days to hatching). On the timescale (# of days to hatching for the eggs) the time point to hatching for the eggs from the lice coming from different sub groups is indicated (Co5 to Co8, “hatching before”, “early hatching”, “late hatching” and “no hatching”). [file 12864_2015_1277_MOESM3_ESM.tiff]

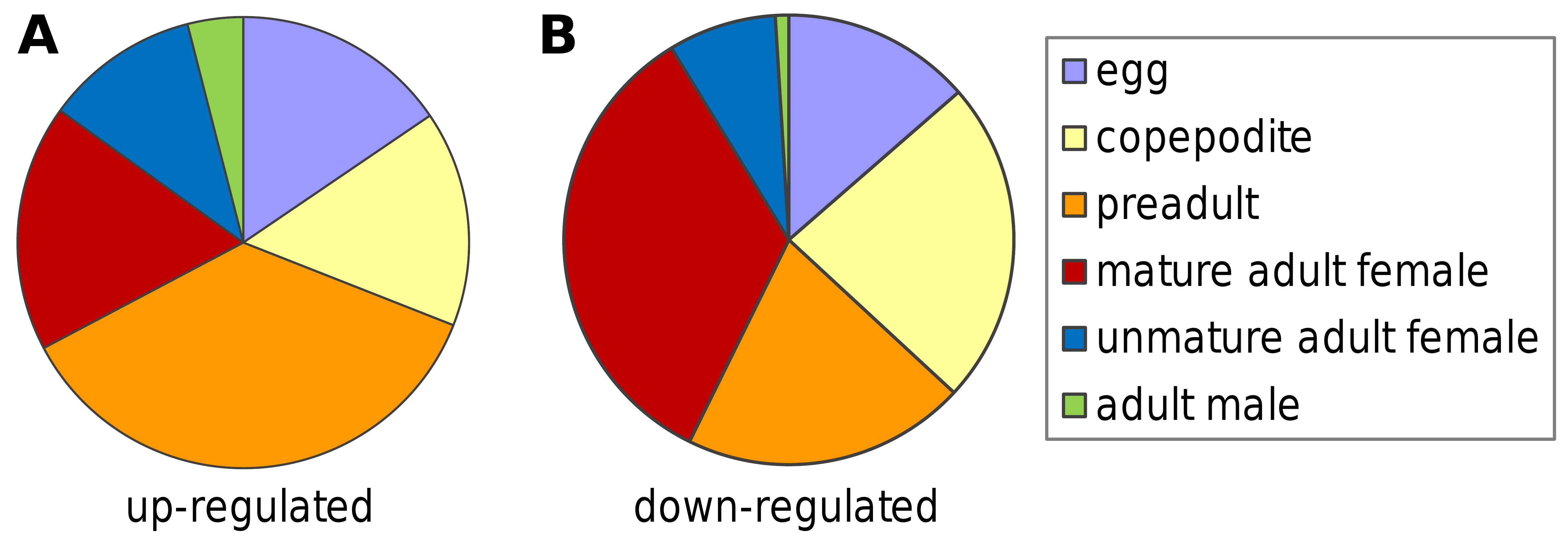

Supplement: Additional file 5: Figure S4. — Verification of the gene expression measured by microarray with Q-PCR. Six different genes found to be regulated in LsRXR RNAi by the microarray were selected and quantified by real time PCR. Gene expression was measured in three control lice (Control) and three LsRXR knock-down lice (Fr1) (A) as well as in different other lice developmental stages (B). The genes are Vitellogenin1 (LsVit1) (on average 9.1 times down regulated on array), Vitellogenin2 (LsVit2) (on average 4.0 times down regulated on array), LsYAP (on average 1.5E + 26 times down regulated on array), two cuticle genes (CL333Contig1 (CL333) (on average 3.05E + 04 times up regulated on array) and Cl3250Contig1 (CL3500) (on average 1.14E + 06 times up regulated on array) and neuroparsin (CL3400) (on average 1348 times up regulated on array). Note the different scales in A and B figure of each gene. 0.01 was selected as scale for all eggyolk proteins. The vitellogenin genes are strongly down regulated, but still higher expressed than in other stages, while CL3250 and CL3400 are very strong up regulated, much higher than in any other stages. CL333 is strongly up regulated in knock-down lice, but this gene is very high expressed in younger stages. [file 12864_2015_1277_MOESM5_ESM.tiff]

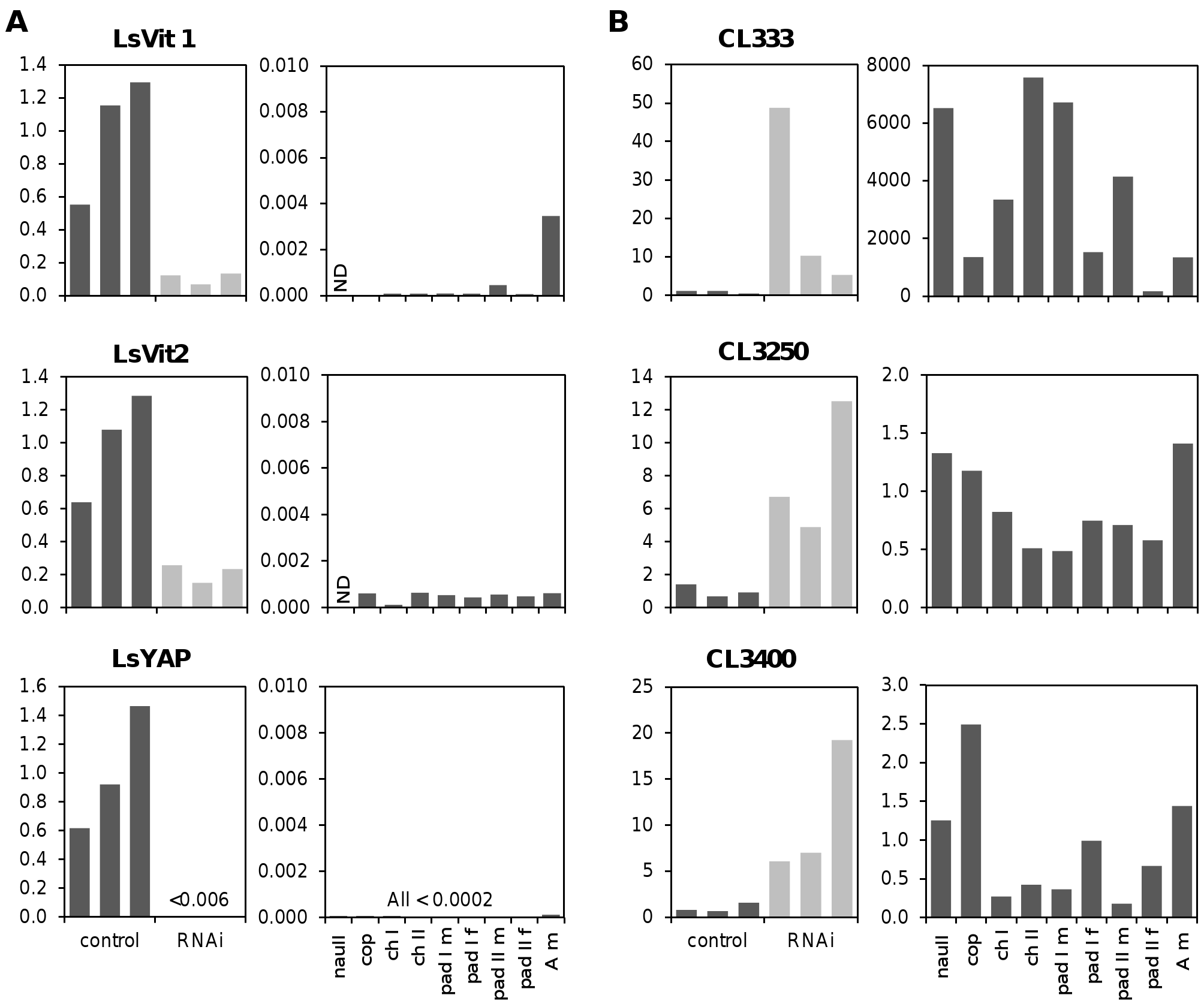

Supplement: Additional file 6: Figure S5. — Clusters/singletons which belong to the groups of up- or down regulated genes are shown with respect to libraries they originate from. Only clusters are shown, which are composed of EST from one of the following developmental stages: egg, nauplia, copepodid, chalimus, preadult, immature adult female, adult female and adult male. [file 12864_2015_1277_MOESM6_ESM.tiff]
