# Supplementary material for: Characterization of a novel RXR receptor in the salmon louse (Lepeophtheirus salmonis, Copepoda) regulating growth and female reproduction
Source: BMC Genomics. 2015 Feb 14;16(1):81. doi: 10.1186/s12864-015-1277-y (PMC4333900; doi:10.1186/s12864-015-1277-y)
Supplement: Additional file 4: Table S1-Table S9. — Expression of LsRXR in the different groups measured by qPCR and on microarray (mean values of each group). [file 12864_2015_1277_MOESM4_ESM.doc]

# Supplementary table 1. Expression of LsRXR in the different groups measured by qPCR and on microarray (mean values of each group)

| **Group mean of:** | **qPCR** | **Microarray (log2)** |
| --- | --- | --- |
| Control | 1, STDV: 0.28 | 1 |
| Fragment 1 | 0.35, STDV: 0.13 | 0.46, STDV: 0.03 |
| Fragment 2 | 0.44, STDV: 0.13 | 0.53, STDV: 0.04 |
| Hatching prior to harvesting | 0.44, STDV: 0.11 | - |
| Early hatching | 0.45, STDV: 0.18 | 0,54, STDV: 0.05 |
| Late hatching | 0.36, STDV: 0.11 | 0.46, STDV: 0.02 |
| No hatching | 0.33, STDV: 0.15 | 0.41, STDV: 0.03 |

# Supplementary table 2. All up-regulated clusters/singletons sorted after strength of regulation

| **no** | **Cluster,singelt.** | **composed of EST (name)** | **Hit UniRef/ GenBank** | **Library** | **Categorization** | **times up-regu-lated** |
| --- | --- | --- | --- | --- | --- | --- |
| 1 | CL2825 Contig1 | zslaa0_011456.z1.scf; LNO1557-2007-02-07.ab1 | Neurotransmitter gated ion channel *Aedes aegypti* (Yellowfever mosquito) | LNO | signal transduction | 1.35E+09 |
| 2 | CL3250 Contig1 | NLG1750-2007-08-23.ab1; NLG648-2007-06-13.ab1 | cuticular protein analogous to peritrophins 3-A2 [*Tribolium castaneum*] | NLG | chitin metabolic process | 1.14E+06 |
| 3 | CL333 Contig1 | HA539-2005-02-14 .ab1; zslaa0_011517.z1.scf; zslaa0_018209.z1.scf; zslaa0_011312.z1.scf; LF178-2007-01-09 .ab1; L1T1355-2007-01-23 .ab1; L1T1688-2007-01-12 .ab1; L1T1646-2007-01-12 .ab1; L1T_1125-2007-01-15 .ab1; LPA103-2007-05-15 .ab1; LF732-2007-03-16 .ab1; PU137-2004-09-20.ab1 | Cuticular protein *Tachypleus tridentatus* (Japanese horseshoe crab) | L1T, PU, LF, LPA, HA, LNO | cuticula | 3.05E+04 |
| 4 | CL1409 Contig1 | PU436-2004-09-30 .ab1; L1T1674-2007-01-12 .ab1; PU479-2004-09-30.ab1 | Cuticular protein *Tachypleus tridentatus* (Japanese horseshoe crab) | L1T, PU | cuticula | 28904 |
| 5 | zslaa0_005368 | zslaa0_005368.z1.scf | no hit | LNO | no hit | 4165 |
| 6 | L1T1708 | L1T1708-2007-01-24.ab1 | no hit | L1T | no hit | 2046 |
| 7 | CL763 Contig1 | zslaa0_005623.z1.scf; zslaa0_010070.z1.scf; zslaa0_002160.z1.scf; zslaa0_014665.z1.scf; L1T_1259-2007-01-15 .ab1; L1T406-2006-12-19 .ab1; LNO1648-2007-02-15.ab1 | CG13935PA *Drosophila melanogaster* (Fruit fly) | L1T, LNO | cuticula | 1546 |
| 8 | CL3400 Contig1 | HA239-2004-08-06.ab1; HA232-2004-08-06.ab1 | Neuroparsin 2 precursor *Schistocerca gregaria* (Desert locust) | HA | signal transduction | 1348 |
| 9 | CL804 Contig1 | zslaa0_008950.z1.scf; zslaa0_018255.z1.scf; zslaa0_010512.z1.scf; LNC771-2007-12-20 .ab1; zslaa0_012646.z1.scf; zslaa0_011270.z1.scf; LNO1605-2007-02-15.ab1 | glutathione peroxidase 7 [Ciona intestinalis] | LNO, LNC | stress response | 527.5 |
| 10 | zslaa0_005411 | zslaa0_005411.z1.scf | Collagen alpha1(VI) chain precursor *Gallus gallus* (Chicken) | LNO | cuticula | 254.6 |
| 11 | CL319 Contig1 | zslaa0_005206.z1.scf; LF1964-2007-05-09 .ab1; LF636-2007-01-11 .ab1; zslaa0_016853.z1.scf; HA821-2005-02-25 .ab1; LF1851-2007-05-08 .ab1; LF152-2007-01-09 .ab1; zslaa0_014022.z1.scf; LNC3114-2008-12-22 .ab1; LF968-2007-03-16 .ab1; LNO2715-2007-03-06.ab1 | similar to Collagen alpha1(II) chain precursor partial *Apis mellifera* | LF, HA, LNO, LNC | extracellular matrix | 207.0 |
| 12 | zslaa0_008083 | zslaa0_008083.z1.scf | no hit | LNO | no hit | 169.4 |
| 13 | CL224 Contig3 | zslaa0_006938.z1.scf; zslaa0_002804.z1.scf; HAV-FN-FN2270-2001-11-30.ab1; PU413-2004-09-30.ab1; LNO2166-2007-03-06.ab1; LPA135-2007-05-15.ab1; L1T1387-2007-01-23.ab1 | Cuticular protein *Tachypleus tridentatus* (Japanese horseshoe crab) | ME, L1T, PU, LPA, LNO | cuticula | 154.7 |
| 14 | FN4963 | FN4963-2003-03-12.ab1 | Cuticle protein 7 [*Lepeophtheirus salmonis*] | ME | cuticula | 138.2 |
| 15 | LPU305 | LPU305-2007-08-29.ab1 | CG6669PA *Drosophila melanogaster* (Fruit fly) | LPU | cell adhesion | 122.4 |
| 16 | CL224 Contig1 | L1T176-2006-12-01.ab1; LF1719-2007-05-08.ab1 | Cuticular protein *Tachypleus tridentatus* (Japanese horseshoe crab) | L1T, LF | cuticula | 100.9 |
| 17 | CL224 Contig4 | zslaa0_016354.z1.scf; CC193-2003-03-13.ab1 | Cuticular protein *Tachypleus tridentatus* (Japanese horseshoe crab) | CC, LNO | cuticula | 73.5 |
| 18 | FN436 | 337_FN436_SF1_T7.ab1 | Cuticle protein *Bombyx mori* (Silk moth) | SB | cuticula | 69.0 |
| 19 | LF1985 | LF1985-2007-05-09.ab1 | putative SPT transcription factor family member [*Lepeophtheirus salmonis*] | LF | transcription factor | 63.4 |
| 20 | CL3800 Contig1 | HA791-2005-02-25 .ab1; FN4620-2003-03-06.ab1 | FK506binding protein 2 precursor *Kluyveromyces lactis* (Yeast) (Candida sphaerica) | ME, HA | protein folding | 61.1 |
| 21 | LPU1175 | LPU1175-2007-08-29.ab1 | no hit | LPU | no hit | 50.6 |
| 22 | CL170 Contig1 | zslaa0_003787.z1.scf; zslaa0_000492.z1.scf; zslaa0_003324.z1.scf; zslaa0_003376.z1.scf; zslaa0_004571.z1.scf; zslaa0_013536.z1.scf; zslaa0_008644.z1.scf; zslaa0_014462.z1.scf; zslaa0_013443.z1.scf; zslaa0_005351.z1.scf; zslaa0_012102.z1.scf; zslaa0_007255.z1.scf; LNO421-2007-02-08 .ab1; LNO3552-2007-03-05 .ab1; LNC2307-2008-01-15.ab1 | similar to CG13313PA *Apis mellifera* | LNO, LNC | unknown function | 49.6 |
| 23 | CL224 Contig2 | LNO3887-2007-03-15.ab1; LF1533-2007-05-08.ab1 | Cuticular protein *Tachypleus tridentatus* (Japanese horseshoe crab) | LF, LNO | cuticula | 38.4 |
| 24 | CL925 Contig1 | zslaa0_008703.z1.scf; zslaa0_012360.z1.scf; zslaa0_015358.z1.scf; LNO1028-2007-02-01.ab1; LNO2064-2007-02-20.ab1; L1T173-2006-12-01.ab1 | Cuticular protein *Tachypleus tridentatus* (Japanese horseshoe crab) | L1T, LNO | cuticula | 38.3 |
| 25 | LNC2484 | LNC2484-2008-01-16.ab1 | no hit | LNC | no hit | 34.8 |
| 26 | CL2097 Contig1 | zslaa0_005418.z1.scf; LNO3033-2007-02-19 .ab1; NLG2482-2007-09-14.ab1 | similar to interferon gamma inducible protein 30 *Apis mellifera* | LNO, NLG | immune-regulatory genes | 29.5 |
| 27 | zslaa0_012006 | zslaa0_012006.z1.scf | similar to Collagen alpha1(XI) chain precursor *Tribolium castaneum* | LNO | extracellular matrix | 27.5 |
| 28 | CL937 Contig1 | zslaa0_003370.z1.scf; LF1039-2007-03-30 .ab1; LPU877-2007-08-29 .ab1; L1T971-2007-01-11 .ab1; L1T954-2007-01-11 .ab1; L1T1641-2007-01-12.ab1 | similar to Collagen alpha1(XI) chain precursor isoform 1 *Apis mellifera* | L1T, LF, LPU, LNO | extracellular matrix | 27.0 |
| 29 | FN3413 | FN3413-2002-03-08.ab1 | no hit | ME | no hit | 26.7 |
| 30 | zslaa0_010513 | zslaa0_010513.z1.scf | similar to CG13313PA *Apis mellifera* | LNO | unknown function | 26.1 |
| 31 | CL736 Contig1 | zslaa0_005581.z1.scf; zslaa0_007634.z1.scf; LPU1187-2007-08-29 .ab1; zslaa0_013674.z1.scf; LPU924-2007-08-29 .ab1; LNO3285-2007-02-15 .ab1; CC186-2003-03-13.ab1 | similar to leprecan 1 *Tribolium castaneum* | LPU, CC, LNO | negative regulation of cell proliferation | 24.3 |
| 32 | LNO126 | LNO126-2006-12-01.ab1 | Alkaline phosphatase tissue-nonspecific isozyme precursor [*Caligus clemensi*] | LNO | phosphatase | 24.0 |
| 33 | LPU1868 | LPU1868-2007-09-05.ab1 | no hit | LPU | no hit | 22.2 |
| 34 | zslaa0_018292 | zslaa0_018292.z1.scf | no hit | LNO | no hit | 21.7 |
| 35 | CL206 Contig1 | zslaa0_010817.z1.scf; zslaa0_015741.z1.scf; zslaa0_008848.z1.scf; zslaa0_016953.z1.scf; zslaa0_013464.z1.scf; zslaa0_012983.z1.scf; zslaa0_009639.z1.scf; zslaa0_007980.z1.scf; LF1514-2007-05-08 .ab1; LPU1780-2007-09-05 .ab1; L1T103-2006-12-01 .ab1; L1T560-2006-12-21 .ab1; LPU954-2007-08-29 .ab1; LF1942-2007-05-09 .ab1; L1T1492-2007-01-24.ab1 | similar to CG17052PA *Tribolium castaneum* | L1T, LF, LPU, LNO | chitin metabolic process | 19.5 |
| 36 | CL1248 Contig1 | zslaa0_007332.z1.scf; L1T192-2006-12-01.ab1; LNO3791-2007-02-20.ab1; LNO3661-2007-03-09.ab1; LNC1387-2007-12-21.ab1 | no hit | L1T, LNO, LNC | No hit | 18.8 |
| 37 | CL3122 Contig1 | PU718-2004-12-02.ab1; HA105-2004-07-28.ab1 | similar to Tryptophan 23dioxygenase (Tryptophan pyrrolase) (Protein vermilion) *Apis mellifera* | PU, HA | Tryptophan catabolism | 16.8 |
| 38 | FN1081 | 475_FN-1081-rerun_A11_081.ab1 | Leukocyte receptor cluster member 9. *Xenopus tropicalis* | FB | receptor | 16.6 |
| 39 | CL3562 Contig1 | zslaa0_016638.z1.scf; zslaa0_008286.z1.scf | no hit | LNO | no hit | 15.7 |
| 40 | CL456 Contig1 | zslaa0_015103.z1.scf; zslaa0_012539.z1.scf; zslaa0_007068.z1.scf; zslaa0_005536.z1.scf; LNO687-2007-02-06.ab1; LPA174-2007-05-15.ab1; LF1248-2007-04-23.ab1; zslaa0_000822.z1.scf | similar to CG4300PB isoform B *Tribolium castaneum* | LF, LPA, LNO | spermine biosynthetic process | 15.0 |
| 41 | CL2943 Contig1 | zslaa0_009221.z1.scf; zslaa1_002714.z1.scf | no hit | LNO | no hit | 14.6 |
| 42 | NLG845 | NLG845-2007-06-14.ab1 | no hit | NLG | No hit | 14.3 |
| 43 | LN01255 | LN01255-2007-02-08.ab1 | no hit | LNO | no hit | 14.3 |
| 44 | zslaa0_013255 | zslaa0_013255.z1.scf | no hit | LNO | no hit | 14.0 |
| 45 | CL1385 Contig1 | zslaa0_009259.z1.scf; zslaa0_017250.z1.scf; zslaa0_011675.z1.scf | Pyridoxalphosphate dependent enzyme family protein *Tetrahymena thermophila* SB210 | LNO | Amino acid biosynthesis | 13.9 |
| 46 | LNO1433 | LNO1433-2007-02-14.ab1 | no hit | LNO | no hit | 13.8 |
| 47 | LNC3095 | LNC3095-2008-01-16.ab1 | GABA alpha subunit *Lepeophtheirus salmonis* (salmon louse) | LNC | gamma-aminobutyric acid signaling pathway | 13.4 |
| 48 | CL1877 Contig1 | zslaa0_005816.z1.scf; zslaa0_007246.z1.scf | no hit | LNO | no hit | 13.3 |
| 49 | LNO1095 | LNO1095-2007-02-01.ab1 | Aquaporin 3 *Xenopus tropicalis* (Western clawed frog) (*Silurana tropicalis*) | LNO | transporter activity | 13.2 |
| 50 | LF1761 | LF1761-2007-05-08.ab1 | Pyridoxalphosphate dependent enzyme family protein *Tetrahymena thermophila* SB210 | LF | Amino acid biosynthesis | 13.1 |
| 51 | zslaa0_013828 | zslaa0_013828.z1.scf | no hit | LNO | no hit | 12.9 |
| 52 | zslaa0_003714 | zslaa0_003714.z1.scf | no hit | LNO | no hit | 12.9 |
| 53 | zslaa0_013180 | zslaa0_013180.z1.scf | Spermine synthase [*Lepeophtheirus salmonis*] | LNO | Spermine synthesis | 12.8 |
| 54 | LF1549 | LF1549-2007-05-08.ab1 | no hit | LF | no hit | 12.5 |
| 55 | CL720 Contig2 | zslaa0_012676.z1.scf; LNC3993-2009-03-05.ab1; LPU832-2007-08-29.ab1 | no hit | LPU, LNO, LNC | no hit | 12.2 |
| 56 | CL3636 Contig1 | zslaa0_013143.z1.scf; LPU915-2007-08-29.ab1 | papilin, isoform F [*Drosophila melanogaster*] | LPU, LNO | development | 12.0 |
| 57 | CL2354 Contig1 | LF144-2007-01-09 .ab1; LNO3829-2007-03-15 .ab1; LF1010-2007-03-30.ab1 | no hit | LF, LNO | no hit | 11.8 |
| 58 | zslaa0_018437 | zslaa0_018437.z1.scf | Integrin betaPS precursor *Drosophila melanogaster* (Fruit fly) | LNO | Embryonic and post embryonic development | 11.6 |
| 59 | CL2966 Contig1 | zslaa0_009630.z1.scf; zslaa1_010129.z1.scf | similar to CG13310PA *Apis mellifera* | LNO | unknown function | 11.6 |
| 60 | CL1836 Contig1 | zslaa0_001460.z1.scf; zslaa0_019242.z1.scf; zslaa0_012028.z1.scf | no hit | LNO | no hit | 11.2 |
| 61 | LNC472 | LNC472-2007-12-21.ab1 | Aldose 1-epimerase [*Caligus rogercresseyi*] | LNC | glycolysis/ gluconeogenesis | 10.1 |
| 62 | LNC1261 | LNC1261-2007-12-21.ab1 | proclotting enzyme-like isoform 1 [Acyrthosiphon pisum] | LNC | negative regulation of coagulation | 9.7 |
| 63 | CL1437 Contig1 | zslaa0_005849.z1.scf; LPU1116-2007-08-29 .ab1; LPU320-2007-08-29 .ab1; LPU807-2007-08-29.ab1 | Galactose mutarotase *Xenopus tropicalis* (Western clawed frog) (*Silurana tropicalis*) | LPU, LNO | glycolysis/ gluconeogenesis | 9.6 |
| 64 | LNO1878 | LNO1878-2007-02-16.ab1 | similar to Dermis expressed 1 *Strongylocentrotus purpuratus* (Twist-related protein 2) | LNO | development | 9.5 |
| 65 | zslaa0_009216 | zslaa0_009216.z1.scf | similar to CG8234PA isoform A *Tribolium castaneum* | LNO | substrate-specific transmembrane transporter activity | 9.4 |
| 66 | LPU1859 | LPU1859-2007-09-05.ab1 | AGAP012390-PA [*Anopheles gambiae* str. PEST] | LPU | Unknown function | 9.3 |
| 67 | CL1518 Contig1 | zslaa0_000589.z1.scf; LNC1886-2008-01-14.ab1 | GE24914 [*Drosophila yakuba*] | LNO, LNC | chitin metabolic process | 9.2 |
| 68 | LF1332 | LF1332-2007-05-08.ab1 | cuticular protein analogous to peritrophins 1-A [*Tribolium castaneum*] | LF | cuticula | 8.7 |
| 69 | zslaa0_017293 | zslaa0_017293.z1.scf | no hit | LNO | no hit | 8.6 |
| 70 | LF981 | LF981-2007-03-16.ab1 | GI24105 [*Drosophila mojavensis*] | LF | chitin metabolic process | 8.4 |
| 71 | LNC1885 | LNC1885-2008-01-14.ab1 | similar to CG7442PA *Tribolium castaneum* | LNC | transporter activity | 8.2 |
| 72 | zslaa0_007994 | zslaa0_007994.z1.scf | no hit | LNO | no hit | 8.2 |
| 73 | zslaa0_016808 | zslaa0_016808.z1.scf | no hit | LNO | no hit | 7.9 |
| 74 | CL3723 Contig1 | LNC2255-2008-01-15.ab1; 364_FN728_ME28_T3.ab1 | Trehalose6phosphate synthase *Aedes aegypti* (Yellowfever mosquito) | ME, LNC | Embryonal development | 7.9 |
| 75 | NLG907 | NLG907-2007-06-21.ab1 | no hit | NLG | no hit | 7.6 |
| 76 | NLG2619 | NLG2619-2007-09-10.ab1 | vitellogenin-like protein [*Lepeophtheirus salmonis*] | NLG | Lipid transporter activity | 7.5 |
| 77 | zslaa0_000863 | zslaa0_000863.z1.scf | no hit | LNO | no hit | 7.4 |
| 78 | CL474 Contig1 | zslaa0_002307.z1.scf; LNC516-2007-12-20 .ab1; LNO937-2007-02-13 .ab1; LPU1852-2007-09-05 .ab1; HA863-2005-02-25 .ab1; LNO2177-2007-03-06 .ab1; LF1506-2007-05-08 .ab1; LPU1835-2007-09-05.ab1 | no hit | LF, LPU, HA, LNO, LNC | no hit | 7.3 |
| 79 | zslaa0_011523 | zslaa0_011523.z1.scf | Zgc:162888 protein *Danio rerio* (Zebrafish) (*Brachydanio rerio*) | LNO | metabolic | 7.3 |
| 80 | CL734 Contig1 | zslaa0_003981.z1.scf; zslaa0_003737.z1.scf; zslaa0_003576.z1.scf; zslaa0_011358.z1.scf; zslaa1_010192.z1.scf; zslaa0_011370.z1.scf; zslaa0_002144.z1.scf | no hit | LNO | no hit | 7.1 |
| 81 | LNO2065 | LNO2065-2007-02-20.ab1 | no hit | LNO | no hit | 7.1 |
| 82 | LNO2649 | LNO2649-2007-03-01.ab1 | no hit | LNO | no hit | 7.0 |
| 83 | zslaa0_009258 | zslaa0_009258.z1.scf | no hit | LNO | no hit | 7.0 |
| 84 | CL2079 Contig1 | LPU2018-2007-09-06 .ab1; LNC3784-2009-03-06 .ab1; LN01250-2007-02-08.ab1 | Sodiumand chloride dependent neurotransmitter transporter *Aedes aegypti* (Yellowfever mosquito) | LPU, LNO, LNC | signaling | 6.8 |
| 85 | CL260 Contig1 | zslaa0_011031.z1.scf; zslaa0_006447.z1.scf; zslaa0_006370.z1.scf; zslaa0_013028.z1.scf; zslaa0_001447.z1.scf; zslaa0_005814.z1.scf; zslaa0_013708.z1.scf; zslaa0_011803.z1.scf; zslaa1_010101.z1.scf; zslaa0_008516.z1.scf; zslaa0_001425.z1.scf; LPU1157-2007-08-29.ab1; LNO3159-2007-02-27.ab1; LNC2142-2008-01-15.ab1 | no hit | LPU, LNO, LNC | no hit | 6.7 |
| 86 | CL3132 Contig1 | NLG1730-2007-08-23 .ab1; NLG3283-2008-12-22.ab1 | no hit | NLG | no hit | 6.5 |
| 87 | HA934 | HA934-2005-03-02.ab1 | Homolog of Homo sapiens "CCR4 carbon catabolite repression 4like *Takifugu rubripes* | HA | Transcription regulation | 6.5 |
| 88 | CL3720 Contig1 | L1T459-2006-12-19.ab1; L1T790-2006-12-21.ab1 | no hit | L1T | no hit | 6.2 |
| 89 | CL1324 Contig1 | zslaa0_014445.z1.scf; HA876-2005-02-25 .ab1; zslaa0_010562.z1.scf; LF1424-2007-04-23.ab1 | no hit | LF, HA, LNO | no hit | 6.0 |
| 90 | LNC203 | LNC203-2007-12-18.ab1 | Hypoxanthine phosphoribosyltransferase 1 *Xenopus tropicalis* (Western clawed frog) (*Silurana tropicalis*) | LNC | Purine salvage | 5.8 |
| 91 | zslaa0_006750 | zslaa0_006750.z1.scf | no hit | LNO | no hit | 5.8 |
| 92 | CL583 Contig1 | zslaa0_000590.z1.scf; zslaa0_011846.z1.scf; zslaa0_014949.z1.scf; zslaa0_017742.z1.scf; zslaa0_018462.z1.scf; zslaa0_005453.z1.scf; NLG3189-2007-09-27 .ab1; LNO3254-2007-02-15.ab1 | no hit | LNO, NLG | no hit | 5.8 |
| 93 | LNC2718 | LNC2718-2008-01-16.ab1 | no hit | LNC | no hit | 5.7 |
| 94 | zslaa0_015021 | zslaa0_015021.z1.scf | no hit | LNO | no hit | 5.7 |
| 95 | CL2540 Contig1 | zslaa0_005514.z1.scf; zslaa0_016955.z1.scf | Homolog of *Brachydanio rerio* Betaureidopropionase. *Takifugu rubripes* | LNO | pyrimidine base metabolic process | 5.7 |
| 96 | FN4141 | FN4141-2003-02-18.ab1 | no hit | ME | no hit | 5.7 |
| 97 | zslaa0_005569 | zslaa0_005569.z1.scf | no hit | LNO | no hit | 5.6 |
| 98 | CL2962 Contig1 | zslaa0_006863.z1.scf; LPU1734-2007-09-05.ab1 | similar to Phosphomannomutase 45A CG8073PA isoform A *Apis mellifera* | LPU, LNO | carbohydrate metabolic process | 5.5 |
| 99 | CL983 Contig1 | zslaa0_017223.z1.scf; zslaa0_004574.z1.scf; zslaa0_015144.z1.scf; zslaa0_016218.z1.scf; LF1667-2007-04-23.ab1; L1T1531-2007-02-26.ab1 | Cuticle protein putative *Aedes aegypti* (Yellowfever mosquito) | L1T, LF, LNO | cuticula | 5.5 |
| 100 | zslaa0_010567 | zslaa0_010567.z1.scf | similar to CG3057PA isoform A *Tribolium castaneum* (Congested-like trachea protein) | LNO | development | 5.5 |
| 101 | zslaa0_011136 | zslaa0_011136.z1.scf | AGAP008310-PA [*Anopheles gambiae str*. PEST] | LNO | unknown function | 5.3 |
| 102 | CL640 Contig1 | zslaa0_003639.z1.scf; zslaa0_010924.z1.scf; zslaa0_009847.z1.scf; LF112-2007-01-09.ab1; PU884-2005-03-14.ab1; LF927-2007-03-16.ab1 | CG14607PA *Drosophila melanogaster* (Fruit fly) | PU, LF, LNO | chitin metabolic process | 5.3 |
| 103 | LNC1655 | LNC1655-2008-01-14.ab1 | amine oxidase [*Culex quinquefasciatus*] | LNC | oxidation-reduction process | 5.3 |
| 104 | zslaa0_012412 | zslaa0_012412.z1.scf | no hit | LNO | no hit | 5.3 |
| 105 | L1T592 | L1T592-2006-12-21.ab1 | no hit | L1T | no hit | 5.2 |
| 106 | LPU1892 | LPU1892-2007-09-05.ab1 | poly(U)-specific endoribonuclease isoform 3 precursor [*Homo sapiens*] | LPU | endoribonuclease activity | 5.2 |
| 107 | CL3555 Contig1 | HA831-2005-02-25.ab1; HA567-2005-02-14.ab1 | no hit | HA | no hit | 5.2 |
| 108 | CL1587 Contig1 | LPU252-2007-07-30 .ab1; LNC3365-2009-02-09 .ab1; HAV-FN-FN2849-2001-12-19.ab1 | ABU41084.1 hypothetical protein [*Lepeophtheirus salmonis*] | ME, LPU, LNC | Unknown function | 5.0 |
| 109 | CL2432 Contig1 | zslaa0_009192.z1.scf; PU130-2004-09-20.ab1 | no hit | PU, LNO | no hit | 5.0 |
| 110 | CL2787 Contig1 | zslaa0_012673.z1.scf; zslaa0_008176.z1.scf | no hit | LNO | no hit | 5.0 |
| 111 | CL42 Contig2 | HAV-FN-FN2542-2001-12-13.ab1; HAV-FN-FN2542-rerun-2001-12-16.ab1; 383_FN895_ME195_T3.ab1; FN3534-2002-03-25.ab1; FN4539-2003-03-05.ab1; FN3203-2002-03-08.ab1; FN4331-2003-02-19.ab1; HAV-FN-FN2642-2001-12-13.ab1; FN3388-2002-03-08.ab1; FN4959-2003-03-12.ab1; FN3712-2003-01-16.ab1; 364_FN715_ME15_T3.ab1; FN6063-2005-04-05.ab1; FN4233-2003-02-18.ab1; FN6030-2005-04-05.ab1; LF1073-2007-03-30.ab1; LNC3067-2008-01-16.ab1; LF1338-2007-05-08.ab1 | sortilin-related receptor-like *[Xenopus (Silurana)*  *tropicalis]* | ME, LF, LNC | Steroid metabolism | 4.9 |
| 112 | FN6128 | FN6128-2005-04-05.ab1 | similar to Phosphoglucomutase 2 *Ornithorhynchus anatinus* | ME | Carbohydrate metabolism | 4.9 |
| 113 | CL2951 Contig1 | L1T566-2006-12-21.ab1; zslaa0_003683.z1.scf | Laminin A chain putative *Aedes aegypti* (Yellowfever mosquito) | L1T, LNO | development | 4.9 |
| 114 | zslaa0_015322 | zslaa0_015322.z1.scf | no hit | LNO | no hit | 4.9 |
| 115 | LF659 | LF659-2007-01-11.ab1 | no hit | LF | no hit | 4.9 |
| 116 | CL1833 Contig1 | zslaa0_004171.z1.scf; zslaa0_018446.z1.scf; LNO3025-2007-02-19.ab1 | no hit | LNO | no hit | 4.9 |
| 117 | LNO3356 | LNO3356-2007-02-19.ab1 | CG6330PA isoform A *Drosophila melanogaster* (Fruit fly) | LNO | nucleotide catabolism | 4.9 |
| 118 | zslaa0_004425 | zslaa0_004425.z1.scf | CG6330PA isoform A *Drosophila melanogaster* (Fruit fly) | LNO | nucleotide catabolism | 4.8 |
| 119 | LNC582 | LNC582-2007-12-20.ab1 | Cytochrome P450 *CYP330A1 Carcinus maenas* (Common shore crab) (Green crab) | LNC | monooxygenase activity | 4.8 |
| 120 | CL1290 Contig1 | LNO512-2007-02-13.ab1; LF936-2007-03-16.ab1; LPU304-2007-08-29.ab1 | no hit | LF, LPU, LNO | no hit | 4.8 |
| 121 | CL42 Contig3 | LNO505-2007-02-13 .ab1; LPU846-2007-08-29 .ab1; LNO2547-2007-03-01 .ab1; LNO2370-2007-02-27 .ab1; LNO3251-2007-02-15 .ab1; zslaa0_016519.z1.scf; NLG3076-2007-09-26 .ab1; zslaa0_014051.z1.scf | similar to CG33950PD isoform D *Apis mellifera* (Terribly reduced optic lobes, isoform D) | LPU, LNO, NLG | Cell adhesion | 4.8 |
| 122 | zslaa0_011371 | zslaa0_011371.z1.scf | no hit | LNO | no hit | 4.8 |
| 123 | LNC1876 | LNC1876-2008-01-14.ab1 | no hit | LNC | no hit | 4.7 |
| 124 | CL2512 Contig1 | zslaa0_011478.z1.scf; zslaa0_006982.z1.scf | no hit | LNO | no hit | 4.7 |
| 125 | CL2579 Contig1 | zslaa0_012472.z1.scf; L1T1882-2007-01-24.ab1 | CHKov1 [*Culex quinquefasciatus*] | L1T, LNO | RNA-directed DNA polymerase activity | 4.6 |
| 126 | LNO3187 | LNO3187-2007-02-27.ab1 | no hit | LNO | no hit | 4.5 |
| 127 | NLG1724 | NLG1724-2007-08-23.ab1 | similar to CG2811PA isoform 2 *Apis mellifera* | NLG | Putative gamma-glutamylcyclotransferase | 4.5 |
| 128 | CL2847 Contig1 | LNO3177-2007-02-27 .ab1; HA766-2005-02-25.ab1 | similar to Glucose transporter 1 CG1086PB isoform B isoform 1 *Apis mellifera* | HA, LNO | Glucose transporter activity | 4.4 |
| 129 | CL502 Contig1 | zslaa0_003746.z1.scf; zslaa0_015307.z1.scf; zslaa0_016649.z1.scf; zslaa0_005450.z1.scf; zslaa0_013587.z1.scf; LF224-2007-01-09 .ab1; LNO1932-2007-02-19 .ab1; LPU919-2007-08-29 .ab1; LNO1553-2007-02-07.ab1 | similar to LDLa domain containing chitin binding protein 1 CG8756PC isoform C isoform 1 *Apis mellifera* | LF, LPU, LNO | chitin metabolic process | 4.4 |
| 130 | CL1925 Contig1 | LNC2161-2008-01-15 .ab1; LNC2542-2008-01-15 .ab1; LNC1784-2008-01-24.ab1 | Cytochrome P450 3A31 *Mesocricetus auratus* (Golden hamster) | LNC | monooxygenase activity | 4.4 |
| 131 | CL3527 Contig1 | LNC2621-2008-01-15.ab1; LNO3917-2007-02-27.ab1 | no hit | LNO, LNC | no hit | 4.4 |
| 132 | zslaa0_016533 | zslaa0_016533.z1.scf | no hit | LNO | no hit | 4.4 |
| 133 | CL3785 Contig1 | zslaa0_004248.z1.scf; LPU1913-2007-09-05.ab1 | Cytochrome P450 *Anopheles gambiae* (African malaria mosquito) | LPU, LNO | monooxygenase activity | 4.4 |
| 134 | LNO1861 | LNO1861-2007-02-16.ab1 | sodium bicarbonate transporter 4 isoform c *Xenopus tropicalis* | LNO | anion transport | 4.3 |
| 135 | LPU1341 | LPU1341-2007-09-12.ab1 | no hit | LPU | no hit | 4.3 |
| 136 | LF779 | LF779-2007-03-16.ab1 | methylenetetrahydrofolate dehydrogenase 1-like [*Saccoglossus*  *kowalevskii*] | LF | One-carbon metabolism | 4.3 |
| 137 | NLG1427 | NLG1427-2007-08-22.ab1 | no hit | NLG | no hit | 4.2 |
| 138 | CL1631 Contig1 | zslaa0_009803.z1.scf; LNO1863-2007-02-16.ab1; LNC839-2007-12-21.ab1; LNC862-2007-12-21.ab1 | Novel KRAB box and zinc finger C2H2 type domain containing protein *Mus musculus* (Mouse) | LNO, LNC | transcription regulation | 4.2 |
| 139 | CL2764 Contig1 | zslaa0_010610.z1.scf; LF1505-2007-05-08.ab1 | NAD dependent epimerase/dehydratase [*Schistosoma mansoni*] | LF, LNO | cellular metabolic process | 4.2 |
| 140 | zslaa0_009859 | zslaa0_009859.z1.scf | Spondin-1 [*Harpegnathos saltator*] | LNO | cell adhesion | 4.2 |
| 141 | zslaa0_005725 | zslaa0_005725.z1.scf | no hit | LNO | no hit | 4.2 |
| 142 | zslaa0_010035 | zslaa0_010035.z1.scf | similar to CG3322PA *Tribolium castaneum*(Laminin subunit gamma-1) | LNO | cell adhesion | 4.2 |
| 143 | LF1681 | LF1681-2007-04-23.ab1 | ABU41084.1 hypothetical protein [*Lepeophtheirus salmonis*] | LF | Unknown function | 4.1 |
| 144 | CL3772 Contig1 | LPA126-2007-05-15 .ab1; LPA176-2007-05-15.ab1 | similar to CG3456PA *Tribolium castaneum* | LPA | transmembrane transport | 4.1 |
| 145 | HA294 | HA294-2004-08-06.ab1 | similar to Glucose transporter 1 CG1086PB isoform B isoform 1 *Apis mellifera* | HA | Glucose transporter activity | 4.1 |
| 146 | LNO3312 | LNO3312-2007-02-19.ab1 | no hit | LNO | no hit | 4.1 |
| 147 | CL1513 Contig1 | LNO2949-2007-03-07 .ab1; LPU406-2007-09-05 .ab1; HA370-2004-08-13.ab1 | AGAP011937-PA [Anopheles gambiae str. PEST] | LPU, HA, LNO | chitin metabolic process | 4.1 |
| 148 | CL2053 Contig1 | zslaa0_010836.z1.scf; zslaa0_017295.z1.scf; zslaa0_016087.z1.scf | GH25683p *Drosophila melanogaster* (Fruit fly) | LNO | monovalent inorganic cation transport | 4.1 |
| 149 | NLG3113 | NLG3113-2007-09-27.ab1 | no hit | NLG | no hit | 4.1 |
| 150 | NLG2227 | NLG2227-2007-09-10.ab1 | no hit | NLG | no hit | 4.0 |
| 151 | LNO1420 | LNO1420-2007-02-14.ab1 | no hit | LNO | no hit | 4.0 |
| 152 | zslaa0_000861 | zslaa0_000861.z1.scf | no hit | LNO | no hit | 4.0 |
| 153 | LPU1952 | LPU1952-2007-09-05.ab1 | no hit | LPU | no hit | 4.0 |
| 154 | CL2763 Contig1 | zslaa0_008669.z1.scf; zslaa0_013109.z1.scf | similar to alpha2macroglobulinlike 1 *Apis mellifera* | LNO | Protease inhibitor | 3.9 |
| 155 | zslaa0_007650 | zslaa0_007650.z1.scf | no hit | LNO | no hit | 3.9 |
| 156 | CL5 Contig2 | PU824-2005-03-14 .ab1; PU854-2005-03-14 .ab1; HA722-2005-02-25 .ab1; LF1655-2007-04-23 .ab1; zslaa0_016703.z1.scf | ABU41030.1, hypothetical protein [*Lepeophtheirus salmonis*] | PU, LF, HA, LNO | Unknown function | 3.9 |
| 157 | zslaa0_000718 | zslaa0_000718.z1.scf | no hit | LNO | no hit | 3.9 |
| 158 | LF1306 | LF1306-2007-05-08.ab1 | Coagulation factor VII *Fugu rubripes* (Japanese pufferfish) (*Takifugu rubripes*) | LF | Blood coagulation | 3.9 |
| 159 | LNC521 | LNC521-2007-12-20.ab1 | no hit | LNC | no hit | 3.9 |
| 160 | LNC905 | LNC905-2007-12-28.ab1 | no hit | LNC | no hit | 3.9 |
| 161 | NLG1435 | NLG1435-2007-08-22.ab1 | similar to Inosine5monophosphate dehydrogenase (IMP dehydrogenase) (IMPDH) (IMPD) (Protein raspberry) isoform 1 *Apis mellifera* | NLG | nucleotide biosynthesis | 3.9 |
| 162 | CL856 Contig1 | FN5881-2005-03-14 .ab1; FN4060-2003-02-18 .ab1; HAV-FN-FN2117-2001-11-30 .ab1; FN5827-2005-03-14 .ab1; FN4756-2003-03-06 .ab1; FN4022-2003-02-18.ab1 | no hit | ME | no hit | 3.9 |
| 163 | zslaa0_012727 | zslaa0_012727.z1.scf | no hit | LNO | no hit | 3.9 |
| 164 | CL1249 Contig1 | LNC1150-2008-01-17 .ab1; HAV-FN-FN2347-rerun-2001-12-11 .ab1; LNC848-2007-12-21 .ab1; HAV-FN-FN2347-2001-12-10.ab1 | Congested-like trachea protein [*Lepeophtheirus salmonis*] | ME, LNC | Transport, development | 3.8 |
| 165 | LPU1264 | LPU1264-2007-09-04.ab1 | GK10820 [Drosophila willistoni] | LPU | chitin metabolic | 3.8 |
| 166 | CL1255 Contig1 | zslaa0_016347.z1.scf; LNO4042-2007-03-09.ab1; LNO2349-2007-02-27.ab1 | Aldehyde dehydrogenase *Aedes aegypti* (Yellowfever mosquito) | LNO | oxidation reduction | 3.8 |
| 167 | CL3275 Contig1 | LNO1439-2007-02-14 .ab1; LNO584-2007-02-13.ab1 | Cytochrome 3A72 *Balaenoptera acutorostrata* (Minke whale) (Lesser rorqual) | LNO | monooxygenase activity | 3.8 |
| 168 | CL1414 Contig1 | HA984-2005-03-02 .ab1; PU383-2004-09-28 .ab1; PU804-2005-03-14 .ab1; LNO3133-2007-02-27.ab1 | no hit | PU, HA, LNO | no hit | 3.8 |
| 169 | LNC2337 | LNC2337-2008-01-15.ab1 | similar to CG31075PA *Tribolium castaneum* | LNC | aldehyde dehydrogenase | 3.8 |
| 170 | CL1104 Contig1 | zslaa0_009143.z1.scf; zslaa0_008565.z1.scf; zslaa0_012112.z1.scf; LPU1579-2007-09-04.ab1 | CG14275PA isoform A *Drosophila melanogaster* (Fruit fly) | LPU, LNO | unknown function | 3.8 |
| 171 | NLG1741 | NLG1741-2007-08-23.ab1 | no hit | NLG | no hit | 3.7 |
| 172 | zslaa0_016027 | zslaa0_016027.z1.scf | no hit | LNO | no hit | 3.7 |
| 173 | NLG895 | NLG895-2007-06-14.ab1 | no hit | NLG | no hit | 3.7 |
| 174 | CL3404 Contig1 | NLG4420-2009-03-05 .ab1; LNC1660-2008-01-14.ab1 | similar to 24dehydrocholesterol reductase *Tribolium castaneum* | LNC, NLG | development | 3.7 |
| 175 | LNC525 | LNC525-2007-12-20.ab1 | no hit | LNC | no hit | 3.7 |
| 176 | CL115 Contig3 | zslaa0_001837.z1.scf; zslaa0_012372.z1.scf; zslaa0_014619.z1.scf; LPU207-2007-07-30.ab1; LNO3878-2007-03-15.ab1; zslaa0_016186.z1.scf; zslaa0_011563.z1.scf; LNO1651-2007-02-15.ab1; LNO2824-2007-03-07.ab1; LNC1027-2007-12-28.ab1; PU515-2004-09-30.ab1 | Prophenoloxidase activating factor *Penaeus monodon* (Penoeid shrimp) | PU, LPU, LNO, LNC | serine-type endopeptidase activity | 3.7 |
| 177 | LNO923 | LNO923-2007-02-13.ab1 | LOC398553 protein *Xenopus laevis* (African clawed frog) | LNO | nucleotide metabolic | 3.7 |
| 178 | CL265 Contig1 | zslaa0_003027.z1.scf; zslaa0_000741.z1.scf; zslaa0_003571.z1.scf; LNO3448-2007-03-05 .ab1; LNC461-2007-12-21 .ab1; LNC3761-2009-03-06 .ab1; LPU1357-2007-09-12 .ab1; zslaa0_007642.z1.scf; LPU2040-2007-09-06 .ab1; FN4719-2003-03-06 .ab1; LNO3236-2007-02-15.ab1 | Glutamine synthetase *Anopheles gambiae str*. PEST | ME, LPU, LNO, LNC | Amino acid biosynthesis | 3.7 |
| 179 | CL3537 Contig1 | zslaa0_017194.z1.scf; zslaa0_015262.z1.scf | haloacid dehalogenase-like hydrolase family protein [*Brugia malayi*] | LNO | phosphoglycolate phosphatase activity | 3.7 |
| 180 | CL2664 Contig1 | zslaa0_002678.z1.scf; LNC2361-2008-01-15.ab1 | Solute carrier family 25 (Mitochondrial oxodicarboxylate carrier) member 21 *Danio rerio* (Zebrafish) (*Brachydanio rerio*) | LNO, LNC | transporter activity | 3.7 |
| 181 | CL1802 Contig1 | zslaa0_009979.z1.scf; LNC723-2007-12-20.ab1; L1T1313-2007-01-23.ab1 | similar to GTP cyclohydrolase I precursor (GTPCHI) *Strongylocentrotus purpuratus* | L1T, LNO, LNC | tetrahydrofolate biosynthetic | 3.7 |
| 182 | zslaa0_017009 | zslaa0_017009.z1.scf | Putative uncharacterized protein CBG14689 *Caenorhabditis briggsae* | LNO | nucleotide biosynthesis | 3.7 |
| 183 | CL1289 Contig1 | zslaa0_013802.z1.scf; zslaa0_012229.z1.scf; LNO2836-2007-03-07 .ab1; FN3589-2002-03-25.ab1 | similar to Putative deoxyribosephosphate aldolase (Phosphodeoxyriboaldolase) (Deoxyriboaldolase) (DERA) *Tribolium castaneum* | ME, LNO | deoxyribonucleoside catabolic process | 3.6 |
| 184 | CL3827 Contig1 | LNC1727-2008-01-24.ab1; L1T1635-2007-01-12.ab1 | no hit | L1T, LNC | no hit | 3.6 |
| 185 | NLG114 | NLG114-2007-05-14.ab1 | no hit | NLG | no hit | 3.6 |
| 186 | zslaa0_016232 | zslaa0_016232.z1.scf | similar to fibulin 1 and [*Tribolium castaneum*] | LNO | extracellular matrix organization | 3.6 |
| 187 | NLG2557 | NLG2557-2007-09-17.ab1 | no hit | NLG | no hit | 3.6 |
| 188 | LNC1949 | LNC1949-2008-01-15.ab1 | no hit | LNC | no hit | 3.6 |
| 189 | LNO2420 | LNO2420-2007-02-14.ab1 | no hit | LNO | no hit | 3.6 |
| 190 | CL3213 Contig1 | 354_FN496_SE14_T7.ab1; 354_FN493_SE11_T7.ab1 | similar to CG5254PA *Tribolium castaneum* | SB | salivary gland cell autophagic cell death | 3.6 |
| 191 | LNC3020 | LNC3020-2008-01-16.ab1 | no hit | LNC | no hit | 3.6 |
| 192 | zslaa0_015922 | zslaa0_015922.z1.scf | no hit | LNO | no hit | 3.6 |
| 193 | CL2988 Contig1 | zslaa0_003259.z1.scf; LNO863-2007-01-03.ab1 | hypothetical protein [*Schistosoma mansoni*] | LNO | unknown function | 3.5 |
| 194 | CL1398 Contig1 | zslaa0_012747.z1.scf; LF1074-2007-03-30.ab1; zslaa0_017116.z1.scf; LNO148-2006-12-01.ab1 | no hit | LF, LNO | no hit | 3.5 |
| 195 | FN6169 | FN6169-2005-04-05.ab1 | no hit | ME | no hit | 3.5 |
| 196 | L1T1786 | L1T1786-2007-01-24.ab1 | Peroxinectin precursor *Pacifastacus leniusculus* (Signal crayfish) | L1T | stress response | 3.5 |
| 197 | zslaa0_016026 | zslaa0_016026.z1.scf | no hit | LNO | no hit | 3.5 |
| 198 | CL343 Contig1 | zslaa0_014902.z1.scf; zslaa0_009852.z1.scf; zslaa0_019273.z1.scf; zslaa0_008053.z1.scf; zslaa0_0097_F09.ab1; HAV-FN-FN2371-2001-12-10.ab1; NLG2073-2007-08-03.ab1; LNC2929-2008-01-16.ab1; LNO3122-2007-02-27.ab1 | no hit | ME, LNO, LNC, NLG | no hit | 3.5 |
| 199 | CL3604 Contig1 | LNO2231-2007-02-27 .ab1; HA762-2005-02-25.ab1 | Homolog of Homo sapiens "transmembrane protein 38B *Takifugu rubripes* | HA, LNO | ion transporter activity | 3.5 |
| 200 | CL1373 Contig1 | zslaa0_012333.z1.scf; zslaa0_012283.z1.scf; LNO1655-2007-02-15.ab1; L1T1562-2007-02-26.ab1 | no hit | L1T, LNO | no hit | 3.5 |
| 201 | LNC237 | LNC237-2007-12-18.ab1 | no hit | LNC | no hit | 3.5 |
| 202 | LF407 | LF407-2007-01-10.ab1 | GA11509PA *Drosophila pseudoobscura* (Fruit fly) | LF | transporter activity | 3.5 |
| 203 | LF772 | LF772-2007-03-16.ab1 | Phosphoglucomutase 1 *Danio rerio* (Zebrafish) (*Brachydanio rerio*) | LF | Carbohydrate metabolism | 3.4 |
| 204 | FN1219 | 482_FN1219_C03_018.ab1 | Granulins precursor (Proepithelin) (PEPI) (PC cellderived growth factor) (PCDGF) *Mus musculus* (Mouse) | FB | reproduction | 3.4 |
| 205 | L1T101 | L1T101-2006-12-01.ab1 | no hit | L1T | no hit | 3.4 |
| 206 | LF1584 | LF1584-2007-05-08.ab1 | no hit | LF | no hit | 3.4 |
| 207 | LNO478 | LNO478-2007-02-08.ab1 | no hit | LNO | no hit | 3.4 |
| 208 | CL3215 Contig1 | zslaa1_010193.z1.scf; LNO3922-2007-02-27.ab1 | similar to CG18547PA *Apis mellifera* | LNO | oxidation reduction | 3.4 |
| 209 | LPU1008 | LPU1008-2007-08-31.ab1 | no hit | LPU | no hit | 3.4 |
| 210 | CL1444 Contig1 | LNO545-2007-02-13 .ab1; LF1980-2007-05-09 .ab1; zslaa0_008932.z1.scf; LNO2630-2007-03-01.ab1 | similar to GTPase Rab8b *Gallus gallus* | LF, LNO | small GTPase mediated signal transduction | 3.4 |
| 211 | LNO3096 | LNO3096-2007-02-19.ab1 | no hit | LNO | no hit | 3.4 |
| 212 | LNO2936 | LNO2936-2007-03-07.ab1 | similar to Calcium/calmodulindependent protein kinase I CG1495PG isoform G *Apis mellifera* | LNO | calmodulin-dependent protein kinase activity | 3.4 |
| 213 | zslaa0_015338 | zslaa0_015338.z1.scf | no hit | LNO | no hit | 3.4 |
| 214 | CL3794 Contig1 | NLG984-2007-06-21 .ab1; LNC3003-2008-01-16.ab1 | Bipolar kinesin KRP-130 [*Harpegnathos saltator*] | LNC, NLG | cell division | 3.4 |
| 215 | zslaa0_015312 | zslaa0_015312.z1.scf | no hit | LNO | no hit | 3.4 |
| 216 | zslaa0_014311 | zslaa0_014311.z1.scf | no hit | LNO | no hit | 3.4 |
| 217 | CL2841 Contig1 | LNC2328-2008-01-15.ab1; zslaa0_013603.z1.scf | similar to alpha 5 type IV collagen isoform 1 precursor *Tribolium castaneum* | LNO, LNC | extracellular matrix structural constituent | 3.3 |
| 218 | NLG372 | NLG372-2007-06-04.ab1 | no hit | NLG | no hit | 3.3 |
| 219 | zslaa0_013494 | zslaa0_013494.z1.scf | no hit | LNO | no hit | 3.3 |
| 220 | L1T426 | L1T426-2006-12-19.ab1 | no hit | L1T | no hit | 3.3 |
| 221 | FN2659 | HAV-FN-FN2659-2001-12-13.ab1 | no hit | ME | no hit | 3.3 |
| 222 | L1T1056 | L1T1056-2007-01-02.ab1 | similar to CG1240PA *Tribolium castaneum* | L1T | unknown function | 3.3 |
| 223 | CL543 Contig1 | zslaa0_007267.z1.scf; zslaa0_011687.z1.scf; zslaa0_018414.z1.scf; zslaa0_009239.z1.scf; zslaa0_007583.z1.scf; LNO1761-2007-02-08.ab1; PU220-2004-09-21.ab1; PU156-2004-09-20.ab1 | Peritrophin-1 [*Lepeophtheirus salmonis*] | PU, LNO | chitin metabolic process | 3.3 |
| 224 | CL2614 Contig1 | FN4716-2003-03-06 .ab1; FN4110-2003-02-18.ab1 | Zgc:162153 protein *Danio rerio* (Zebrafish) (Brachydanio rerio), sphingosine-1-phosphate lyase 1 | ME | carboxylic acid metabolic process | 3.3 |
| 225 | zslaa0_015492 | zslaa0_015492.z1.scf | similar to pyridoxal kinase partial *Strongylocentrotus purpuratus* | LNO | pyridoxine biosynthetic | 3.3 |
| 226 | CL3004 Contig1 | FN4843-2003-03-12 .ab1; LNO1428-2007-02-14.ab1 | no hit | ME, LNO | no hit | 3.3 |
| 227 | CL672 Contig1 | zslaa0_006212.z1.scf; zslaa0_009187.z1.scf; zslaa0_013396.z1.scf; LPU2053-2007-09-06 .ab1; LNO1443-2007-02-14 .ab1; LNC141-2007-11-22.ab1 | no hit | LPU, LNO, LNC | no hit | 3.3 |
| 228 | CL2474 Contig1 | LNC366-2007-12-18 .ab1; zslaa0_008510.z1.scf | no hit | LNO, LNC | no hit | 3.3 |
| 229 | zslaa0_018413 | zslaa0_018413.z1.scf | Chromosome 7 SCAF14557 whole genome shotgun sequence *Tetraodon nigroviridis* (Green puffer) | LNO | transcription regulation | 3.3 |
| 230 | zslaa0_006739 | zslaa0_006739.z1.scf | no hit | LNO | no hit | 3.3 |
| 231 | CL2401 Contig1 | LNC3051-2008-01-16 .ab1; LNC1605-2008-01-14 .ab1; LPU280-2007-07-30.ab1 | no hit | LPU, LNC | unknown function | 3.2 |
| 232 | CL374 Contig1 | LN01309-2007-02-01 .ab1; LF862-2007-02-12 .ab1; LPU1518-2007-09-04 .ab1; LPU1525-2007-09-04 .ab1; L1T1083-2007-01-02 .ab1; LIT384-2006-12-18 .ab1; LIT374-2006-12-18 .ab1; HA865-2005-02-25.ab1 | similar to Ras suppressor1 CG9031PA *Apis mellifera* | L1T, LF, LPU, HA, LNO | signal transduction | 3.2 |
| 233 | LNC2783 | LNC2783-2008-01-16.ab1 | no hit | LNC | no hit | 3.2 |
| 234 | LNC2368 | LNC2368-2008-01-15.ab1 | similar to CG6199PA isoform A *Tribolium castaneum* | LNC | oxidation reduction | 3.2 |
| 235 | CL2980 Contig1 | zslaa0_017255.z1.scf; zslaa0_015827.z1.scf | no hit | LNO | no hit | 3.2 |
| 236 | CL749 Contig2 | FN5554-2005-02-10 .ab1; LNO2171-2007-03-06 .ab1; LN01287-2007-02-08 .ab1; HAV-FN-FN2607-2001-12-13.ab1 | Major vault protein *Strongylocentrotus purpuratus* (Purple sea urchin) | ME, LNO | drug resistance | 3.2 |
| 237 | NLG187 | NLG187-2007-05-14.ab1 | similar to Probable cytochrome P450 9f2 (CYPIXF2) *Tribolium castaneum* | NLG | May be involved in the metabolism of insect hormones and in the breakdown of synthetic insecticides | 3.2 |
| 238 | NLG2564 | NLG2564-2007-09-17.ab1 | no hit | NLG | no hit | 3.2 |
| 239 | CL32 Contig4 | zslaa0_007504.z1.scf; zslaa0_007151.z1.scf; FN5723-2005-02-14 .ab1; zslaa0_005578.z1.scf; zslaa0_003991.z1.scf; zslaa0_002958.z1.scf; zslaa0_009427.z1.scf; zslaa0_0097_G03 .ab1; LNC2225-2008-01-15 .ab1; LPU1166-2007-08-29 .ab1; LF554-2007-01-10 .ab1; zslaa1_010149.z1.scf; LNO3841-2007-03-15 .ab1; FN4290-2003-02-18 .ab1; FN3343-2002-03-08 .ab1; FN3371-2002-03-08.ab1 | no hit | ME, LF, LPU, LNO, LNC | no hit | 3.2 |
| 240 | zslaa0_012308 | zslaa0_012308.z1.scf | similar to Mlx interactor CG18362PC isoform C *Apis mellifera* | LNO | transcription regulator activity | 3.2 |
| 241 | CL3628 Contig1 | LF495-2007-01-10 .ab1; LNC2501-2008-01-15.ab1 | no hit | LF, LNC | no hit | 3.2 |
| 242 | CL2168 Contig1 | zslaa0_014977.z1.scf; zslaa0_006056.z1.scf; zslaa0_009917.z1.scf | Phosphoglucomutase1 *Mus musculus* (Mouse) | LNO | glycolysis/ gluconeogenesis | 3.2 |
| 243 | LNO3433 | LNO3433-2007-03-05.ab1 | no hit | LNO | no hit | 3.2 |
| 244 | zslaa0_016007 | zslaa0_016007.z1.scf | spermidine/spermine N1acetyl transferaselike 1 *Xenopus tropicalis* | LNO | Acyltransferase | 3.2 |
| 245 | FN2816 | HAV-FN-FN2816-2001-12-19.ab1 | no hit | ME | no hit | 3.2 |
| 246 | CL413 Contig1 | LNO294-2007-01-02.ab1; zslaa0_010854.z1.scf; zslaa0_008155.z1.scf; zslaa0_002344.z1.scf; zslaa0_010446.z1.scf; zslaa0_013211.z1.scf; zslaa0_000348.z1.scf; zslaa0_010461.z1.scf; LNO3301-2007-02-19.ab1; LNO4025-2007-03-09.ab1 | Phosphatidylinositol-4-phosphate 5-kinase 8 [*Lepeophtheirus salmonis*] | LNO | 1-phosphatidylinositol-4-phosphate 5-kinase activity | 3.2 |
| 247 | CL3043 Contig1 | zslaa0_011469.z1.scf; NLG1817-2007-08-23.ab1 | similar to Chain A Structure Of Human 5Deoxy5Methylthioadenosine Phosphorylase At 1.7 A Resolution *Equus caballus* | LNO, NLG | nucleoside metabolic process | 3.2 |
| 248 | CL950 Contig1 | zslaa0_012795.z1.scf; zslaa0_001910.z1.scf; zslaa0_016386.z1.scf; zslaa0_012165.z1.scf; LNO2971-2007-03-07 .ab1; LF1254-2007-04-23.ab1 | similar to RAB27A protein isoform 1 *Apis mellifera* | LF, LNO | signal transduction | 3.2 |
| 249 | LF593 | LF593-2007-01-10.ab1 | no hit | LF | no hit | 3.1 |
| 250 | NLG976 | NLG976-2007-06-21.ab1 | similar to ATPbinding cassette subfamily B (MDR/TAP) member 1A *Tribolium castaneum* | NLG | drug resistance | 3.1 |
| 251 | zslaa0_003154 | zslaa0_003154.z1.scf | gamma-aminobutyric acid (GABA-A) receptor, subunit theta [*Mus musculus*] | LNO | chloride channel activity | 3.1 |
| 252 | CL875 Contig1 | zslaa0_014344.z1.scf; zslaa0_016550.z1.scf; zslaa0_003785.z1.scf; LPA161-2007-05-15 .ab1; zslaa0_012155.z1.scf | Elongation of very long chain fatty acids protein AAEL008004  [*Lepeophtheirus salmonis*] | LPA, LNO | Fatty acid biosynthesis | 3.1 |
| 253 | LNC1413 | LNC1413-2007-12-21.ab1 | no hit | LNC | no hit | 3.1 |
| 254 | zslaa0_008068 | zslaa0_008068.z1.scf | Microtubule associated protein *Drosophila melanogaster* (Fruit fly) | LNO | cytoskeleton | 3.1 |
| 255 | CL1267 Contig1 | zslaa0_002369.z1.scf; zslaa0_014683.z1.scf; zslaa0_009894.z1.scf | similar to MGC107801 protein *Strongylocentrotus purpuratus* | LNO | unknown function | 3.1 |
| 256 | CL816 Contig1 | zslaa1_002791.z1.scf; zslaa0_002902.z1.scf; zslaa0_015163.z1.scf; LN01257-2007-02-08.ab1; FN3391-2002-03-08.ab1; FN3928-2003-02-11.ab1; FN3928rerun-2003-02-13.ab1 | Aldehyde dehydrogenase *Aedes aegypti* (Yellowfever mosquito) | ME, LNO | oxidation reduction | 3.1 |
| 257 | CL1026 Contig1 | zslaa0_013465.z1.scf; zslaa0_004223.z1.scf; zslaa1_010188.z1.scf; LNO3706-2007-02-20.ab1; LNC189-2007-11-22.ab1 | similar to Buzidau CG13761PB *Apis mellifera* | LNO, LNC | translation initiation factor activity | 3.1 |
| 258 | LNO3467 | LNO3467-2007-03-05.ab1 | similar to ankyrin 2,3/unc44 [*Strongylocentrotus purpuratus*] | LNO | unknown function | 3.1 |
| 259 | CL3147 Contig1 | LNC2933-2008-01-16 .ab1; LPU1017-2007-08-31.ab1 | no hit | LPU, LNC | no hit | 3.1 |
| 260 | zslaa0_009617 | zslaa0_009617.z1.scf | no hit | LNO | no hit | 3.1 |
| 261 | zslaa0_010590 | zslaa0_010590.z1.scf | no hit | LNO | no hit | 3.0 |
| 262 | zslaa0_007959 | zslaa0_007959.z1.scf | no hit | LNO | no hit | 3.0 |
| 263 | CL3310 Contig1 | HAV-FN-FN1871-2001-11-28.ab1; FN4073-2003-02-18.ab1 | no hit | ME | no hit | 3.0 |
| 264 | zslaa0_004282 | zslaa0_004282.z1.scf | no hit | LNO | no hit | 3.0 |
| 265 | CL1753 Contig1 | zslaa0_014427.z1.scf; LF1908-2007-05-09 .ab1; FN6195-2005-04-05.ab1 | Cysteine-rich motor neuron 1 protein [*Lepeophtheirus salmonis*] | ME, LF, LNO | cell growth | 3.0 |
| 266 | CL2561 Contig1 | zslaa0_018431.z1.scf; LPU1519-2007-09-04.ab1 | no hit | LPU, LNO | no hit | 3.0 |
| 267 | CL1265 Contig1 | zslaa0_005125.z1.scf; LPU1757-2007-09-05 .ab1; NLG2416-2007-09-14 .ab1; FN3869-2003-02-11.ab1 | GA19257PA *Drosophila pseudoobscura* (Fruit fly) | ME, LPU, LNO, NLG | metabolic | 3.0 |
| 268 | FN4954 | FN4954-2003-03-12.ab1 | no hit | ME | no hit | 3.0 |
| 269 | LPU1541 | LPU1541-2007-09-04.ab1 | similar to Cytidine monophospho N-acetylneuraminic acid synthetase *Gallus gallus* | LPU | lipopolysaccharide biosynthetic process | 3.0 |
| 270 | CL472 Contig1 | zslaa0_010962.z1.scf; zslaa0_010584.z1.scf; zslaa0_003250.z1.scf; zslaa0_010706.z1.scf; zslaa0_012439.z1.scf; zslaa0_006043.z1.scf; zslaa0_009836.z1.scf; LPU396-2007-08-29 .ab1; FN4876-2003-03-12 .ab1; L1T_1191-2007-01-15.ab1 | Cytosolic juvenile hormone binding protein 36 kDa subunit *Bombyx mori* (Silk moth) | ME, L1T, LPU, LNO | Juvenile hormone binding | 3.0 |
| 271 | LNO628 | LNO628-2007-02-06.ab1 | Transposase *Heliothis virescens* (Noctuid moth) (Owlet moth) | LNO | transposase | 3.0 |
| 272 | CL1205 Contig1 | zslaa0_005343.z1.scf; zslaa0_014001.z1.scf; LNC2808-2008-01-15 .ab1; NLG879-2007-06-14 .ab1; NLG4695-2009-03-05.ab1 | Similarities with sp|Q8TGK9 *Saccharomyces cerevisiae* Hypothetical 6.0 kDa protein *Kluyveromyces lactis* (Yeast) (*Candida sphaerica*) | LNO, LNC, NLG | unknown function | 3.0 |
| 273 | LNO1013 | LNO1013-2007-02-01.ab1 | no hit | LNO | no hit | 3.0 |
| 274 | zslaa0_006063 | zslaa0_006063.z1.scf | no hit | LNO | no hit | 3.0 |
| 275 | NLG2553 | NLG2553-2007-09-17.ab1 | similar to ADAM metallopeptidase with thrombospondin type 1 motif 9 preproprotein *Tribolium castaneum* | NLG | development | 3.0 |
| 276 | zslaa0_015933 | zslaa0_015933.z1.scf | no hit | LNO | no hit | 3.0 |
| 277 | LF1668 | LF1668-2007-04-23.ab1 | no hit | LF | no hit | 3.0 |
| 278 | zslaa0_016522 | zslaa0_016522.z1.scf | no hit | LNO | no hit | 3.0 |
| 279 | CL1956 Contig1 | zslaa0_000158.z1.scf; zslaa0_001929.z1.scf; NLG2391-2007-09-14.ab1 | Homolog of *Homo sapiens* 2amino 3ketobutyrate coenzyme A ligase mitochondrial precursor *Takifugu rubripes* | LNO, NLG | amino acid metabolism | 3.0 |
| 280 | LNC2562 | LNC2562-2008-01-15.ab1 | Antiviral helicase SKI2 *Ajellomyces capsulatus* NAm1 | LNC | antiviral helicase | 3.0 |
| 281 | PU153 | PU153-2004-09-20.ab1 | similar to CG7187PA isoform A *Tribolium castaneum* | PU | transcription regulation | 3.0 |
| 282 | CL2613 Contig1 | FN4702-2003-03-06.ab1; FN4702rerun-2003-03-07.ab1 | Homologue of Sarcophaga 2629kDa proteinase *Periplaneta americana* (American cockroach) | ME | peptidase | 3.0 |
| 283 | zslaa0_007717 | zslaa0_007717.z1.scf | no hit | LNO | no hit | 3.0 |
| 284 | NLG1746 | NLG1746-2007-08-23.ab1 | no hit | NLG | no hit | 3.0 |
| 285 | CL2987 Contig1 | LF846-2007-02-12.ab1; LF1195-2007-05-08.ab1 | similar to alpha 5 type IV collagen isoform 1 precursor *Tribolium castaneum* | LF | extracellular matrix | 2.9 |
| 286 | zslaa0_008806 | zslaa0_008806.z1.scf | no hit | LNO | no hit | 2.9 |
| 287 | CL302 Contig1 | LF1165-2007-05-08.ab1; zslaa0_001932.z1.scf; zslaa0_014443.z1.scf; LF1090-2007-03-30.ab1; zslaa0_012466.z1.scf; LNC451-2007-12-21.ab1; L1T455-2006-12-19.ab1; L1T1002-2007-01-02.ab1; LF1285-2007-04-23.ab1; L1T413-2006-12-19.ab1; L1T1443-2007-01-24.ab1; LF558-2007-01-10.ab1; LF789-2007-03-16.ab1 | similar to LEThal family member (let2) *Tribolium castaneum* | L1T, LF, LNO, LNC | development | 2.9 |
| 288 | CL1827 Contig1 | zslaa0_002231.z1.scf; zslaa0_003041.z1.scf; zslaa0_008463.z1.scf | Major vault protein (MVP) (Lung resistance related protein). *Xenopus tropicalis* | LNO | drug resistance | 2.9 |
| 289 | zslaa0_016207 | zslaa0_016207.z1.scf | no hit | LNO | no hit | 2.9 |
| 290 | CL2731 Contig1 | LF1420-2007-04-23 .ab1; LF1820-2007-05-08.ab1 | no hit | LF | no hit | 2.9 |
| 291 | LF171 | LF171-2007-01-09.ab1 | similar to CG33653PA isoform A *Tribolium castaneum* | LF | Exocytosis | 2.9 |
| 292 | CL492 Contig1 | zslaa0_012272.z1.scf; zslaa0_018461.z1.scf; zslaa0_015502.z1.scf; HA628-2005-02-24 .ab1; LF461-2007-01-10 .ab1; LF136-2007-01-09 .ab1; LNO2324-2007-02-27 .ab1; LNO3877-2007-03-15 .ab1; PU483-2004-09-30.ab1 | similar to lethal (2) 05510 CG13432PA *Apis mellifera* | PU, LF, HA, LNO | development | 2.9 |
| 293 | NLG2275 | NLG2275-2007-09-10.ab1 | no hit | NLG | no hit | 2.9 |
| 294 | L1T_1291 | L1T_1291-2007-01-15.ab1 | Ras related protein Rab10 *Homo sapiens* (Human) | L1T | signal transduction | 2.9 |
| 295 | CL3163 Contig1 | NLG585-2007-06-04 .ab1; LNC2803-2008-01-15.ab1 | Short gastrulation *Tribolium castaneum* (Red flour beetle) | LNC, NLG | development | 2.9 |
| 296 | zslaa0_008104 | zslaa0_008104.z1.scf | Rho GTPaselike protein *Schistosoma japonicum* (Blood fluke) | LNO | signal transduction | 2.9 |
| 297 | zslaa0_009245 | zslaa0_009245.z1.scf | GATA4 transcription factor *Homo sapiens* (Human) | LNO | development | 2.9 |
| 298 | CL401 Contig1 | LF1474-2007-04-23.ab1; zslaa0_008569.z1.scf; LPU476-2007-09-05.ab1; zslaa0_015256.z1.scf; L1T_1188-2007-01-15.ab1; FN4553-2003-03-05.ab1; L1T3-2006-11-24.ab1; PU388-2004-09-28.ab1; LF1547-2007-05-08.ab1; L1T_1116-2007-01-15.ab1; LPU418-2007-09-05.ab1 | ENSANGP00000015354 *Anopheles gambiae str*. PEST | ME, L1T, PU, LF, LPU, LNO | cuticula | 2.9 |
| 299 | CL3126 Contig1 | NLG158-2007-05-14.ab1; NLG1526-2007-08-23.ab1 | no hit | NLG | no hit | 2.9 |
| 300 | CL2386 Contig1 | zslaa0_000194.z1.scf; LNO1926-2007-02-19 .ab1; LNO2736-2007-03-06.ab1 | similar to CG9967PA isoform A *Tribolium castaneum* | LNO | unknown function | 2.9 |
| 301 | LNC1339 | LNC1339-2007-12-21.ab1 | Transposase C*eratitis rosa* (Natal fruit fly) | LNC | ion transporter activity | 2.9 |
| 302 | LF535 | LF535-2007-01-10.ab1 | no hit | LF | no hit | 2.9 |
| 303 | CL59 Contig1 | LNC954-2007-12-28.ab1; zslaa0_007034.z1.scf; LPU232-2007-07-30.ab1; LF672-2007-01-11.ab1; HAV-FN-FN2267-2001-11-30.ab1; LPU415-2007-09-05.ab1; LPU1811-2007-09-05.ab1; LPU1762-2007-09-05.ab1; LPU402-2007-09-05.ab1; LPU1986-2007-09-05.ab1; zslaa0_018238.z1.scf; zslaa1_010189.z1.scf; LF1373-2007-05-08.ab1; FN4643-2003-03-06.ab1; FN4979-2003-03-12.ab1; LNC1251-2007-12-21.ab1; HAV-FN-FN2616-2001-12-13.ab1; HA314-2004-08-13.ab1; LF1892-2007-05-08.ab1; LPU1979-2007-09-05.ab1; L1T1521-2007-02-26.ab1; HAV-FN-FN2121-rerun-2001-12-03.ab1; HAV-FN-FN2121-2001-11-30.ab1; L1T1725-2007-01-24.ab1; L1T1860-2007-01-24.ab1; LNO141-2006-12-01.ab1 | no hit | ME, L1T, LF, LPU, HA, LNO, LNC | no hit | 2.9 |
| 304 | CL2344 Contig1 | 512_FN1454_F07_059 .ab1; 514_FN1454-rerun_G11_084 .ab1; FN3495-2002-03-08.ab1 | similar to sprouty related EVH1 domain containing 2 *Monodelphis domestica* | FB, ME | development | 2.9 |
| 305 | HAV-FN-FN2478 | HAV-FN-FN2478-2001-12-10.ab1 | no hit | ME | no hit | 2.8 |
| 306 | LNO612 | LNO612-2007-02-06.ab1 | Homologue of Sarcophaga 2629kDa proteinase *Periplaneta americana* (American cockroach) | LNO | proteolysis | 2.8 |
| 307 | zslaa0_000574 | zslaa0_000574.z1.scf | no hit | LNO | no hit | 2.8 |
| 308 | CL2253 Contig1 | zslaa0_008077.z1.scf; LNC491-2007-12-21.ab1; LNC3513-2009-02-10.ab1 | similar to Tramtrack protein beta isoform (Tramtrack p69) (Fushi tarazu repressor protein) *Tribolium castaneum* | LNO, LNC | development | 2.8 |
| 309 | zslaa0_014183 | zslaa0_014183.z1.scf | similar to CG8472PA isoform A *Tribolium castaneum* (Calmodulin) | LNO | mediates the control of a large number of enzymes and other proteins by Ca2+ | 2.8 |
| 310 | CL460 Contig1 | zslaa0_002304.z1.scf; CC105-rerun-2003-03-14.ab1; CC105-2003-03-13.ab1; LF1589-2007-05-08.ab1; NLG3060-2007-09-26.ab1; LF411-2007-01-10.ab1; LPU973-2007-08-29.ab1; NLG2137-2007-09-26.ab1; LF940-2007-03-16.ab1 | similar to aldehyde dehydrogenase 7 family member A1 *Tribolium castaneum* | LF, LPU, CC, LNO, NLG | Oxidoreductase | 2.8 |
| 311 | LNO3741 | LNO3741-2007-02-20.ab1 | no hit | LNO | no hit | 2.8 |
| 312 | NLG2255 | NLG2255-2007-09-10.ab1 | no hit | NLG | no hit | 2.8 |
| 313 | zslaa0_002151 | zslaa0_002151.z1.scf | Starch branching enzyme ii *Aedes aegypti* (Yellowfever mosquito) | LNO | glycolysis/ gluconeogenesis | 2.8 |
| 314 | LPU1570 | LPU1570-2007-09-04.ab1 | similar to CG9342PA *Tribolium castaneum* | LPU | phosphatidylcholine transmembrane transporter activity | 2.8 |
| 315 | LF1754 | LF1754-2007-05-08.ab1 | no hit | LF | no hit | 2.8 |
| 316 | CL579 Contig1 | LPU223-2007-07-30 .ab1; LNC1815-2008-01-14.ab1 | no hit | LPU, LNC | no hit | 2.8 |
| 317 | zslaa0_010479 | zslaa0_010479.z1.scf | GE22530 [*Drosophila yakuba*] | LNO | unknown function | 2.8 |
| 318 | CL1834 Contig1 | zslaa0_012133.z1.scf; LNO3294-2007-02-15.ab1 | similar to G1/Sspecific cyclinD3 *Apis mellifera* | LNO | signal transduction | 2.8 |
| 319 | CL2406 Contig1 | LNC3439-2009-02-09.ab1; LNC1557-2008-01-11.ab1; LPU1916-2007-09-05.ab1 | no hit | LPU, LNC | no hit | 2.8 |
| 320 | LNO2205 | LNO2205-2007-02-27.ab1 | Myosin heavy chain muscle *Drosophila melanogaster* (Fruit fly) | LNO | myosin | 2.8 |
| 321 | NLG582 | NLG582-2007-06-04.ab1 | no hit | NLG | no hit | 2.8 |
| 322 | L1T1678 | L1T1678-2007-01-12.ab1 | no hit | L1T | no hit | 2.8 |
| 323 | CL2146 Contig1 | LNO822-2007-01-03 .ab1; LNO1742-2007-02-08.ab1 | similar to H28G03.4 [*Hydra magnipapillata*] | LNO | unknown function | 2.7 |
| 324 | CL3481 Contig1 | zslaa0_006013.z1.scf; LF1848-2007-05-08.ab1 | similar to CG3057PA isoform A *Tribolium castaneum* | LF, LNO | transporter activity | 2.7 |
| 325 | zslaa0_016030 | zslaa0_016030.z1.scf | *Parhyale hawaiensis* short gastrulation protein mRNA | LNO | Growth regulation | 2.7 |
| 326 | CL1098 Contig1 | zslaa0_015189.z1.scf; zslaa0_008221.z1.scf; zslaa0_011377.z1.scf; zslaa0_015162.z1.scf; LNO1904-2007-02-19.ab1 | similar to 6phosphofructo2kinase *Ornithorhynchus anatinus* | LNO | fructose 2,6-bisphosphate metabolic process | 2.7 |
| 327 | NLG2733 | NLG2733-2007-09-17.ab1 | Isopentenyl-diphosphate Delta-isomerase 1 [*Lepeophtheirus salmonis*] | NLG | sterol biosynthesis | 2.7 |
| 328 | zslaa0_014144 | zslaa0_014144.z1.scf | no hit | LNO | no hit | 2.7 |
| 329 | CL2923 Contig1 | zslaa0_005357.z1.scf; NLG3007-2007-09-26.ab1 | similar to Longitudinals lacking protein isoform G isoform 5 *Tribolium castaneum* | LNO, NLG | differentiation | 2.7 |
| 330 | zslaa0_016284 | zslaa0_016284.z1.scf | Ribose 5phosphate isomerase A *Danio rerio* (Zebrafish) (Brachydanio rerio) | LNO | ribose-5-phosphate isomerase activity | 2.7 |
| 331 | LF1486 | LF1486-2007-04-23.ab1 | MethionineRsulfoxide reductase *Drosophila melanogaster* (Fruit fly) | LF | oxidation reduction | 2.7 |
| 332 | NLG1589 | NLG1589-2007-08-23.ab1 | Putative uncharacterized protein *Caenorhabditis elegans* | NLG | unknown function | 2.7 |
| 333 | LF429 | LF429-2007-01-10.ab1 | no hit | LF | no hit | 2.7 |
| 334 | FN4626 | FN4626-2003-03-06.ab1 | GH26463p *Drosophila melanogaster* (Fruit fly) | ME | protein phosphorylation | 2.7 |
| 335 | CL3326 Contig1 | FN5489-2005-02-10.ab1; FN3592-2002-03-25.ab1 | GTPbinding protein Rac1p Yarrowia lipolytica (*Candida lipolytica*) | ME | signal transduction | 2.7 |
| 336 | CL3176 Contig1 | LNC172-2007-11-22.ab1; zslaa0_017283.z1.scf | D-lactate dehydrogenase [*Daphnia pulex*] | LNO, LNC | aerobic respiration | 2.7 |
| 337 | CL1732 Contig1 | LNO2864-2007-03-07 .ab1; NLG2438-2007-09-14 .ab1; LF1674-2007-04-23.ab1 | no hit | LF, LNO, NLG | no hit | 2.7 |
| 338 | CL178 Contig2 | zslaa0_003353.z1.scf; HA534-2005-02-14 .ab1; zslaa0_013810.z1.scf; zslaa0_010560.z1.scf; zslaa0_016388.z1.scf; zslaa0_015044.z1.scf; LNO2226-2007-02-27 .ab1; LNO2284-2007-02-27 .ab1; FN4107-2003-02-18 .ab1; LN01226-2007-02-08 .ab1; LF1132-2007-05-08 .ab1; FN3270-2002-03-08 .ab1; PU575-2004-09-30.ab1 | Ferritin heavy chain 1 [*Lepeophtheirus salmonis*] | ME, PU, LF, HA, LNO | Iron storage | 2.7 |
| 339 | CL2065 Contig1 | zslaa0_016774.z1.scf; LNO1454-2007-02-14.ab1; L1T512-2006-12-21.ab1 | no hit | L1T, LNO | no hit | 2.7 |
| 340 | CL716 Contig1 | zslaa0_016311.z1.scf; LF1753-2007-05-08.ab1; zslaa0_011083.z1.scf; zslaa0_017111.z1.scf; LNO1501-2007-02-07.ab1; LPU1908-2007-09-05.ab1; L1T1847-2007-01-24.ab1 | similar to CG8026PB isoform B *Tribolium castaneum* | L1T, LF, LPU, LNO | transporter activity | 2.7 |
| 341 | CL1178 Contig1 | NLG2056-2007-08-03.ab1; LF1660-2007-04-23.ab1; LNO2557-2007-03-01.ab1; LPU1114-2007-08-29.ab1; FN4478-2003-03-05.ab1 | Dj1 (Mammalian transcriptional regulator) related protein 1.1 *Caenorhabditis elegans* | ME, LF, LPU, LNO, NLG | transcription regulation | 2.7 |
| 342 | CL2597 Contig1 | zslaa0_009941.z1.scf; LNC3715-2009-03-06.ab1 | no hit | LNO, LNC | no hit | 2.7 |
| 343 | CL1357 Contig1 | NLG3237-2008-12-22 .ab1; zslaa0_017289.z1.scf; FN4482-2003-03-05 .ab1; FN4988-2003-03-12.ab1 | no hit | ME, LNO, NLG | no hit | 2.7 |
| 344 | LF747 | LF747-2007-03-16.ab1 | Cofilin/actin depolymerizing factor homolog *Drosophila melanogaster* (Fruit fly) | LF | development | 2.7 |
| 345 | FN4604 | FN4604-2003-03-06.ab1 | no hit | ME | no hit | 2.7 |
| 346 | CL246 Contig1 | zslaa0_002293.z1.scf; zslaa0_013428.z1.scf; zslaa0_007617.z1.scf; LNC924-2007-12-28.ab1; LNO2134-2007-03-06.ab1; LNC3726-2009-03-06.ab1; HAV-FN-FN2535-2001-12-13.ab1; NLG2876-2007-09-18.ab1; zslaa0_014037.z1.scf; zslaa0_007236.z1.scf; HAV-FN-FN2307-2001-12-10.ab1; NLG2780-2007-09-17.ab1; 470_FN-1010_FB_T3.ab1; HA545-2005-02-14.ab1 | Oxidative stress protein *Aurelia aurita* (Moon jellyfish) | FB, ME, HA, LNO, LNC, NLG | stress response | 2.6 |
| 347 | CL3348 Contig1 | LNO3825-2007-03-15.ab1; NLG2832-2007-09-18.ab1 | CG32484PA *Drosophila melanogaster* (Fruit fly) | LNO, NLG | signal transduction | 2.6 |
| 348 | CL2416 Contig1 | zslaa0_012991.z1.scf; zslaa0_015023.z1.scf | Pyroglutamyl-peptidase 1 [*Lepeophtheirus salmonis*] | LNO | cysteine-type peptidase activity | 2.6 |
| 349 | NLG1386 | NLG1386-2007-08-22.ab1 | Innexin inx2 *Drosophila melanogaster* (Fruit fly) | NLG | development | 2.6 |
| 350 | CL730 Contig1 | zslaa0_013424.z1.scf; zslaa0_003151.z1.scf; zslaa0_005879.z1.scf; LNO957-2007-02-13.ab1; LF1457-2007-04-23.ab1; zslaa0_008662.z1.scf | Pyruvate kinase *Fugu rubripes* (Japanese pufferfish) (*Takifugu rubripes*) | LF, LNO | glycolysis/ gluconeogenesis | 2.6 |
| 351 | CL2041 Contig1 | zslaa0_012312.z1.scf; zslaa0_017139.z1.scf; LNO1579-2007-02-07.ab1 | no hit | LNO | no hit | 2.6 |
| 352 | LNC2310 | LNC2310-2008-01-15.ab1 | similar to CG18619PA isoform A *Apis mellifera* | LNC | regulation of transcription | 2.6 |
| 353 | CL3455 Contig1 | zslaa0_013476.z1.scf; zslaa0_015340.z1.scf | no hit | LNO | no hit | 2.6 |
| 354 | LNC1976 | LNC1976-2008-01-15.ab1 | no hit | LNC | no hit | 2.6 |
| 355 | CL91 Contig2 | zslaa0_007334.z1.scf; zslaa0_003268.z1.scf; zslaa0_003628.z1.scf; zslaa0_005636.z1.scf; zslaa0_009244.z1.scf; zslaa0_012254.z1.scf; zslaa0_013908.z1.scf; zslaa0_008462.z1.scf; zslaa0_017274.z1.scf; zslaa0_007384.z1.scf; zslaa0_013490.z1.scf; LNO2610-2007-03-01.ab1; LNO3268-2007-02-15.ab1 | Polyadenylatebinding protein *Aedes aegypti* (Yellowfever mosquito) | LNO | mRNA processing | 2.6 |
| 356 | FN2237 | HAV-FN-FN2237-2001-11-30.ab1 | probable D-lactate dehydrogenase, mitochondrial [*Danio rerio*] | ME | oxidation reduction | 2.6 |
| 357 | CL340 Contig1 | LNO476-2007-02-08 .ab1; LF578-2007-01-10 .ab1; LNC2081-2008-01-15.ab1 | similar to CG8663PA isoform A *Tribolium castaneum* | LF, LNO, LNC | ATP biosynthesis | 2.6 |
| 358 | zslaa0_005887 | zslaa0_005887.z1.scf | G protein a subunit 4 *Ephydatia fluviatilis* | LNO | signal transduction | 2.6 |
| 359 | CL682 Contig1 | L1T_1233-2007-01-15 .ab1; FN4272-2003-02-18 .ab1; NLG1404-2007-08-22 .ab1; PU248-2004-09-21 .ab1; FN3559-2002-03-25 .ab1; FN4272-rerun-2003-02-19 .ab1; FN4676-2003-03-06.ab1 | similar to Pyruvate kinase (PK) isoform 1 *Tribolium castaneum* | ME, L1T, PU, NLG | glycolysis/ gluconeogenesis | 2.6 |
| 360 | CL961 Contig1 | zslaa0_014759.z1.scf; zslaa0_013204.z1.scf; zslaa0_011375.z1.scf; zslaa0_010501.z1.scf; NLG1736-2007-08-23 .ab1; LNC1663-2008-01-14.ab1 | At1g14870 [*Caligus clemensi*] (Protein plant cadmium resistance 2) | LNO, LNC, NLG | Unknown function | 2.6 |
| 361 | LNO4044 | LNO4044-2007-03-09.ab1 | similar to Betahexosaminidase beta chain precursor (Nacetylbetaglucosaminidase) (BetaNacetylhexosaminidase) (Hexosaminidase B) (Cervical cancer protooncogene 7) (HCC7) *Gallus gallus* | LNO | Glycosidase | 2.6 |
| 362 | zslaa0_008243 | zslaa0_008243.z1.scf | Broad-complex core protein isoform 6 [*Lepeophtheirus salmonis*] | LNO | development | 2.6 |
| 363 | zslaa0_007519 | zslaa0_007519.z1.scf | similar to phospholysine phosphohistidine inorganic pyrophosphate phosphatase *Strongylocentrotus purpuratus* | LNO | inorganic diphosphatase activity | 2.6 |
| 364 | CL1343 Contig1 | zslaa0_012677.z1.scf; zslaa0_003595.z1.scf; zslaa0_011061.z1.scf | Guanine nucleotidebinding protein G(Q) alpha subunit *Lytechinus variegatus* (Sea urchin) | LNO | signal transduction | 2.6 |
| 365 | LNO3582 | LNO3582-2007-03-05.ab1 | similar to CG6414PA *Tribolium castaneum* | LNO | Hydrolase | 2.6 |
| 366 | zslaa0_010212 | zslaa0_010212.z1.scf | no hit | LNO | no hit | 2.6 |
| 367 | L1T540 | L1T540-2006-12-21.ab1 | no hit | L1T | no hit | 2.6 |
| 368 | CL1555 Contig1 | LF1763-2007-05-08.ab1; L1T1739-2007-01-24.ab1; L1T934-2007-01-11.ab1; HAV-FN-FN1973-2001-11-30.ab1 | 3hydroxyisobutyrylCoA hydrolase mitochondrial precursor *Xenopus tropicalis* (Western clawed frog) (*Silurana tropicalis*) | ME, L1T, LF | Amino-acid degradation | 2.6 |
| 369 | zslaa1_010152 | zslaa1_010152.z1.scf | no hit | LNO | No hit | 2.6 |
| 370 | CL1562 Contig1 | LNC270-2007-12-18.ab1; LNC404-2007-12-21.ab1; LNC1369-2007-12-21.ab1; zslaa0_016316.z1.scf | similar to Glutamatecysteine ligase catalytic subunit (Gammaglutamylcysteine synthetase) (GCS heavy chain) isoform 1 *Tribolium castaneum* | LNO, LNC | stress response | 2.6 |
| 371 | CL3247 Contig1 | LNO588-2007-02-13 .ab1; LNC1757-2008-01-24.ab1 | similar to signal transducer and activator of transcription 5.1 *Apis mellifera* | LNO, LNC | transcription activation | 2.6 |
| 372 | zslaa0_007149 | zslaa0_007149.z1.scf | Novel protein similar to vertebrate superkiller viralicidic activity 2like 2 *Danio rerio* (Zebrafish) (*Brachydanio rerio*) | LNO | helicase | 2.5 |
| 373 | CL882 Contig1 | zslaa0_012458.z1.scf; zslaa0_015570.z1.scf; zslaa0_007561.z1.scf; LNO3396-2007-02-19 .ab1; LNO3481-2007-03-05 .ab1; L1T1859-2007-01-24.ab1 | Abhydrolase domain-containing protein 10, mitochondrial [*Lepeophtheirus*  *salmonis*] | L1T, LNO | peptidase | 2.5 |
| 374 | CL1546 Contig1 | zslaa0_006213.z1.scf; zslaa0_007333.z1.scf; LF1689-2007-04-23 .ab1; 364_FN745_ME45_T3.ab1 | no hit | ME, LF, LNO | no hit | 2.5 |
| 375 | LNC1093 | LNC1093-2007-12-28.ab1 | Diphosphoinositol polyphosphate phosphohydrolase 2 [*Lepeophtheirus*  *salmonis*] | LNC | hydrolase | 2.5 |
| 376 | L1T1353 | L1T1353-2007-01-23.ab1 | no hit | L1T | no hit | 2.5 |
| 377 | NLG133 | NLG133-2007-05-14.ab1 | no hit | NLG | no hit | 2.5 |
| 378 | zslaa0_011091 | zslaa0_011091.z1.scf | no hit | LNO | no hit | 2.5 |
| 379 | CL1490 Contig1 | LNO3753-2007-02-20 .ab1; LF812-2007-02-12.ab1 | Protein tyrosine phosphatase *Bombyx mori* (Silk moth) | LF, LNO | phosphatase | 2.5 |
| 380 | CL262 Contig1 | zslaa0_012356.z1.scf; zslaa0_007345.z1.scf; zslaa0_013472.z1.scf; zslaa0_010315.z1.scf; zslaa0_015064.z1.scf; zslaa0_008295.z1.scf; zslaa0_008410.z1.scf; LNO3445-2007-03-05 .ab1; LNO3574-2007-03-05 .ab1; LF1414-2007-04-23 .ab1; LNO2915-2007-03-07 .ab1; NLG2215-2007-09-10 .ab1; LNC2345-2008-01-15 .ab1; LNC3724-2009-03-06.ab1 | Ribosephosphate pyrophosphokinase *Drosophila melanogaster* (Fruit fly) | LF, LNO, LNC, NLG | nucleoside biosynthesis | 2.5 |
| 381 | L1T_1280 | L1T_1280-2007-01-15.ab1 | similar to CG2246PB isoform B *Tribolium castaneum* | L1T | nucleoside metabolic | 2.5 |
| 382 | CL91 Contig1 | zslaa0_006826.z1.scf; LPU356-2007-08-29.ab1; zslaa0_010357.z1.scf; LNC328-2007-12-18.ab1; LNC2523-2008-01-15.ab1; zslaa0_017006.z1.scf; LF1079-2007-03-30.ab1; LNO1860-2007-02-16.ab1 | similar to poly A binding protein cytoplasmic 1 isoform 1 *Tribolium castaneum* | LF, LPU, LNO, LNC | splicing | 2.5 |
| 383 | LNO388 | LNO388-2007-01-12.ab1 | Ribose 5phosphate isomerase A *Danio rerio* (Zebrafish) (*Brachydanio rerio*) | LNO | oxidation reduction | 2.5 |
| 384 | HA320 | HA320-2004-08-13.ab1 | Mitogen activated protein kinase kinase kinase 4 mapkkk4 mekk4 *Aedes aegypti* (Yellowfever mosquito) | HA | stress response | 2.5 |
| 385 | CL1896 Contig1 | zslaa0_017016.z1.scf; zslaa0_002147.z1.scf; zslaa0_0097_E05.ab1 | similar to Ubiquitin carboxyterminal hydrolase CG4265PA *Apis mellifera* | LNO | nucleosid/ tid metabolism | 2.5 |
| 386 | CL390 Contig1 | zslaa0_010734.z1.scf; zslaa0_016753.z1.scf; zslaa0_007673.z1.scf; LNO624-2007-02-06.ab1; LNO1563-2007-02-07.ab1; LNO1548-2007-02-07.ab1; LF310-2007-01-10.ab1; L1T738-2006-12-21.ab1; 481_FN1183_C11_082.ab1; L1T583-2006-12-21.ab1 | no hit | FB, L1T, LF, LNO | no hit | 2.5 |
| 387 | zslaa0_006052 | zslaa0_006052.z1.scf | no hit | LNO | no hit | 2.5 |
| 388 | LPU210 | LPU210-2007-07-30.ab1 | facilitated trehalose transporter Tret1 [*Bombyx mori*] | LPU | Sugar transport | 2.5 |
| 389 | CL1775 Contig1 | zslaa0_012690.z1.scf; zslaa0_007731.z1.scf; NLG2231-2007-09-10.ab1 | Cationtransporting ATPase *Drosophila melanogaster* (Fruit fly) | LNO, NLG | ion transporter activity | 2.5 |
| 390 | HA575 | HA575-2005-02-14.ab1 | similar to CG7717PB isoform B *Tribolium castaneum* | HA | stress response | 2.5 |
| 391 | LF1768 | LF1768-2007-05-08.ab1 | similar to CG7433PA isoform A *Tribolium castaneum* | LF | gamma-aminobutyric acid metabolic process | 2.5 |
| 392 | CL2872 Contig1 | zslaa0_019293.z1.scf; LNC1711-2008-01-24.ab1 | no hit | LNO, LNC | no hit | 2.5 |
| 393 | zslaa0_015372 | zslaa0_015372.z1.scf | no hit | LNO | no hit | 2.5 |
| 394 | CL1583 Contig1 | zslaa0_008861.z1.scf; NLG1471-2007-08-22.ab1; L1T_1175-2007-01-15.ab1; HAV-FN-FN2401-2001-12-10.ab1 | Ribokinase [*Caligus clemensi*] | ME, L1T, LNO, NLG | Carbohydrate metabolism | 2.5 |
| 395 | zslaa0_010871 | zslaa0_010871.z1.scf | no hit | LNO | no hit | 2.5 |
| 396 | zslaa0_015724 | zslaa0_015724.z1.scf | no hit | LNO | no hit | 2.5 |
| 397 | NLG1862 | NLG1862-2007-08-23.ab1 | no hit | NLG | no hit | 2.5 |
| 398 | CL1239 Contig1 | zslaa0_011867.z1.scf; zslaa0_013125.z1.scf; LNO3961-2007-02-27 .ab1; LNO3861-2007-03-15 .ab1; LNC1608-2008-01-14.ab1 | similar to Misexpression suppressor of KSR 2 CG15669PB isoform B isoform 1 *Apis mellifera* | LNO, LNC | unknown function | 2.5 |
| 399 | CL2341 Contig1 | zslaa0_015116.z1.scf; LNO1603-2007-02-15 .ab1; zslaa0_014061.z1.scf | similar to CG5731PA *Tribolium castaneum* | LNO | metabolic | 2.5 |
| 400 | NLG1079 | NLG1079-2007-06-22.ab1 | PIWI *Aedes aegypti* (Yellowfever mosquito) | NLG | RNA-mediated gene silencing | 2.5 |
| 401 | LNC2490 | LNC2490-2008-01-16.ab1 | similar to CG32549PE isoform E *Tribolium castaneum* | LNC | nucleotide catabolism | 2.5 |
| 402 | CL934 Contig1 | zslaa0_015802.z1.scf; LNC1685-2008-01-14 .ab1; LF131-2007-01-09 .ab1; LNC2173-2008-01-15 .ab1; zslaa0_017030.z1.scf; NLG2559-2007-09-17.ab1 | Pyrroline5carboxylase synthase *Tigriopus californicus* (Marine copepod) | LF, LNO, LNC, NLG | Amino acid biosynthesis | 2.5 |
| 403 | CL1965 Contig1 | zslaa0_002815.z1.scf; zslaa0_005124.z1.scf; LNO2947-2007-03-07.ab1 | Adpribosylation factor related protein 3 *Caenorhabditis elegans* | LNO | ER-Golgi transport | 2.5 |
| 404 | CL121 Contig1 | zslaa0_000136.z1.scf; zslaa0_008146.z1.scf; zslaa0_007685.z1.scf; zslaa0_005589.z1.scf; zslaa0_016974.z1.scf; zslaa0_007581.z1.scf; zslaa0_009165.z1.scf; zslaa0_013270.z1.scf; zslaa0_014967.z1.scf; zslaa0_016627.z1.scf; zslaa0_017155.z1.scf; zslaa0_004562.z1.scf; LNO357-2007-01-12 .ab1; zslaa1_010102.z1.scf; LNO2202-2007-02-27 .ab1; LNC1311-2007-12-21 .ab1; LNO3138-2007-02-27 .ab1; LNC2962-2008-01-16 .ab1; LNC940-2007-12-28.ab1 | Heat shock protein 90 *Mamestra brassicae* (Cabbage armyworm) | LNO, LNC | stress response | 2.5 |
| 405 | zslaa0_006204 | zslaa0_006204.z1.scf | no hit | LNO | no hit | 2.5 |
| 406 | CL2668 Contig1 | LNO290-2007-01-02 .ab1; LNO493-2007-02-08.ab1 | Cytochrome P450 CYP315A1 *Manduca sexta* (Tobacco hawkmoth) (Tobacco hornworm) | LNO | neurogenesis | 2.5 |
| 407 | CL125 Contig2 | zslaa0_004293.z1.scf; zslaa0_011838.z1.scf | 60S acidic ribosomal protein P0 *Homo sapiens* (Human) | LNO | ribosomal protein | 2.5 |
| 408 | zslaa0_008426 | zslaa0_008426.z1.scf | ENSANGP00000013140 *Anopheles gambiae str.* PEST | LNO | transferase | 2.5 |
| 409 | CL1456 Contig1 | NLG1021-2007-06-22 .ab1; NLG1272-2007-06-29.ab1 | Inorganic pyrophosphatase *Aedes aegypti* (Yellowfever mosquito) | NLG | glycolysis/ gluconeogenesis | 2.5 |
| 410 | zslaa0_012996 | zslaa0_012996.z1.scf | similar to rhea CG6831PA partial *Apis mellifera* | LNO | cytoskeleton | 2.5 |
| 411 | CL2152 Contig1 | LPU912-2007-08-29 .ab1; LNC402-2007-12-21 .ab1; LPU2072-2007-09-06.ab1 | Alphaamylase *Aedes aegypti* (Yellowfever mosquito) | LPU, LNC | Carbohydrate metabolism | 2.4 |
| 412 | CL1607 Contig1 | zslaa0_011740.z1.scf; FN4538-2003-03-05.ab1; LPU605-2007-09-18.ab1 | no hit | ME, LPU, LNO | no hit | 2.4 |
| 413 | CL298 Contig1 | zslaa0_006802.z1.scf; zslaa0_006976.z1.scf; zslaa0_007241.z1.scf; zslaa0_008524.z1.scf; zslaa0_013928.z1.scf; zslaa0_001988.z1.scf; LNO3934-2007-02-27 .ab1; LF487-2007-01-10 .ab1; LNO3807-2007-03-15 .ab1; LNO1729-2007-02-08 .ab1; NLG1827-2007-08-23 .ab1; zslaa0_007007.z1.scf | Cd63prov protein *Xenopus laevis* (African clawed frog) | LF, LNO, NLG | unknown function | 2.4 |
| 414 | LNO2814 | LNO2814-2007-03-07.ab1 | XM_002426119.1 *Pediculus humanus* corporis conserved hypothetical protein | LNO | unknown function | 2.4 |
| 415 | LF1260 | LF1260-2007-04-23.ab1 | no hit | LF | no hit | 2.4 |
| 416 | CL3282 Contig1 | FN4894-rerun-2003-03-13 .ab1; FN4894-2003-03-12.ab1 | similar to CG5784PB isoform B *Tribolium castaneum* | ME | neurogenesis | 2.4 |
| 417 | CL178 Contig1 | zslaa0_016204.z1.scf; HAV-FN-FN2777-2001-12-19 .ab1; LPU1088-2007-08-31 .ab1; L1T_1286-2007-01-15.ab1 | hypothetical protein *Rattus norvegicus* | ME, L1T, LPU, LNO | oxidation reduction | 2.4 |
| 418 | CL1469 Contig1 | FN4681-2003-03-06 .ab1; LNC2544-2008-01-15 .ab1; LPU250-2007-07-30 .ab1; FN3533-2002-03-25.ab1 | similar to Lk6 CG17342PA isoform A *Apis mellifera* | ME, LPU, LNC | translation/ growth regulation | 2.4 |
| 419 | LNO595 | LNO595-2007-02-13.ab1 | no hit | LNO | no hit | 2.4 |
| 420 | CL247 Contig3 | NLG924-2007-06-21 .ab1; L1T1831-2007-01-24.ab1 | similar to CG9769PA *Apis mellifera* | L1T, NLG | translation initiation | 2.4 |
| 421 | CL247 Contig2 | zslaa0_011904.z1.scf; LNO2038-2007-02-20.ab1 | similar to CG9769PA *Apis mellifera* | LNO | translation initiation | 2.4 |
| 422 | zslaa0_007758 | zslaa0_007758.z1.scf | similar to RuvBlike 2 (p47 protein) *Apis mellifera* | LNO | helicase | 2.4 |
| 423 | CL1558 Contig1 | HAV-FN-FN2251-2001-11-30 .ab1; zslaa0_011534.z1.scf; LNO1584-2007-02-07 .ab1; LPU253-2007-07-30.ab1 | similar to AGAP007939-PA [*Tribolium castaneum*], Phosphorylase | ME, LPU, LNO | Carbohydrate metabolism | 2.4 |
| 424 | LF943 | LF943-2007-03-16.ab1 | similar to CG9148PA isoform A *Tribolium castaneum* | LF | chromatin organization | 2.4 |
| 425 | CL247 Contig4 | zslaa0_006243.z1.scf; zslaa0_018267.z1.scf; zslaa0_011104.z1.scf; zslaa0_001311.z1.scf; LNO3438-2007-03-05 .ab1; zslaa0_013370.z1.scf; zslaa1_010185.z1.scf; HAV-FN-FN2366-2001-12-10.ab1 | similar to CG9769PA *Apis mellifera* | ME, LNO | translation initiation | 2.4 |
| 426 | CL62 Contig1 | zslaa0_003327.z1.scf; zslaa0_014729.z1.scf; zslaa0_007448.z1.scf; zslaa0_014715.z1.scf; zslaa0_008611.z1.scf; zslaa0_012910.z1.scf; zslaa0_008495.z1.scf; zslaa0_010996.z1.scf; zslaa0_007590.z1.scf; zslaa0_018425.z1.scf; zslaa0_008595.z1.scf; zslaa0_017776.z1.scf; zslaa0_0097_D11.ab1; LNO2306-2007-02-27.ab1; 278_FN60_D08_062.ab1; HAV-FN-FN2342-2001-12-10.ab1; PU762-2004-12-02.ab1; FN4037-2003-02-18.ab1; LNO3748-2007-02-20.ab1; FN4561-2003-03-05.ab1; PU277-2004-09-21.ab1; FN5474-2005-02-10.ab1; L1T579-2006-12-21.ab1; 383_FN866_ME166_T3.ab1 | LP23547p *Drosophila melanogaster* (Fruit fly) | FB, ME, L1T, PU, LNO | ribosomal protein | 2.4 |
| 427 | LPU407 | LPU407-2007-09-05.ab1 | Cofilin/actindepolymerizing factor homolog *Drosophila melanogaster* (Fruit fly) | LPU | development | 2.4 |
| 428 | LNO3960 | LNO3960-2007-02-27.ab1 | 84kDa heat shock protein *Haliotis tuberculata* | LNO | stress response | 2.4 |
| 429 | CL3377 Contig1 | zslaa0_010664.z1.scf; LF1866-2007-05-08.ab1 | Calpain B *Gecarcinus lateralis* (Blackback land crab) | LF, LNO | peptidase | 2.4 |
| 430 | CL721 Contig1 | zslaa0_007223.z1.scf; LNC3319-2009-02-09 .ab1; FN3351-2002-03-08 .ab1; FN6062-2005-04-05 .ab1; HAV-FN-FN2132-2001-11-30 .ab1; NLG485-2007-06-04 .ab1; LF953-2007-03-16.ab1 | 90 kDa heat shock protein *Bemisia tabaci* (Sweetpotato whitefly) | ME, LF, LNO, LNC, NLG | stress response | 2.4 |
| 431 | CL33 Contig1 | zslaa0_017275.z1.scf; zslaa0_016429.z1.scf; 491_FN1342_B06_045.ab1; 494_FN1342-rerun_E07_051.ab1; PU272-2004-09-21.ab1; PU848-2005-03-14.ab1; LF120-2007-01-09.ab1; LF419-2007-01-10.ab1; zslaa0_011746.z1.scf; zslaa0_012065.z1.scf; LF617-2007-01-11.ab1; NLG4386-2009-03-05.ab1; LF1622-2007-04-23.ab1; LF1410-2007-04-23.ab1; zslaa0_013639.z1.scf; zslaa0_019253.z1.scf; LNO1643-2007-02-15.ab1; LF1971-2007-05-09.ab1; LNC2211-2008-01-15.ab1; FN3233-2002-03-08.ab1; 470_FN-1027_C04_037.ab1; zslaa0_016954.z1.scf; FN4522-2003-03-05.ab1; zslaa0_013677.z1.scf; LPU1218-2007-09-04.ab1; LPU1951-2007-09-05.ab1; LF1050-2007-03-30.ab1; L1T991-2007-01-11.ab1; LNC112-2007-11-22.ab1; LF716-2007-03-16.ab1; L1T_1201-2007-01-15.ab1; LNO494-2007-02-08.ab1; FN4283-2003-02-18.ab1; L1T191-2006-12-01.ab1; L1T277-2006-12-04.ab1; PU717-2004-12-02.ab1; HAV-FN-FN2320-2001-12-10.ab1 | Elongation factor 1alpha *Artemia salina* (Brine shrimp) | FB, ME, L1T, PU, LF, LPU, LNO, LNC, NLG | translation elongation | 2.4 |
| 432 | CL1522 Contig1 | LNO682-2007-02-06 .ab1; LNO488-2007-02-08 .ab1; LNO1987-2007-02-19 .ab1; LNO1916-2007-02-19.ab1 | CR936371.23, Zebrafish DNA sequence from clone DKEY-149M13 in linkage group  8 | LNO | Unknown function | 2.4 |
| 433 | CL2211 Contig1 | zslaa0_010961.z1.scf; LF879-2007-02-12.ab1; NLG4015-2009-02-17.ab1 | MGC84800 protein *Xenopus laevis* (African clawed frog) | LF, LNO, NLG | pyridoxine biosynthetic | 2.4 |
| 434 | LNO3501 | LNO3501-2007-03-05.ab1 | Glutathione Stransferase *Nilaparvata lugens* (Brown planthopper) | LNO | transferase | 2.4 |
| 435 | L1T1051 | L1T1051-2007-01-02.ab1 | similar to CG8208PB isoform B *Tribolium castaneum* | L1T | transcription regulation | 2.4 |
| 436 | PU184 | PU184-2004-09-20.ab1 | ENSANGP00000022034 *Anopheles gambiae str*. PEST | PU | transferase | 2.4 |
| 437 | NLG3104 | NLG3104-2007-09-27.ab1 | no hit | NLG | no hit | 2.4 |
| 438 | LNC1463 | LNC1463-2007-12-21.ab1 | no hit | LNC | no hit | 2.4 |
| 439 | CL107 Contig1 | zslaa0_004130.z1.scf; zslaa0_005708.z1.scf; zslaa0_010353.z1.scf; zslaa0_009838.z1.scf; zslaa0_000320.z1.scf; zslaa0_011353.z1.scf; zslaa0_013364.z1.scf; zslaa0_013907.z1.scf; zslaa0_008587.z1.scf; HA849-2005-02-25 .ab1; zslaa0_015562.z1.scf; LF477-2007-01-10 .ab1; LNO3051-2007-02-19 .ab1; LF1821-2007-05-08 .ab1; NLG451-2007-06-04 .ab1; LF158-2007-01-09 .ab1; LF888-2007-02-12 .ab1; PU888-2005-03-14 .ab1; LF267-2007-01-09 .ab1; HA224-2004-08-06 .ab1; HA289-2004-08-06.ab1 | ADPATP carrier protein *Pichia guilliermondii* (Yeast) (*Candida guilliermondii*) | PU, LF, HA, LNO, NLG | transporter activity | 2.4 |
| 440 | CL486 Contig1 | zslaa0_003520.z1.scf; zslaa0_013410.z1.scf; zslaa0_010968.z1.scf; zslaa0_011644.z1.scf; LNO3908-2007-02-27.ab1; LNO3738-2007-02-20.ab1; LN01348-2007-02-01.ab1; NLG586-2007-06-04.ab1; LNC303-2007-12-18.ab1 | 40S ribosomal protein S8 *Spodoptera frugiperda* (Fall armyworm) | LNO, LNC, NLG | ribosomal protein | 2.4 |
| 441 | CL1196 Contig1 | zslaa0_010759.z1.scf; zslaa0_013121.z1.scf; LNO1517-2007-02-07.ab1; NLG3905-2009-02-17.ab1; LNC314-2007-12-18.ab1 | similar to CG3981PB isoform B *Tribolium castaneum* | LNO, LNC, NLG | Phagocytosis, axon cargo transport | 2.4 |
| 442 | CL2136 Contig1 | LPU481-2007-09-05.ab1; LF1469-2007-04-23.ab1; LPU1376-2007-09-12.ab1 | Glucose6phosphate isomerase *Bombyx mori* (Silk moth) | LF, LPU | glycolysis/ gluconeogenesis | 2.3 |
| 443 | CL279 Contig2 | PU702-2004-12-02 .ab1; LPU2090-2007-09-06.ab1 | Homolog of *Brachydanio rerio* "Prosaposin. *Takifugu rubripes* | PU, LPU | lipid metabolism | 2.3 |
| 444 | CL567 Contig1 | zslaa0_011693.z1.scf; zslaa0_000788.z1.scf; zslaa0_009293.z1.scf; zslaa0_004238.z1.scf; zslaa0_013867.z1.scf; zslaa0_017145.z1.scf; NLG3837-2009-02-17 .ab1; LNO1467-2007-02-14.ab1 | Probable ribosome biogenesis protein RLP24. *Rattus norvegicus* | LNO, NLG | ribosomal protein | 2.3 |
| 445 | CL707 Contig1 | zslaa0_008249.z1.scf; LF618-2007-01-11.ab1; L1T1009-2007-01-02.ab1; FN3909-2003-02-11.ab1; HAV-FN-FN1948-2001-11-30.ab1; FN4847-2003-03-12.ab1 | *Aedes aegypti* ubiquitin-L40 ribosomal fusion protein (UbL40) gene | ME, L1T, LF, LNO | ribosomal protein | 2.3 |
| 446 | CL316 Contig1 | zslaa0_007974.z1.scf; zslaa0_012069.z1.scf; zslaa0_016219.z1.scf; LNO3493-2007-03-05.ab1; LF604-2007-01-11.ab1; NLG488-2007-06-04.ab1; NLG2340-2007-09-14.ab1; LNO3577-2007-03-05.ab1; LNC2296-2008-01-15.ab1; LNC2924-2008-01-16.ab1; LNC2267-2008-01-15.ab1; L1T1866-2007-01-24.ab1 | Lepeophtheirus salmonis Pacific form, Splicing factor, arginine/serine-rich 7 putative mRNA | L1T, LF, LNO, LNC, NLG | mRNA splicing | 2.3 |
| 447 | CL1103 Contig1 | 383_FN812_ME112_T3.ab1; PU361-2004-09-28.ab1; L1T1663-2007-01-12.ab1; FN6152-2005-04-05.ab1; FN3762-2003-01-16.ab1 | 60S ribosomal protein L44 *Aedes triseriatus* (Mosquito) (*Ochlerotatus triseriatus*) | ME, L1T, PU | ribosomal protein | 2.3 |
| 448 | LNC2756 | LNC2756-2008-01-16.ab1 | no hit | LNC | no hit | 2.3 |
| 449 | CL281 Contig1 | zslaa0_008906.z1.scf; zslaa0_010454.z1.scf; zslaa0_009354.z1.scf; zslaa0_003647.z1.scf; zslaa0_014482.z1.scf; zslaa0_016914.z1.scf; zslaa0_008706.z1.scf; zslaa0_012467.z1.scf; zslaa0_015331.z1.scf; LNO1624-2007-02-15.ab1; LNC832-2007-12-21.ab1; LNO3006-2007-02-19.ab1 | Elongation factor 1beta2 *Aedes aegypti* (Yellowfever mosquito) | LNO, LNC | translation elongation | 2.3 |
| 450 | CL234 Contig1 | zslaa0_018244.z1.scf; zslaa0_005155.z1.scf; zslaa0_008139.z1.scf; zslaa0_003105.z1.scf; zslaa0_015669.z1.scf; zslaa0_016444.z1.scf; zslaa0_002359.z1.scf; LNO3054-2007-02-19 .ab1; HAV-FN-FN1823-2001-11-28 .ab1; LNO3475-2007-03-05 .ab1; NLG2412-2007-09-14 .ab1; FN4049-2003-02-18.ab1 | Drosophila melanogaster lethal (2) 37Cc (l(2)37Cc), transcript variant B, mRNA | ME, LNO, NLG | development | 2.3 |
| 451 | CL179 Contig2 | zslaa0_017257.z1.scf; PU485-2004-09-30.ab1 | similar to arginine kinase *Strongylocentrotus purpuratus* | PU, LNO | ATP/ADP | 2.3 |
| 452 | CL1594 Contig1 | zslaa0_011564.z1.scf; zslaa0_003333.z1.scf; zslaa0_017794.z1.scf; LNC2040-2008-01-15.ab1 | *Lepeophtheirus salmonis* Pacific form,  repeat domain-containing protein 49 putative mRNA | LNO, LNC | Transcription activation | 2.3 |
| 453 | CL2176 Contig1 | L1T1328-2007-01-23 .ab1; NLG3617-2009-02-09 .ab1; L1T980-2007-01-11.ab1 | ORF YBR1730 *Saccharomyces cerevisiae* (Baker's yeast) | L1T, NLG | transferase | 2.3 |
| 454 | CL1850 Contig1 | zslaa0_015651.z1.scf; zslaa0_015767.z1.scf; NLG955-2007-06-21.ab1 | no hit | LNO, NLG | no hit | 2.3 |
| 455 | CL283 Contig1 | LNC2308-2008-01-15.ab1; zslaa0_000351.z1.scf; LNC382-2007-12-18.ab1; LNO3456-2007-03-05.ab1; LNO3118-2007-02-27.ab1; L1T1669-2007-01-12.ab1; L1T1454-2007-01-24.ab1; L1T1781-2007-01-24.ab1; FN4230-2003-02-18.ab1 | similar to CG31692PA isoform A *Tribolium castaneum* | ME, L1T, LNO, LNC | carbohydrate biosynthetic process | 2.3 |
| 456 | CL125 Contig1 | zslaa0_012427.z1.scf; zslaa0_016977.z1.scf; zslaa0_012714.z1.scf; zslaa0_007035.z1.scf; zslaa0_013483.z1.scf; zslaa0_009269.z1.scf; zslaa0_007390.z1.scf; LNO1873-2007-02-16 .ab1; LF1631-2007-04-23 .ab1; LF1621-2007-04-23 .ab1; LNO2076-2007-02-20 .ab1; LNO3156-2007-02-27 .ab1; HA395-2004-08-13 .ab1; PU523-2004-09-30 .ab1; HA246-2004-08-06 .ab1; PU260-2004-09-21 .ab1; HA391-2004-08-13.ab1 | *Apis mellifera* ribosomal protein LP0, transcript variant 1 (RpLP0), mRNA | PU, LF, HA, LNO | ribosomal protein | 2.3 |
| 457 | LF1065 | LF1065-2007-03-30.ab1 | Ribosomal protein S25 *Ixodes scapularis* (Blacklegged tick) (Deer tick) | LF | ribosomal protein | 2.3 |
| 458 | CL69 Contig1 | zslaa0_011688.z1.scf; zslaa0_006580.z1.scf; zslaa0_006427.z1.scf; HA544-2005-02-14.ab1; CC156-2003-03-13.ab1; LPU1776-2007-09-05.ab1; LPU2013-2007-09-06.ab1; LNO3115-2007-02-27.ab1; LNO2826-2007-03-07.ab1; LF1688-2007-04-23.ab1; LPU891-2007-08-29.ab1; LF1113-2007-05-08.ab1; LF1119-2007-05-08.ab1; HAV-FN-FN2171-2001-11-30.ab1; LNC1246-2007-12-21.ab1; HAV-FN-FN1891-2001-11-28.ab1; L1T1370-2007-01-23.ab1; zslaa0_003228.z1.scf; zslaa0_012078.z1.scf; FN5863-2005-03-14.ab1; LNC1250-2007-12-21.ab1; LNC2352-2008-01-15.ab1; LF308-2007-01-10.ab1; FN3563-2002-03-25.ab1 | *Lepeophtheirus salmonis* Pacific form, Adenosylhomocysteinase, putative mRNA | ME, L1T, LF, LPU, CC, HA, LNO, LNC | Amino acid biosynthesis | 2.3 |
| 459 | CL275 Contig1 | zslaa0_006534.z1.scf; PU450-2004-09-30 .ab1; FN5247-2005-01-19 .ab1; LF1714-2007-05-08 .ab1; CC101-2003-03-13 .ab1; CC101-rerun-2003-03-14 .ab1; LNC3296-2009-02-09 .ab1; LPU1381-2007-09-12 .ab1; HAV-FN-FN1908-2001-11-30 .ab1; FN5926-2005-03-15 .ab1; LPU313-2007-08-29 .ab1; HAV-FN-FN2727-2001-12-19 .ab1; LPU874-2007-08-29.ab1 | 60S ribosomal protein L5 *Styela clava* (Sea squirt) | ME, PU, LF, LPU, CC, LNO, LNC | ribosomal protein | 2.3 |
| 460 | CL392 Contig1 | zslaa0_007266.z1.scf; zslaa0_010243.z1.scf; zslaa0_008002.z1.scf; zslaa0_014630.z1.scf; zslaa0_002138.z1.scf; zslaa0_019260.z1.scf; NLG535-2007-06-04.ab1; NLG2235-2007-09-10.ab1; LNC3994-2009-03-05.ab1; NLG680-2007-06-13.ab1; L1T1710-2007-01-24.ab1 | no hit | L1T, LNO, LNC, NLG | no hit | 2.3 |
| 461 | CL809 Contig1 | zslaa0_002376.z1.scf; zslaa0_015711.z1.scf; zslaa0_004264.z1.scf; LNO3535-2007-03-05 .ab1; FN4479-2003-03-05 .ab1; LNC3304-2009-02-09 .ab1; LNC2322-2008-01-15.ab1 | *Lepeophtheirus salmonis* Atlantic form, Hepatic leukemia factor putative mRNA | ME, LNO, LNC | transcription | 2.3 |
| 462 | CL547 Contig1 | zslaa0_007663.z1.scf; zslaa0_015347.z1.scf; zslaa0_014635.z1.scf; 292_CC541_A06_038 .ab1; zslaa0_007349.z1.scf; NLG4460-2009-03-05 .ab1; LNO1573-2007-02-07 .ab1; LNC904-2007-12-28 .ab1; LNO495-2007-02-08.ab1 | Kinase substrate HASPP28 *Pagrus major* (Red sea bream) (Chrysophrys major) | CC, LNO, LNC, NLG | signal transduction | 2.3 |
| 463 | CL2679 Contig1 | zslaa0_002965.z1.scf; NLG1569-2007-08-23.ab1 | SD09147p *Drosophila melanogaster* (Fruit fly) | LNO, NLG | ribosomal protein | 2.3 |
| 464 | CL279 Contig1 | LNC2444-2008-01-16 .ab1; zslaa0_010029.z1.scf; zslaa0_017071.z1.scf; zslaa0_008653.z1.scf; zslaa0_006503.z1.scf; zslaa0_009396.z1.scf; L1T1814-2007-01-24 .ab1; FN3032-2002-02-18 .ab1; LNC1205-2007-12-21.ab1 | similar to prosaposin partial *Strongylocentrotus purpuratus* | ME, L1T, LNO, LNC | lipid metabolism | 2.3 |
| 465 | CL1485 Contig1 | L1T1337-2007-01-23.ab1; 383_FN871_ME171_T3.ab1; HAV-FN-FN1826-2001-11-28.ab1; FN3318-2002-03-08.ab1 | *Lepeophtheirus salmonis*, 60S ribosomal protein L39 putative mRNA | ME, L1T | ribosomal protein | 2.3 |
| 466 | L1T573 | L1T573-2006-12-21.ab1 | hypothetical protein Phum_PHUM604570 [*Pediculus humanus corporis*] | L1T | unknown function | 2.3 |
| 467 | CL756 Contig1 | LNO1792-2007-02-08.ab1; FN3252-2002-03-08.ab1; LNC1543-2008-01-11.ab1 | similar to Syndecan CG10497PA isoform A partial *Apis mellifera* | ME, LNO, LNC | differentiation | 2.3 |
| 468 | CL1740 Contig1 | HAV-FN-FN2195-2001-11-30.ab1; NLG1677-2007-08-23.ab1 | Ribosomal protein L28e *Carabus granulatus* (Ground beetle) | ME, NLG | ribosomal protein | 2.3 |
| 469 | CL1874 Contig1 | zslaa0_014177.z1.scf; zslaa0_008443.z1.scf; HAV-FN-FN2260-2001-11-30.ab1 | Thioredoxinlike protein *Maconellicoccus hirsutus* (hibiscus mealybug) | ME, LNO | electron transport | 2.3 |
| 470 | L1T205 | L1T205-2006-12-04.ab1 | no hit | L1T | no hit | 2.3 |
| 471 | CL317 Contig1 | zslaa0_003243.z1.scf; zslaa0_016659.z1.scf; zslaa0_000564.z1.scf; zslaa0_014813.z1.scf; zslaa0_000367.z1.scf; zslaa0_016834.z1.scf; zslaa0_009203.z1.scf; zslaa0_009386.z1.scf; zslaa0_016281.z1.scf; LNO2810-2007-03-07 .ab1; FN3825-2003-02-11 .ab1; LNO517-2007-02-13.ab1 | *Lepeophtheirus salmonis* Atlantic form, Ribosome biogenesis protein NSA2 homolog putative mRNA | ME, LNO | Ribosome biogenesis | 2.3 |
| 472 | zslaa0_017291 | zslaa0_017291.z1.scf | Putative uncharacterized Chaperone protein dnj16 *Caenorhabditis elegans* | LNO | stress response | 2.3 |
| 473 | zslaa0_012945 | zslaa0_012945.z1.scf | no hit | LNO | no hit | 2.3 |
| 474 | CL182 Contig2 | zslaa0_002627.z1.scf; zslaa0_010662.z1.scf; LNO1978-2007-02-19.ab1; L1T233-2006-12-04.ab1; L1T11-2006-11-24.ab1; zslaa0_013729.z1.scf | Ribosomal protein S19 *Bombyx mori* (Silk moth) | L1T, LNO | ribosomal protein | 2.3 |
| 475 | FN2662 | HAV-FN-FN2662-2001-12-13.ab1 | similar to RIKEN cDNA 2310016E02 gene [*Strongylocentrotus purpuratus*] | ME | unknown function | 2.3 |
| 476 | CL232 Contig1 | zslaa0_012680.z1.scf; FN3485-2002-03-08.ab1; HAV-FN-FN2750-2001-12-19.ab1; HAV-FN-FN2750-rerun-2001-12-20.ab1; HAV-FN-FN1935-2001-11-30.ab1; HAV-FN-FN1932-2001-11-30.ab1; HAV-FN-FN2635-2001-12-13.ab1; LPU1972-2007-09-05.ab1; L1T1877-2007-01-24.ab1; LPU878-2007-08-29.ab1; HAV-FN-FN2594-2001-12-13.ab1; FN4942-2003-03-12.ab1; HAV-FN-FN1911-2001-11-30.ab1; CC115-2003-03-13.ab1 | 40S ribosomal protein S28 *Drosophila melanogaster* (Fruit fly) | ME, L1T, LPU, CC, LNO | ribosomal protein | 2.3 |
| 477 | CL2605 Contig1 | zslaa0_005594.z1.scf; NLG3352-2009-02-09.ab1 | similar to U2 small nuclear riboprotein auxiliary factor 50 CG9998PA *Apis mellifera* | LNO, NLG | splicing | 2.3 |
| 478 | CL3763 Contig1 | zslaa0_013755.z1.scf; CC152-2003-03-13.ab1 | Ribosomal protein L35Ae *Carabus granulatus* (Ground beetle) | CC, LNO | ribosomal protein | 2.3 |
| 479 | CL3846 Contig1 | FN3895rerun-2003-02-12.ab1; FN4443-2003-03-05.ab1 | no hit | ME | no hit | 2.3 |
| 480 | zslaa0_016430 | zslaa0_016430.z1.scf | no hit | LNO | no hit | 2.3 |
| 481 | CL3704 Contig1 | zslaa0_017262.z1.scf; LPU905-2007-08-29.ab1 | 39S ribosomal protein L13, mitochondrial [*Lepeophtheirus salmonis*] | LPU, LNO | ribosomal protein | 2.3 |
| 482 | CL185 Contig2 | 40S ribosomal protein S3 [Lepeophtheirus salmonis] | 40S ribosomal protein S3 [*Lepeophtheirus salmonis*] | ME, L1T, LF | ribosomal protein | 2.3 |
| 483 | CL447 Contig1 | zslaa0_016860.z1.scf; zslaa0_002155.z1.scf; PU760-2004-12-02 .ab1; LNO3008-2007-02-19 .ab1; L1T1510-2007-02-26 .ab1; LPU936-2007-08-29 .ab1; LNC2741-2008-01-16 .ab1; FN3460-2002-03-08 .ab1; FN6080-2005-04-05 .ab1; LPU977-2007-08-29.ab1 | Ribosomal protein L23Ae *Georissus sp*. APV2005 | ME, L1T, PU, LPU, LNO, LNC | ribosomal protein | 2.3 |
| 484 | zslaa0_007462 | zslaa0_007462.z1.scf | no hit | LNO | no hit | 2.3 |
| 485 | LPU809 | LPU809-2007-08-29.ab1 | UPI0000E4E5A5 UniRef100 entry *Danio rerio* | LPU | transporter activity | 2.3 |
| 486 | LPU366 | LPU366-2007-08-29.ab1 | Nacetylgalactosaminyltransferase *Aedes aegypti* (Yellowfever mosquito) | LPU | transferase | 2.3 |
| 487 | LNC2722 | LNC2722-2008-01-16.ab1 | similar to CG33116PA *Apis mellifera* | LNC | transferase | 2.3 |
| 488 | FN4923 | FN4923-2003-03-12.ab1 | no hit | ME | no hit | 2.3 |
| 489 | CL505 Contig1 | zslaa0_010473.z1.scf; zslaa0_005501.z1.scf; zslaa0_011595.z1.scf; LNO3009-2007-02-19.ab1; FN6065-2005-04-05.ab1; 471_FN-937_FB37_T3.ab1; 364_FN757_ME57_T3.ab1; L1T6-2006-11-24.ab1 | Ribosomal protein L24e *Hister sp.* APV2005 | FB, ME, L1T, LNO | ribosomal protein | 2.3 |
| 490 | CL1021 Contig1 | 278_FN65_A09_065 .ab1; LNO660-2007-02-06 .ab1; HAV-FN-FN2737-2001-12-19 .ab1; 279_FN65rerun_A12_085 .ab1; HAV-FN-FN2877-2001-12-19.ab1 | 60S ribosomal protein L34 [*Lepeophtheirus salmonis*] | FB, ME, LNO | ribosomal protein | 2.3 |
| 491 | CL179 Contig1 | zslaa0_002988.z1.scf; PU706-2004-12-02 .ab1; zslaa0_014324.z1.scf; LF1910-2007-05-09 .ab1; HA365-2004-08-13 .ab1; LNO3560-2007-03-05 .ab1; LF1054-2007-03-30 .ab1; LPU1748-2007-09-05 .ab1; LF1842-2007-05-08 .ab1; zslaa0_013752.z1.scf; zslaa0_006923.z1.scf; zslaa0_011736.z1.scf; LPU607-2007-09-18 .ab1; LNO2220-2007-02-27.ab1 | similar to arginine kinase *Strongylocentrotus purpuratus* | PU, LF, LPU, HA, LNO | transferase | 2.3 |
| 492 | LNO1018 | LNO1018-2007-02-01.ab1 | Cell death regulatory protein GRIM19 *Bombyx mori* (Silk moth) | LNO | apoptosis | 2.3 |
| 493 | CL1262 Contig1 | PU489-2004-09-30.ab1; HAV-FN-FN2324-2001-12-10.ab1; FN4637-2003-03-06.ab1; FN4162-2003-02-18.ab1 | Ribosomal protein L38e *Georissus sp.* APV2005 | ME, PU | ribosomal protein | 2.3 |
| 494 | CL468 Contig1 | PU103-2004-09-20.ab1; LF1983-2007-05-09.ab1; LNC2256-2008-01-15.ab1; FN4077-2003-02-18.ab1; FN5807-2005-03-14.ab1; L1T707-2006-12-21.ab1; L1T_1196-2007-01-15.ab1; FN3439-2002-03-08.ab1; FN3596-2002-03-25.ab1; FN6114-2005-04-05.ab1 | 60S ribosomal protein L23 [*Caligus clemensi*] | ME, L1T, PU, LF, LNC | ribosomal protein | 2.3 |
| 495 | CL321 Contig1 | FN6090-2005-04-05 .ab1; FN4256-2003-02-18 .ab1; FN4256-rerun-2003-02-19 .ab1; L1T_1174-2007-01-15 .ab1; CC165-2003-03-13 .ab1; FN3091-2002-02-18 .ab1; HAV-FN-FN2871-2001-12-19 .ab1; LF1658-2007-04-23 .ab1; FN5187-2005-01-17 .ab1; LNO839-2007-01-03 .ab1; FN5143-2005-01-17 .ab1; L1T127-2006-12-01.ab1 | 60S ribosomal protein L31 [*Lepeophtheirus salmonis*] | ME, L1T, LFCC, LNO | ribosomal protein | 2.3 |
| 496 | L1T1722 | L1T1722-2007-01-24.ab1 | similar to prefoldin 5 isoform 1 *Tribolium castaneum* | L1T | transcription regulation | 2.3 |
| 497 | CL194 Contig1 | zslaa0_003030.z1.scf; PU831-2005-03-14.ab1; CC192-2003-03-13.ab1; LNO2543-2007-03-01.ab1; HAV-FN-FN2547-2001-12-13.ab1; 278_FN2_B01_009.ab1; FN3358-2002-03-08.ab1; FN4635-2003-03-06.ab1; HAV-FN-FN2562-2001-12-13.ab1; FN4709-2003-03-06.ab1; HA315-2004-08-13.ab1; HAV-FN-FN2660-2001-12-13.ab1; FN4628-2003-03-06.ab1 | Ribosomal protein L17 *Ixodes scapularis* (Blacklegged tick) (Deer tick) | FB, ME, PU, CC, HA, LNO | ribosomal protein | 2.3 |
| 498 | CL2204 Contig1 | LNC2216-2008-01-15 .ab1; NLG3046-2007-09-26 .ab1; NLG3960-2009-02-17.ab1 | similar to Probable nucleolar GTPbinding protein 1 isoform 1 *Apis mellifera* | LNC, NLG | negative regulator of cell proliferation | 2.3 |
| 499 | CL24 Contig1 | zslaa0_015946.z1.scf; zslaa0_015462.z1.scf; zslaa0_003584.z1.scf; zslaa0_009104.z1.scf; zslaa0_007422.z1.scf; zslaa0_011352.z1.scf; zslaa0_016569.z1.scf; zslaa0_013038.z1.scf; zslaa0_002858.z1.scf; zslaa0_007372.z1.scf; zslaa0_016412.z1.scf; zslaa0_015787.z1.scf; zslaa0_012657.z1.scf; zslaa0_016722.z1.scf; LNO251-2007-01-02 .ab1; zslaa0_010739.z1.scf; LPU1386-2007-09-12 .ab1; LNO451-2007-02-08 .ab1; LN01314-2007-02-01 .ab1; LF1304-2007-05-08 .ab1; LNO2769-2007-03-06 .ab1; FN4219-2003-02-18 .ab1; LF875-2007-02-12 .ab1; FN4260-rerun-2003-02-19 .ab1; 471_FN-964_FB64_T3 .ab1; FN4713-2003-03-06 .ab1; FN3344-2002-03-08 .ab1; LPU1925-2007-09-05 .ab1; LNC3245-2009-02-09 .ab1; zslaa0_011652.z1.scf; FN3773-2003-01-16 .ab1; LNO2701-2007-03-06 .ab1; LF993-2007-03-16 .ab1; PU394-2004-09-28 .ab1; FN4340-2003-02-19 .ab1; LNC2184-2008-01-15 .ab1; PU350-2004-09-28 .ab1; NLG558-2007-06-04 .ab1; PU352-2004-09-28 .ab1; FN3505-2002-03-25 .ab1; FN4012-2003-02-18.ab1 | Receptor for activated protein kinase C like *Blattella germanica* (German cockroach) | FB, ME, PU, LF, LPU, LNO, LNC, NLG | Signal transduction | 2.3 |
| 500 | CL186 Contig1 | zslaa0_016573.z1.scf; FN5014-2004-01-23 .ab1; zslaa1_010130.z1.scf; L1T_1248-2007-01-15 .ab1; LPU1985-2007-09-05 .ab1; HA286-2004-08-06 .ab1; FN5344-2005-01-26 .ab1; LPU342-2007-08-29 .ab1; PU761-2004-12-02 .ab1; 383_FN887_ME187_T3 .ab1; FN6137-2005-04-05 .ab1; HAV-FN-FN2706-2001-12-19 .ab1; HAV-FN-FN2333-2001-12-10 .ab1; FN5920-2005-03-15 .ab1; PU268-2004-09-21 .ab1; FN4732-2003-03-06.ab1 | 60S ribosomal protein L27a [*Lepeophtheirus salmonis]* | ME, L1T, PU, LPU, HA, LNO | ribosomal protein | 2.3 |
| 501 | CL1241 Contig1 | zslaa0_002102.z1.scf; zslaa0_016536.z1.scf; LNO3886-2007-03-15.ab1; L1T1315-2007-01-23.ab1; LNC3212-2009-02-09.ab1 | Cold shock domain-containing protein C2 [*Lepeophtheirus salmonis*] | L1T, LNO, LNC | mRNA processing | 2.3 |
| 502 | CL1404 Contig1 | zslaa0_016619.z1.scf; LNO1542-2007-02-07 .ab1; HAV-FN-FN2792-2001-12-19 .ab1; NLG1552-2007-08-23.ab1 | ADPribosylation factorlike protein 8B *Homo sapiens* (Human) | ME, LNO, NLG | signal transduction | 2.3 |
| 503 | CL3795 Contig1 | HA938-2005-03-02.ab1; LNC1869-2008-01-14.ab1 | Small GTPbinding protein Rab10 *Bombyx mori* (Silk moth) | HA, LNC | signal transduction | 2.3 |
| 504 | CL185 Contig1 | FN4766-2003-03-06.ab1; L1T1818-2007-01-24.ab1 | 40S ribosomal protein S3 [*Lepeophtheirus salmonis*] | ME, L1T | ribosomal protein | 2.3 |
| 505 | NLG1555 | NLG1555-2007-08-23.ab1 | Putative mitochondrial processing peptidase *Ostreococcus tauri* | NLG | ribosomal protein | 2.3 |
| 506 | CL1075 Contig1 | zslaa0_007982.z1.scf; zslaa0_015335.z1.scf; LNC2860-2008-01-15.ab1; LN01227-2007-02-08.ab1 | similar to Brix domaincontaining protein 2 (Ribosome biogenesis protein Brix) *Tribolium castaneum* | LNO, LNC | Required for biogenesis of the 60S ribosomal subunit | 2.3 |
| 507 | LNC2811 | LNC2811-2008-01-15.ab1 | similar to CG7638PA *Tribolium castaneum* | LNC | unknown function | 2.3 |
| 508 | LNC2655 | LNC2655-2008-01-15.ab1 | *Tribolium castaneum* similar to CG10889 CG10889-PA | LNC | nucleic acid binding | 2.3 |
| 509 | CL1137 Contig1 | zslaa0_006964.z1.scf; zslaa0_013718.z1.scf; LF456-2007-01-10 .ab1; NLG3695-2009-02-09 .ab1; LF822-2007-02-12.ab1 | Hsc70-interacting protein [*Lepeophtheirus salmonis*] | LF, LNO, NLG | stress response | 2.3 |
| 510 | CL127 Contig1 | zslaa0_010633.z1.scf; zslaa0_014072.z1.scf; HAV-FN-FN2757-rerun-2001-12-20.ab1; HA343-2004-08-13.ab1; LPU1527-2007-09-04.ab1; HA250-2004-08-06.ab1; LPU616-2007-09-18.ab1; PU227-2004-09-21.ab1; HAV-FN-FN1870-2001-11-28.ab1; HAV-FN-FN2757-2001-12-19.ab1; FN3725-2003-01-16.ab1; FN5180-2005-01-17.ab1; LNO3168-2007-02-27.ab1; PU314-2004-09-28.ab1; HA127-2004-07-28.ab1; HAV-FN-FN1824-2001-11-28.ab1 | S14e ribosomal protein *Dascillus cervinus* | ME, PU, LPU, HA, LNO | ribosomal protein | 2.3 |
| 511 | CL193 Contig1 | zslaa0_003752.z1.scf; zslaa0_003381.z1.scf; zslaa0_009161.z1.scf; zslaa0_005182.z1.scf; zslaa0_010302.z1.scf; HA620-2005-02-24 .ab1; zslaa0_008717.z1.scf; LNO2646-2007-03-01 .ab1; PU539-2004-09-30 .ab1; zslaa0_017252.z1.scf; LF335-2007-01-10 .ab1; LPU384-2007-08-29 .ab1; PU182-2004-09-20 .ab1; PU870-2005-03-14 .ab1; HAV-FN-FN2712-2001-12-19 .ab1; HAV-FN-FN2712-rerun-2001-12-20.ab1 | similar to Ribosomal protein L4 CG5502PA isoform 1 *Apis mellifera* | ME, PU, LF, LPU, HA, LNO | ribosomal protein | 2.2 |
| 512 | CL1 Contig21 | zslaa0_016428.z1.scf; zslaa0_007638.z1.scf; LF534-2007-01-10 .ab1; zslaa0_011960.z1.scf; zslaa0_015361.z1.scf; zslaa0_009274.z1.scf; HAV-FN-FN1890-2001-11-28 .ab1; L1T537-2006-12-21 .ab1; LNO3682-2007-03-09 .ab1; 491_FN1334_B05_041 .ab1; LPU1308-2007-09-12 .ab1; HAV-FN-FN2465-2001-12-10 .ab1; LPU1148-2007-08-29 .ab1; PU750-2004-12-02 .ab1; LF1686-2007-04-23 .ab1; FN4389-2003-02-19 .ab1; HAV-FN-FN2276-2001-11-30 .ab1; HAV-FN-FN2671-2001-12-13 .ab1; L1T1356-2007-01-23 .ab1; L1T470-2006-12-19 .ab1; L1T558-2006-12-21 .ab1; L1T_1137-2007-01-15 .ab1; 383_FN869_ME169_T3 .ab1; FN3982-2003-02-11 .ab1; FN4202-2003-02-18.ab1 | *Lepeophtheirus salmonis* Pacific form, 60S ribosomal protein L13A putative mRNA, complete cds | FB, ME, L1T, PU, LF, LPU, LNO | ribosomal protein | 2.2 |
| 513 | CL1367 Contig1 | zslaa0_003025.z1.scf; zslaa0_005210.z1.scf; 383_FN832_ME132_T3.ab1; LNO3341-2007-02-19.ab1 | Ribosomal protein L22e *Sphaerius sp*. APV2005 | ME, LNO | ribosomal protein | 2.2 |
| 514 | CL161 Contig1 | zslaa0_006984.z1.scf; zslaa0_004120.z1.scf; zslaa0_003336.z1.scf; zslaa0_013857.z1.scf; zslaa0_003612.z1.scf; zslaa0_011503.z1.scf; zslaa0_013631.z1.scf; zslaa0_016725.z1.scf; zslaa0_013523.z1.scf; zslaa0_016662.z1.scf; LNO1934-2007-02-19.ab1; LPU1022-2007-08-31.ab1; LNO1881-2007-02-16.ab1; LNC2992-2008-01-16.ab1; 278_FN92_D12_094.ab1; LNO1030-2007-02-01.ab1; LN01327-2007-02-01.ab1; FN3871-2003-02-11.ab1 | Elongation factor 1-gamma [*Lepeophtheirus salmonis*] | FB, ME, LPU, LNO, LNC | translation elongation/ apoptosis | 2.2 |
| 515 | CL2809 Contig1 | zslaa0_015117.z1.scf; LNO1966-2007-02-19.ab1 | similar to Developmental embryonic B CG16792PA *Apis mellifera* | LNO | ion transporter activity | 2.2 |
| 516 | CL239 Contig1 | zslaa0_018460.z1.scf; zslaa0_008640.z1.scf; zslaa0_014416.z1.scf; zslaa0_009620.z1.scf; zslaa0_003990.z1.scf; zslaa0_009313.z1.scf; zslaa0_017114.z1.scf; zslaa0_009644.z1.scf; NLG1713-2007-08-23.ab1; NLG1012-2007-06-22.ab1; LNC3215-2009-02-09.ab1; HA734-2005-02-25.ab1; L1T1873-2007-01-24.ab1; LNC2214-2008-01-15.ab1 | similar to CG8636PA *Apis mellifera* | L1T, HA, LNO, LNC, NLG | translation initiation | 2.2 |
| 517 | CL2250 Contig1 | zslaa0_004109.z1.scf; FN5220-2005-01-19.ab1; NLG453-2007-06-04.ab1 | Wdrepeat protein *Aedes aegypti* (Yellowfever mosquito) | ME, LNO, NLG | rRNA processing | 2.2 |
| 518 | LPU1969 | LPU1969-2007-09-05.ab1 | S5e ribosomal protein *Timarcha balearica* | LPU | ribosomal protein | 2.2 |
| 519 | CL201 Contig1 | zslaa0_006957.z1.scf; zslaa0_013021.z1.scf; zslaa0_003080.z1.scf; zslaa0_012724.z1.scf; zslaa0_008066.z1.scf; zslaa0_012074.z1.scf; zslaa0_015534.z1.scf; zslaa0_000860.z1.scf; zslaa0_014471.z1.scf; LNO1092-2007-02-01.ab1; LNO503-2007-02-13.ab1; NLG4136-2009-02-24.ab1; 542_FN1789_A12_056.ab1; LNO3721-2007-02-20.ab1; LPU821-2007-08-29.ab1 | similar to CG10664PA isoform A *Tribolium castaneum* | ME, LPU, LNO, NLG | cytochrome c oxidase | 2.2 |
| 520 | CL993 Contig1 | LNO3792-2007-02-20.ab1; L1T1-2006-11-24.ab1; LF1150-2007-05-08.ab1; CC189-2003-03-13.ab1; LPU1754-2007-09-05.ab1 | 60S ribosomal protein L27 *Suberites domuncula* (Sponge) | L1T, LF, LPU, CC, LNO | ribosomal protein | 2.2 |
| 521 | CL216 Contig1 | 278_FN75_C10_070 .ab1; 279_FN75rerun_E12_087 .ab1; zslaa0_017060.z1.scf; zslaa0_006861.z1.scf; zslaa0_015051.z1.scf; NLG1707-2007-08-23 .ab1; NLG1359-2007-08-22 .ab1; LNO3267-2007-02-15 .ab1; FN3059-2002-02-18 .ab1; HAV-FN-FN2689-2001-12-13 .ab1; L1T1071-2007-01-02 .ab1; LF1827-2007-05-08 .ab1; PU445-2004-09-30 .ab1; HAV-FN-FN2835-2001-12-19 .ab1; L1T931-2007-01-11.ab1 | *Lepeophtheirus salmonis* Pacific form,40S ribosomal protein S18 putative mRNA, complete cds | FB, ME, L1T, PU, LF, LNO, NLG | ribosomal protein | 2.2 |
| 522 | CL715 Contig2 | LPU951-2007-08-29.ab1; HA551-2005-02-14.ab1 | similar to CG3186PA isoform A *Tribolium castaneum* | LPU, HA | translation initiation | 2.2 |
| 523 | CL174 Contig1 | zslaa0_001312.z1.scf; zslaa0_005655.z1.scf; zslaa0_013507.z1.scf; HA819-2005-02-25 .ab1; HA748-2005-02-25 .ab1; LIT331-2006-12-18 .ab1; LF1335-2007-05-08 .ab1; LF5-2006-12-08 .ab1; LPU838-2007-08-29 .ab1; LF877-2007-02-12 .ab1; LPU213-2007-07-30 .ab1; LNO4014-2007-03-09 .ab1; LNO1525-2007-02-07 .ab1; LPU459-2007-09-05 .ab1; LNO3188-2007-02-27 .ab1; L1T757-2006-12-21 .ab1; PU128-2004-09-20.ab1 | *Lepeophtheirus salmonis* Pacific, 60S  ribosomal protein L8 putative mRNA, complete cds | L1T, PU, LF, LPU, HA, LNO | ribosomal protein | 2.2 |
| 524 | CL1816 Contig1 | HA744-2005-02-25 .ab1; HA940-2005-03-02 .ab1; LPU1875-2007-09-05.ab1 | SNB1 *Caenorhabditis elegans* | LPU, HA | signalling | 2.2 |
| 525 | CL1892 Contig1 | zslaa0_008578.z1.scf; LF1812-2007-05-08.ab1; LF272-2007-01-09.ab1 | Eukaryotic translation initiation factor 3 subunit 6 *Homo sapiens* (Human) | LF, LNO | translation initiation | 2.2 |
| 526 | LNO3624 | LNO3624-2007-03-09.ab1 | similar to chloride channel *Monodelphis domestica* | LNO | transporter activity | 2.2 |
| 527 | CL182 Contig1 | FN4509-2003-03-05.ab1; 383_FN858_ME158_T3.ab1; HAV-FN-FN1902-2001-11-28.ab1; HAV-FN-FN2331-2001-12-10.ab1; FN4773-2003-03-06.ab1; FN4291-rerun-2003-02-19.ab1; 520_FN1680_H10_073.ab1; FN4773rerun-2003-03-07.ab1; FN6117-2005-04-05.ab1 | *Lepeophtheirus salmonis* Atlantic, 40S ribosomal protein S19 putative mRNA, complete cds | FB, ME | ribosomal protein | 2.2 |
| 528 | CL574 Contig1 | zslaa0_011042.z1.scf; zslaa0_003037.z1.scf; zslaa0_005884.z1.scf; FN3780-2003-01-16 .ab1; HAV-FN-FN2363-2001-12-10 .ab1; L1T1438-2007-01-24 .ab1; 278_FN96_H12_096 .ab1; PU591-2004-09-30.ab1 | Ribosomal protein S16 *Lysiphlebus testaceipes* (Greenbugs aphid parastoid) | FB, ME, L1T, PU, LNO | ribosomal protein | 2.2 |
| 529 | CL128 Contig1 | zslaa0_002628.z1.scf; 481_FN1113_E02_007 .ab1; zslaa0_009616.z1.scf; zslaa0_008876.z1.scf; zslaa0_011763.z1.scf; zslaa0_009622.z1.scf; zslaa0_008576.z1.scf; zslaa0_017154.z1.scf; zslaa0_012934.z1.scf; LNO2856-2007-03-07 .ab1; 484_FN1113-rerun_B05_041 .ab1; PU249-2004-09-21 .ab1; CC131-2003-03-13 .ab1; LF279-2007-01-09 .ab1; LF1244-2007-04-23 .ab1; FN3980-2003-02-11 .ab1; L1T1496-2007-01-24 .ab1; L1T794-2006-12-21 .ab1; LNC3870-2009-03-06.ab1 | 40S ribosomal protein S4 [*Lepeophtheirus salmonis*] | FB, ME, L1T, PU, LFCC, LNO, LNC | ribosomal protein | 2.2 |
| 530 | FN4612 | FN4612-2003-03-06.ab1 | no hit | ME | no hit | 2.2 |

# Supplementary table 3. All down-regulated clusters/singletons sorted after strength of regulation

| **no** | **Cluster,singelt.** | **composed of EST (name)** | **Hit UniRef/ GenBank** | **library** | **categorization** | **times down-regu-lated** |
| --- | --- | --- | --- | --- | --- | --- |
| 1 | zslaa0_008831 | zslaa0_008831.z1.scf | Delta5 fatty acid desaturase A [*Caligus rogercresseyi*] | LNO | fatty acid metabolism | -1.43 E+121 |
| 2 | CL1 Contig20, FN4318, FN486, FN4653 | zslaa0_013488.z1.scf; LNO916-2007-02-13.ab1; FN3296-2002-03-08.ab1; 519_FN1517_A03_002.ab1; FN3556-2002-03-25.ab1; FN4613-2003-03-06.ab1; HAV-FN-FN2240-2001-11-30.ab1; FN4860-2003-03-12.ab1; FN3701-2003-01-16.ab1; FN4806-2003-03-12.ab1; HAV-FN-FN2364-2001-12-10.ab1; HAV-FN-FN2439-2001-12-10.ab1; FN3805-2003-02-11.ab1; FN4262-2003-02-18.ab1; FN5854-2005-03-14.ab1; FN4084-2003-02-18.ab1; FN3531-2002-03-25.ab1; FN4947-2003-03-12.ab1; 356_FN550_SB19_T7.ab1; FN4703-2003-03-06.ab1; zslaa0_014693.z1.scf; FN3779-2003-01-16.ab1; LNO2129-2007-03-06.ab1; 356_FN517_SB3_T7.ab1; 356_FN520_SB39_T7.ab1; 345_FN467_SD7_T7.ab1; HAV-FN-FN1858-2001-11-28.ab1; zslaa0_017719.z1.scf; zslaa0_012846.z1.scf; 356_FN555_SB80_T7.ab1; 356_FN583_SB35_T7.ab1; FN4910-2003-03-12.ab1; HAV-FN-FN2286-2001-11-30.ab1; FN6105-2005-04-05.ab1; FN3407-2002-03-08.ab1; FN5938-2005-03-15.ab1; 356_FN565_SB9_T7.ab1; FN3646-2002-09-18.ab1; FN3692-2002-09-18.ab1; 337_FN429_sd1_T7.ab1; 356_FN584_SB47_T7.ab1; 356_FN553_SB55_T7.ab1; 365_FN603_SD7_T3.ab1; FN3609-2002-09-18.ab1; FN3636-2002-09-18.ab1; FN3778-2003-01-16.ab1; FN4091-2003-02-18.ab1; FN4456-2003-03-05.ab1; 364_FN765_ME65_T3.ab1; FN4188-2003-02-18.ab1; FN6034-2005-04-05.ab1; FN4991-2003-03-12.ab1; FN3516-2002-03-25.ab1; FN4309-2003-02-19.ab1; FN5191-2005-01-17.ab1; HAV-FN-FN2496-2001-12-10.ab1; FN3250-rerun-2002-03-12.ab1; FN3327-2002-03-08.ab1; FN3508-2002-03-25.ab1; FN4859-2003-03-12.ab1; FN3092-2002-02-18.ab1; HAV-FN-FN2187-2001-11-30.ab1; HAV-FN-FN1830-2001-11-28.ab1; 364_FN758_ME58_T3.ab1; FN4931-2003-03-12.ab1; FN3057-2002-02-18.ab1; FN4711-2003-03-06.ab1; FN4424-2003-03-05.ab1; 383_FN845_ME145_T3.ab1; FN4276-rerun-2003-02-19.ab1; FN4838-2003-03-12.ab1; FN4276-2003-02-18.ab1; FN4330-2003-02-19.ab1; HAV-FN-FN2893-2001-12-19.ab1; HAV-FN-FN2895-2001-12-19.ab1; FN3471-2002-03-08.ab1; 542_FN1783_C11_053.ab1; HAV-FN-FN2814-2001-12-19.ab1; FN5139-2005-01-17.ab1; HAV-FN-FN2218-2001-11-30.ab1; FN5188-2005-01-17.ab1; FN3090-2002-02-18.ab1; HAV-FN-FN2518-2001-12-13.ab1; FN4328-2003-02-19.ab1; FN6188-2005-04-05.ab1; FN4771-2003-03-06.ab1; FN4771rerun-2003-03-07.ab1; HAV-FN-FN2445-2001-12-10.ab1; HAV-FN-FN2185-2001-11-30.ab1; HAV-FN-FN2394-2001-12-10.ab1; HAV-FN-FN2147-2001-11-30.ab1; 383_FN861_ME161_T3.ab1; FN4685-2003-03-06.ab1; FN3045-2002-02-18.ab1; HAV-FN-FN2294-2001-11-30.ab1; FN3845-2003-02-11.ab1; HAV-FN-FN2372-2001-12-10.ab1; FN4562-2003-03-05.ab1; FN4408-2003-03-05.ab1; FN3370-2002-03-08.ab1; FN3514-2002-03-25.ab1; FN3984-2003-02-11.ab1; FN3918-2003-02-11.ab1; FN4001-2003-02-18.ab1; FN4514-2003-03-05.ab1; FN3642-2002-09-18.ab1; HAV-FN-FN2124-2001-11-30.ab1; 356_FN536_SB41_T7.ab1; HAV-FN-FN2448-2001-12-10.ab1; FN3904-2003-02-11.ab1; FN3034-2002-02-18.ab1; HAV-FN-FN1832-2001-11-28.ab1; FN4953-2003-03-12.ab1; HAV-FN-FN2232-2001-11-30.ab1; FN3455-2002-03-08.ab1; FN4284-2003-02-18.ab1; FN4318-2003-02-19.ab1; 354_FN486_SD18_T7.ab1; FN4653-2003-03-06.ab1 | LsYAP [*Lepeophtheirus salmonis*] | SB, FB, ME, LNO | Egg york protein | -1.50 E+26 |
| 3 | CL2940 Contig1 | zslaa0_015084.z1.scf; zslaa0_015386.z1.scf | similar to CG5452-PA - *Tribolium castaneum*; Deoxynucleoside kinase | LNO | nucleobase, nucleoside, nucleotide and nucleic acid metabolic process | -7.74 E+14 |
| 4 | CL558 Contig1/2 | LNO529-2007-02-13.ab1; LNO554-2007-02-13.ab1; zslaa0_002124.z1.scf; zslaa0_008032.z1.scf; zslaa0_010331.z1.scf; zslaa0_001936.z1.scf; LNO578-2007-02-13.ab1 | Elongation of very long chain fatty acids protein AAEL008004 [*Caligus clemensi*] | LNO | fatty acid metabolism | -1.35 E+13 |
| 5 | LNO3524 | LNO3524-2007-03-05.ab1 | GA11357-PA - *Drosophila pseudoobscura* (Fruit fly) | LNO | cuticle | -6.31 E+11 |
| 6 | zslaa0_009321 | zslaa0_009321.z1.scf | Transmembrane protein nessy [*Caligus clemensi*] | LNO | germ cell development | -1.09 E+09 |
| 7 | LNO2454 | LNO2454-2007-02-14.ab1 | no hit | LNO | no hit | -4.38 E+08 |
| 8 | FN4981 | FN4981-2003-03-12.ab1 | Transmembrane protein nessy [*Caligus clemensi*] | ME | germ cell development | -3.17 E+08 |
| 9 | CL1124 Contig1 | zslaa0_005416.z1.scf; zslaa0_012292.z1.scf; zslaa0_016878.z1.scf; zslaa0_002121.z1.scf; zslaa0_012109.z1.scf | GA12987-PA - *Drosophila pseudoobscura* (Fruit fly) | LNO | involved in steroidogenesis | -1.20 E+08 |
| 10 | FN1807 | HAV-FN-FN1807-2001-11-28.ab1 | no hit | ME | no hit | -2.41 E+07 |
| 11 | zslaa0_008224 | zslaa0_008224.z1.scf | similar to Carboxypeptidase vitellogenic-like - *Monodelphis domestica* | LNO | embryonal development | -3.77 E+06 |
| 12 | CL3121 Contig1 | LNC254-2007-12-18.ab1; LNC1582-2008-01-11.ab1 | no hit | LNC | no hit | -345.1 |
| 13 | CL2068 Contig1 | zslaa0_018419.z1.scf; LPU1036-2007-08-31.ab1; HAV-FN-FN2679-2001-12-13.ab1 | no hit | ME, LPU, LNO | no hit | -157.4 |
| 14 | LNO3037 | LNO3037-2007-02-19.ab1 | Novel KRAB box containing protein - *Mus musculus* (Mouse) | LNO | transcription regulation | -108.6 |
| 15 | zslaa0_013659 | zslaa0_013659.z1.scf | GA19517-PA - *Drosophila pseudoobscura* (Fruit fly) | LNO | organic cation transmembrane transporter activity | -87.9 |
| 16 | CL3701 Contig1 | LNO3920-2007-02-27.ab1; LNC2772-2008-01-16.ab1 | ATP-binding cassette, sub-family D, member 4-like [*Saccoglossus kowalevskii*] | LNO, LNC | transporter | -45.3 |
| 17 | CL202 Contig1 | zslaa0_005506.z1.scf; zslaa0_002979.z1.scf; LNO725-2007-01-25.ab1; HA961-2005-03-02.ab1; zslaa0_016058.z1.scf; LF656-2007-01-11.ab1; LNO3685-2007-03-09.ab1; L1T1351-2007-01-23.ab1; FN5846-2005-03-14.ab1; FN3936-2003-02-11.ab1; zslaa0_014154.z1.scf; LF1630-2007-04-23.ab1 | no hit | ME, L1T, LF, HA, LNO | no hit | -42.8 |
| 18 | CL2325 Contig1 | LNC174-2007-11-22.ab1; LNC841-2007-12-21.ab1; LNO3790-2007-02-20.ab1 | Succinyl-CoA:3-ketoacid-coenzyme A transferase 1 mitochondrial precursor - *Homo sapiens* (Human) | LNO, LNC | ketone body catabolism | -29.9 |
| 19 | CL138 Contig1 | zslaa0_005445.z1.scf; zslaa0_015903.z1.scf; zslaa0_004240.z1.scf; zslaa0_005544.z1.scf; zslaa0_012236.z1.scf; zslaa0_008588.z1.scf; zslaa0_017140.z1.scf; zslaa0_010026.z1.scf; zslaa0_006023.z1.scf; LNO3231-2007-02-15.ab1; HAV-FN-FN1805-2001-11-28.ab1; FN4159-2003-02-18.ab1; FN4159-rerun-2003-02-19.ab1; L1T1715-2007-01-24.ab1; L1T_1134-2007-01-15.ab1; zslaa0_011115.z1.scf; LF192-2007-01-09.ab1; LF539-2007-01-10.ab1; L1T_1114-2007-01-15.ab1 | >gnl|BL_ORD_ID|1219832 hypothetical protein BRAFLDRAFT_125500 [*Branchiostoma floridae*] | ME, L1T, LF, LNO | unknown function | -29.1 |
| 20 | zslaa0_012168 | zslaa0_012168.z1.scf | no hit | LNO | no hit | -29.0 |
| 21 | zslaa0_009218 | zslaa0_009218.z1.scf | Chromosome 15 SCAF14542 whole genome shotgun sequence - *Tetraodon nigroviridis* (Green puffer) | LNO | oxidoreductase activity | -28.3 |
| 22 | zslaa0_015691 | zslaa0_015691.z1.scf | similar to acid alpha glucosidase [*Strongylocentrotus purpuratus*] | LNO | carbohydrate metabolism | -25.3 |
| 23 | zslaa0_003651 | zslaa0_003651.z1.scf | pro-sucrase-isomaltase (EC 3.2.1.48-10) [*Oryctolagus cuniculus*] | LNO | hydrolase activity acting on glycosyl bonds | -21.8 |
| 24 | FN1981 | HAV-FN-FN1981-2001-11-30.ab1 | Zgc:113383 - Danio rerio (Zebrafish) (*Brachydanio rerio*) | ME | transcription factor activity | -21.0 |
| 25 | L1T776 | L1T776-2006-12-21.ab1 | hypothetical protein - *Danio rerio* | L1T | unknown function | -16.3 |
| 26 | LF438 | LF438-2007-01-10.ab1 | no hit | LF | no hit | -14.0 |
| 27 | LNC835 | LNC835-2007-12-21.ab1 | similar to CG3456-PA - *Tribolium castaneum* | LNC | unknown function | -12.7 |
| 28 | CL31 Contig1 | zslaa0_008869.z1.scf; zslaa0_012245.z1.scf; FN3876-2003-02-11.ab1; LF147-2007-01-09.ab1; HAV-FN-FN2573-2001-12-13.ab1; 491_FN1375_C10_070.ab1; 494_FN1375-rerun_C08_054.ab1; L1T_1139-2007-01-15.ab1; L1T1386-2007-01-23.ab1; PU254-2004-09-21.ab1; L1T966-2007-01-11.ab1 | similar to CG10472-PA - *Tribolium castaneum* | FB, ME, L1T, PU, LF, LNO | serine proteinase | -10.9 |
| 29 | LNC994 | LNC994-2007-12-28.ab1 | no hit | LNC | no hit | -9.5 |
| 30 | CL3565 Contig1 | zslaa0_013592.z1.scf; zslaa0_014341.z1.scf | Longitudinals lacking protein-like [*Caligus rogercresseyi*] | LNO | Transcription factor that regulates axon guidance | -9.5 |
| 31 | CL19 Contig2 | zslaa0_014922.z1.scf; zslaa0_013286.z1.scf; zslaa0_015210.z1.scf; zslaa0_008019.z1.scf; PU581-2004-09-30.ab1; LNO924-2007-02-13.ab1; 519_FN1502_B01_090.ab1; LF1989-2007-05-09.ab1; L1T756-2006-12-21.ab1; FN3523-2002-03-25.ab1; LF1008-2007-03-30.ab1; LPA130-2007-05-15.ab1; FN5906-2005-03-15.ab1; LF145-2007-01-09.ab1; L1T422-2006-12-19.ab1; PU703-2004-12-02.ab1 | Phospholipid-hydroperoxide glutathione peroxidase - *Boophilus microplus* (Cattle tick) | FB, ME, L1T, PU, LF, LPA, LNO | Stress response/ development | -9.2 |
| 32 | zslaa0_006245 | zslaa0_006245.z1.scf | Nadp transhydrogenase - *Aedes aegypti* (Yellowfever mosquito) | LNO | oxidoreductase activity | -9.2 |
| 33 | FN3549, CL12 Contig2, FN3672, CL12 Contig1 | FN3549-2002-03-25.ab1;HAV-FN-FN2101-2001-11-30.ab1; FN3489-2002-03-08.ab1;FN3672-2002-09-18.ab1;356_FN559_SB32_T7.ab1; FN3614-2002-09-18.ab1; FN3683-2002-09-18.ab1; zslaa0_003249.z1.scf; FN4527-2003-03-05.ab1; FN3647-2002-09-18.ab1; FN3048-2002-02-18.ab1; FN4566-2003-03-05.ab1; FN4583-2003-03-05.ab1; HAV-FN-FN2451-rerun-2001-12-11.ab1; FN3624-2002-09-18.ab1; FN3610-2002-09-18.ab1; HAV-FN-FN2451-2001-12-10.ab1; HAV-FN-FN2466-2001-12-10.ab1; 365_FN601_SC4_T3.ab1; 356_FN556_SC5_T7.ab1; 365_FN600_SC3_T3.ab1; 365_FN602_SC10_T3.ab1; LNO541-2007-02-13.ab1; FN4582-2003-03-05.ab1; LNO2040-2007-02-20.ab1; 542_FN1771_G09_039.ab1; zslaa0_011369.z1.scf; FN3814-2003-02-11.ab1; 345_FN451_SC6_T7.ab1; 345_FN450_SC5_T7.ab1; 337_FN427_sc3_T7.ab1; 345_FN461_SC16_T7.ab1; 337_FN426_sc2_T7.ab1; 354_FN490_SE8_T7.ab1; HAV-FN-FN2584-2001-12-13.ab1; FN5928-2005-03-15.ab1; FN3837-2003-02-11.ab1; FN3545-2002-03-25.ab1; HAV-FN-FN1867-2001-11-28.ab1; HAV-FN-FN2145-2001-11-30.ab1; FN3254-2002-03-08.ab1; FN3436-2002-03-08.ab1; 542_FN1772_H09_047.ab1; FN3565-2002-03-25.ab1; FN3649-2002-09-18.ab1; FN3583-2002-03-25.ab1; FN3655-2002-09-18.ab1; FN5932-2005-03-15.ab1; FN5175-2005-01-17.ab1; L1T710-2006-12-21.ab1; FN3791-2003-01-16.ab1; HAV-FN-FN2738-2001-12-19.ab1; HAV-FN-FN2269-2001-11-30.ab1; FN6107-2005-04-05.ab1; FN4375-2003-02-19.ab1; FN4039-2003-02-18.ab1; HAV-FN-FN2817-2001-12-19.ab1; HAV-FN-FN2846-2001-12-19.ab1; HAV-FN-FN2711-2001-12-19.ab1; HAV-FN-FN1876-2001-11-28.ab1; FN4511-2003-03-05.ab1; FN4812-2003-03-12.ab1; FN4265-2003-02-18.ab1; HAV-FN-FN2392-2001-12-10.ab1; FN4789-2003-03-06.ab1; HAV-FN-FN2138-2001-11-30.ab1; HAV-FN-FN2135-2001-11-30.ab1; HAV-FN-FN2592-rerun-2001-12-16.ab1; HAV-FN-FN2592-2001-12-13.ab1; FN3959-2003-02-11.ab1; HAV-FN-FN1901-2001-11-28.ab1; FN4552-2003-03-05.ab1; 356_FN508_SB86_T7.ab1; HAV-FN-FN2834-2001-12-19.ab1 | vitellogenin 1 [*Lepeophtheirus salmonis*] | ME, SB, L1T, LNO | Egg york protein | -9.1 |
| 34 | NLG1154 | NLG1154-2007-08-03.ab1 | vitellogenin-like protein [*Lepeophtheirus salmonis*] | NLG | Egg york protein | -9.0 |
| 35 | zslaa0_004577 | zslaa0_004577.z1.scf | no hit | LNO | no hit | -8.6 |
| 36 | CL469 Contig1, zslaa0_007923 | zslaa0_011055.z1.scf; zslaa0_005713.z1.scf; zslaa0_001313.z1.scf; zslaa0_008183.z1.scf; LNO1596-2007-02-07.ab1; LNO2277-2007-02-27.ab1; LNO3038-2007-02-19.ab1; LPU1708-2007-09-05.ab1; LF451-2007-01-10.ab1; zslaa0_007923.z1.scf | sodium/solute symporter [*Aedes aegypti*] | LF, LPU, LNO | sodium ion transport | -7.5 |
| 37 | CL150 Contig1 | zslaa0_014350.z1.scf; zslaa0_006439.z1.scf; zslaa0_001880.z1.scf; zslaa0_009202.z1.scf; zslaa0_013901.z1.scf; zslaa0_005650.z1.scf; zslaa0_002358.z1.scf; zslaa0_017024.z1.scf; zslaa0_010856.z1.scf; zslaa0_016363.z1.scf; zslaa0_014447.z1.scf; LF1883-2007-05-08.ab1; LNO3730-2007-02-20.ab1; LF1766-2007-05-08.ab1; LNO3075-2007-02-19.ab1 | no hit | LF, LNO | no hit | -7.4 |
| 38 | CL603 Contig1 | zslaa0_007260.z1.scf; zslaa0_013658.z1.scf; LNC2648-2008-01-15.ab1; LNC2727-2008-01-16.ab1; LNC3137-2008-12-22.ab1; LNC2437-2008-01-16.ab1; L1T1609-2007-01-12.ab1; L1T1622-2007-01-12.ab1 | similar to cathD CG1548-PA - *Apis mellifera* | L1T, LNO, LNC | unknown function | -7.1 |
| 39 | zslaa0_006067 | zslaa0_006067.z1.scf | no hit | LNO | no hit | -7.1 |
| 40 | CL449 Contig1 | zslaa0_014301.z1.scf; zslaa0_002572.z1.scf; zslaa0_002557.z1.scf; zslaa0_015124.z1.scf; zslaa0_002173.z1.scf; zslaa0_009425.z1.scf; zslaa0_016683.z1.scf; LNO2686-2007-03-01.ab1; L1T554-2006-12-21.ab1 | granulin-like, partial [*Saccoglossus kowalevskii*] | L1T, LNO | lipid metabolism, embryonal development | -7.0 |
| 41 | zslaa0_013678 | zslaa0_013678.z1.scf | NAD(P) transhydrogenase[*Strongylocentrotus purpuratus*] | LNO | oxidoreductase activity | -6.9 |
| 42 | FN4968 | FN4968-2003-03-12.ab1 | no hit | ME | no hit | -6.9 |
| 43 | CL2734 Contig1 | zslaa0_016249.z1.scf; zslaa0_011896.z1.scf | Homolog of Homo sapiens "BTB/POZ domain containing protein 3 - *Takifugu rubripes* | LNO | unknown function | -6.8 |
| 44 | zslaa0_004239 | zslaa0_004239.z1.scf | no hit | LNO | no hit | -6.7 |
| 45 | CL2785 Contig1 | FN5551-2005-02-10.ab1; LNO3602-2007-03-09.ab1 | Alkaline phosphatase - *Aedes aegypti* (Yellowfever mosquito) | ME, LNO | metabolism | -6.3 |
| 46 | CL2270 Contig1 | NLG4652-2009-03-05.ab1; LNC2327-2008-01-15.ab1; FN3262-2002-03-08.ab1 | >gnl|BL_ORD_ID|799004 Tyrosine aminotransferase [*Danio rerio*] | ME, LNC, NLG | Amino acid metabolism | -6.1 |
| 47 | FN3790 | FN3790-2003-01-16.ab1 | similar to CG10444-PA isoform 1 - *Apis mellifera* | ME | unknown function | -5.9 |
| 48 | CL706 Contig1 | zslaa0_003677.z1.scf; zslaa0_010483.z1.scf; LNO568-2007-02-13.ab1; LNO2927-2007-03-07.ab1; NLG4764-2009-03-05.ab1; NLG3077-2007-09-26.ab1 | no hit | LNO, NLG | no hit | -5.8 |
| 49 | zslaa0_003619 | zslaa0_003619.z1.scf | no hit | LNO | no hit | -5.8 |
| 50 | CL202 Contig2 | zslaa0_005606.z1.scf; LNO936-2007-02-13.ab1; FN3544-2002-03-25.ab1 | no hit | ME, LNO | no hit | -5.7 |
| 51 | zslaa0_007975* | zslaa0_007975.z1.scf | *Lepeophtheirus salmonis* Atlantic form clone lsaA-evv-509-309 serine protease K12H4.7 putative mRNA, complete cds | LNO | serine protease | -5.7 |
| 52 | CL1714 Contig1 | LNC1074-2007-12-28.ab1; LPU1983-2007-09-05.ab1; FN3939-2003-02-11.ab1 | Advillin (p92). - *Xenopus tropicalis* | ME, LPU, LNC | nervous system development | -5.6 |
| 53 | FN1014 | 475_FN-1014-rerun_E10_071.ab1 | no hit | FB | no hit | -5.5 |
| 54 | CL2733 Contig1 | zslaa0_005621.z1.scf; LPU1847-2007-09-05.ab1 | no hit | LPU, LNO | no hit | -5.5 |
| 55 | zslaa0_005439 | zslaa0_005439.z1.scf | Cysteine-rich motor neuron 1 protein precursor [*Caligus clemensi*] | LNO | interacting with growth factors implicated in motor neuron differentiation and survival | -5.4 |
| 56 | NLG3690 | NLG3690-2009-02-09.ab1 | ENSANGP00000011321 - *Anopheles gambiae str*. PEST | NLG | hydrolase activity | -5.4 |
| 57 | NLG2894 | NLG2894-2007-09-18.ab1 | no hit | NLG | no hit | -5.3 |
| 58 | CL1127 Contig1 | zslaa0_014073.z1.scf; zslaa0_016043.z1.scf; zslaa0_012073.z1.scf; LNO3687-2007-03-09.ab1 | 7-dehydro-cholesterol reductase [*Lepeophtheirus salmonis*] | LNO | cholesterol production | -5.2 |
| 59 | PU822 | PU822-2005-03-14.ab1 | scavenger receptor class B, member 2 [*Oryctolagus cuniculus*] | PU | lipid transport | -5.2 |
| 60 | LNO2518 | LNO2518-2007-03-01.ab1 | acyl-CoA synthetase bubblegum family member 1-like | LNO | lipid metabolism | -5.2 |
| 61 | CL3240 Contig1 | LNC3595-2009-02-10.ab1; LNC125-2007-11-22.ab1 | tissue-nonspecific alkaline phosphatase-like [*Saccoglossus kowalevskii*] | LNC | hydrolase activity | -5.1 |
| 62 | LPU322 | LPU322-2007-08-29.ab1 | no hit | LPU | no hit | -5.0 |
| 63 | LNO3162 | LNO3162-2007-02-27.ab1 | CG13384-PC isoform C - *Drosophila melanogaster* (Fruit fly) | LNO | Amino acid transporter | -5.0 |
| 64 | CL1909 Contig1 | zslaa0_008094.z1.scf; LNC176-2007-11-22.ab1; L1T1630-2007-01-12.ab1 | 7-dehydro-cholesterol reductase - *Oryza sativa* subsp. indica (Rice) | L1T, LNO, LNC | oxidoreductase activity | -5.0 |
| 65 | CL3605 Contig1 | LNC3882-2009-03-06.ab1; LNC1951-2008-01-15.ab1 | Chromosome undetermined SCAF14025 whole genome shotgun sequence - *Tetraodon nigroviridis* (Green puffer) | LNC | nucleotide-sugar transport | -5.0 |
| 66 | CL1017 Contig1 | zslaa0_009980.z1.scf; zslaa0_012125.z1.scf; LNO2659-2007-03-01.ab1; LNO4089-2007-03-09.ab1; LNO3404-2007-03-05.ab1 | Gamma-interferon-inducible-lysosomal thiol reductase - *Pseudosciaena crocea* (Croceine croaker) | LNO | oxidoreductase activity | -4.9 |
| 67 | LNC3830 | LNC3830-2009-03-06.ab1 | Aspartic proteinase AspMD03 - *Musca domestica* (House fly) | LNC | hydrolase activity | -4.8 |
| 68 | CL1703 Contig1* | zslaa0_008501.z1.scf; 278_FN29_E04_023.ab1; L1T291-2006-12-04.ab1 | hypothetical protein - Monodelphis domestica; GB:*Danio rerio* CD36 antigen (cd36), mRNA | FB, L1T, LNO | ligand binding | -4.7 |
| 69 | CL2013 Contig1 | zslaa0_001622.z1.scf; zslaa0_014642.z1.scf; LPU1959-2007-09-05.ab1 | Intestinal trypsin 3 precursor - *Lepeophtheirus salmonis* (salmon louse) | LPU, LNO | Intestinal trypsin 3 precursor | -4.7 |
| 70 | CL575 Contig1 | LPU2046-2007-09-06.ab1; LNC1781-2008-01-24.ab1; FN4822-2003-03-12.ab1; FN5243-2005-01-19.ab1; LNC941-2007-12-28.ab1; LNO2545-2007-03-01.ab1; 482_FN1284_D11_090.ab1; FN4691-2003-03-06.ab1 | >gnl|BL_ORD_ID|1300950 conserved hypothetical protein [*Pediculus humanus corporis*] | FB, ME, LPU, LNO, LNC | juvenile hormone-inducible protein | -4.6 |
| 71 | FN4432 | FN4432-2003-03-05.ab1 | similar to CG3108-PA - *Apis mellifera* | ME | Carboxy-peptidase | -4.6 |
| 72 | PU579 | PU579-2004-09-30.ab1 | no hit | PU | no hit | -4.6 |
| 73 | CL96 Contig1* | FN4895-2003-03-12.ab1; zslaa0_010720.z1.scf; FN3953-2003-02-11.ab1; L1T194-2006-12-01.ab1 | similar to CG9953-PA - *Tribolium castaneum* | ME, L1T, LNO | Carboxy-peptidase | -4.6 |
| 74 | CL2740 Contig1 | zslaa0_001937.z1.scf; zslaa0_014096.z1.scf | no hit | LNO | no hit | -4.6 |
| 75 | FN5275 | FN5275-2005-01-19.ab1 | amino acid transporter protein [*Glossina morsitans morsitans*] | ME | Amino acid transporter | -4.5 |
| 76 | zslaa0_010260 | zslaa0_010260.z1.scf | no hit | LNO | no hit | -4.5 |
| 77 | CL1763 Contig1 | HAV-FN-FN2676-2001-12-13.ab1; HAV-FN-FN2676-rerun-2001-12-16.ab1; L1T1752-2007-01-24.ab1 | no hit | ME, L1T | no hit | -4.5 |
| 78 | CL2946 Contig1 | FN4781-2003-03-06.ab1; 292_CC501_A01_001.ab1 | RXR-like protein - *Biomphalaria glabrata* (Bloodfluke planorb) | ME, CC | Nuclear receptor | -4.4 |
| 79 | zslaa0_008255 | zslaa0_008255.z1.scf | apoptosis-inducing factor, mitochondrion-associated, 3 isoform 2 [*Bos taurus*] | LNO | apoptosis regulator | -4.3 |
| 80 | LNC1776 | LNC1776-2008-01-24.ab1 | Dipeptidyl-peptidase - *Aedes aegypti* (Yellowfever mosquito) | LNC | peptidase | -4.3 |
| 81 | CL1748 Contig1 | zslaa1_002765.z1.scf; zslaa0_016612.z1.scf; LNO2342-2007-02-27.ab1 | Zgc:56585 protein - *Danio rerio* (Zebrafish) (Brachydanio rerio) | LNO | lipid metabolism | -4.3 |
| 82 | CL441 Contig1 | zslaa0_005381.z1.scf; zslaa0_012995.z1.scf; zslaa0_005690.z1.scf; zslaa0_015469.z1.scf; zslaa0_008240.z1.scf; zslaa0_008159.z1.scf; zslaa0_014149.z1.scf; zslaa0_000306.z1.scf; zslaa0_0097_H04.ab1; LNO3437-2007-03-05.ab1 | no hit | LNO | no hit | -4.3 |
| 83 | CL68 Contig1 | zslaa0_017783.z1.scf; zslaa0_004495.z1.scf; zslaa0_013863.z1.scf; PU405-2004-09-30.ab1; zslaa0_008027.z1.scf; zslaa0_017059.z1.scf; zslaa0_014166.z1.scf; zslaa0_011841.z1.scf; zslaa0_004457.z1.scf; zslaa0_010527.z1.scf; zslaa0_016394.z1.scf; zslaa0_008227.z1.scf; zslaa0_007027.z1.scf; zslaa1_002780.z1.scf; PU341-2004-09-28.ab1; zslaa0_008248.z1.scf; LNO3320-2007-02-19.ab1; LNO3241-2007-02-15.ab1; FN3922-2003-02-11.ab1; FN3748-2003-01-16.ab1; FN3731-2003-01-16.ab1; L1T140-2006-12-01.ab1; PU491-2004-09-30.ab1; LPU1914-2007-09-05.ab1 | Aspartic proteinase AspMD03 - *Musca domestica* (House fly) | ME, L1T, PU, LPU, LNO | hydrolase activity | -4.3 |
| 84 | CL2596 Contig1 | zslaa0_013924.z1.scf; zslaa0_013471.z1.scf | Carboxypeptidase B [*Caligus clemensi*] | LNO | Carboxy-peptidase | -4.3 |
| 85 | zslaa0_008993 | zslaa0_008993.z1.scf | hypothetical protein - *Strongylocentrotus purpuratus* | LNO | oxidoreductase activity | -4.3 |
| 86 | zslaa0_011412 | zslaa0_011412.z1.scf | no hit | LNO | no hit | -4.2 |
| 87 | CL762 Contig1 | zslaa0_016944.z1.scf; zslaa1_010164.z1.scf; zslaa0_015451.z1.scf; LNO2425-2007-02-14.ab1; zslaa0_003652.z1.scf; NLG4621-2009-03-05.ab1 | Ultraspiracle nuclear receptor - *Tribolium castaneum* (Red flour beetle) | LNO, NLG | Nuclear receptor | -4.2 |
| 88 | CL1186 Contig1/2 | zslaa0_000149.z1.scf; FN3920-2003-02-11.ab1; LNO509-2007-02-13.ab1; FN4032-2003-02-18.ab1; FN3870-2003-02-11.ab1 | ENSANGP00000012364 - *Anopheles gambiae* str. PEST | ME, LNO | metabolic | -4.2 |
| 89 | CL1418 Contig1 | zslaa0_002522.z1.scf; zslaa0_015647.z1.scf; zslaa0_011718.z1.scf; LNC232-2007-12-18.ab1 | Trypsin - *Portunus pelagicus* (Blue swimmer crab)  Tryp 4 precursor =CL227Contig 1 | LNO, LNC | trypsin | -4.2 |
| 90 | zslaa0_016230* | zslaa0_016230.z1.scf | Legumain-like protease precursor - *Ixodes ricinus* (Sheep tick) | LNO | lysosomal cysteine protease | -4.2 |
| 91 | CL2403 Contig1 | zslaa0_003558.z1.scf; LNO3883-2007-03-15.ab1; LNC2416-2008-01-16.ab1 | Ferritin 1-like protein A - *Daphnia pulex* (Water flea) | LNO, LNC | oxidoreductase activity | -4.1 |
| 92 | LPU639 | LPU639-2007-09-18.ab1 | Putative serine proteinase - *Tenebrio molitor* (Yellow mealworm) | LPU | hydrolase activity | -4.1 |
| 93 | CL3426 Contig1 | NLG4627-2009-03-05.ab1; LNO573-2007-02-13.ab1 | Acetoacetyl-CoA synthetase - *Xenopus tropicalis* (Western clawed frog) (Silurana tropicalis) | LNO, NLG | lipid metabolism | -4.1 |
| 94 | LNO3617 | LNO3617-2007-03-09.ab1 | no hit | LNO | no hit | -4.0 |
| 95 | CL1696 Contig1* | zslaa0_005629.z1.scf; zslaa0_011072.z1.scf; zslaa0_008891.z1.scf | legumain-like protease precursor [*Ixodes ricinus*] | LNO | lysosomal cysteine protease | -4.0 |
| 96 | CL1136 Contig1 | zslaa0_012758.z1.scf; zslaa0_004211.z1.scf; zslaa0_014033.z1.scf; LNC228-2007-12-18.ab1; zslaa0_015944.z1.scf | Lysophosphatidylcholine acyltransferase 2-B [*Lepeophtheirus salmonis*] | LNO, LNC | lipid metabolism | -4.0 |
| 97 | CL227 Contig1* | zslaa0_005622.z1.scf; zslaa0_003087.z1.scf; zslaa0_011996.z1.scf; zslaa0_009372.z1.scf; zslaa0_002536.z1.scf; zslaa0_003149.z1.scf; zslaa0_000334.z1.scf; zslaa0_016757.z1.scf; zslaa0_008583.z1.scf; HAV-FN-FN2313-2001-12-10.ab1; LN01203-2007-02-08.ab1; 491_FN1380_H10_080.ab1; LNO814-2007-01-03.ab1; LNO2236-2007-02-27.ab1; LF1485-2007-04-23.ab1 | Intestinal trypsin 4 precursor - *Lepeophtheirus salmonis* (salmon louse) | FB, ME, LF, LNO | trypsin | -4.0 |
| 98 | CL2700 Contig1 | NA570-2003-05-30.ab1; LNO150-2006-12-01.ab1 | no hit | NA, LNO | no hit | -4.0 |
| 99 | CL769 Contig1 | zslaa0_015403.z1.scf; LN01344-2007-02-01.ab1; NLG3421-2009-02-09.ab1; zslaa0_005576.z1.scf; LNC3438-2009-02-09.ab1; LNC3526-2009-02-10.ab1 | no hit | LNO, LNC, NLG | no hit | -4.0 |
| 100 | FN462, FN4787, CL1933 Contig1, CL1582 Contig1, CL3 Contig1, CL1062 Contig1, FN825, LNO3333 | 345_FN462_SC17_T7.ab1; FN4787-2003-03-06.ab1;zslaa0_007690.z1.scf; FN5931-2005-03-15.ab1; FN5844rerun-2005-03-15.ab1;zslaa0_001863.z1.scf; zslaa0_009951.z1.scf; 383_FN836_ME136_T3.ab1; HA352-2004-08-13.ab1;FN5833-2005-03-14.ab1; FN4820-2003-03-12.ab1; FN3753-2003-01-16.ab1; FN5987-2005-03-15.ab1; zslaa0_018416.z1.scf; zslaa0_002555.z1.scf; FN4349-2003-02-19.ab1; zslaa0_001842.z1.scf; FN6142-2005-04-05.ab1; LNO3901-2007-02-27.ab1; zslaa0_008296.z1.scf; zslaa0_009196.z1.scf; HAV-FN-FN2202-2001-11-30.ab1; FN3538-2002-03-25.ab1; zslaa0_005307.z1.scf; zslaa0_015184.z1.scf; zslaa0_014754.z1.scf; FN4578-2003-03-05.ab1; zslaa0_012094.z1.scf; FN4252-2003-02-18.ab1; zslaa0_009393.z1.scf; 356_FN570_SB69_T7.ab1; FN3648-2002-09-18.ab1; FN4547-2003-03-05.ab1; FN3704-2003-01-16.ab1; FN3860-2003-02-11.ab1; FN3981-2003-02-11.ab1; HAV-FN-FN2148-2001-11-30.ab1; FN6060-2005-04-05.ab1; FN3025-2002-02-18.ab1; FN4204-2003-02-18.ab1; 356_FN529_SB52_T7.ab1; FN4282-2003-02-18.ab1; HAV-FN-FN2543-2001-12-13.ab1; FN4758-2003-03-06.ab1; 383_FN852_ME152_T3.ab1; zslaa0_017712.z1.scf; FN3946-2003-02-11.ab1; FN3851-2003-02-11.ab1; FN3510-2002-03-25.ab1; FN3775-2003-01-16.ab1; HAV-FN-FN1943-2001-11-30.ab1; HAV-FN-FN2830-2001-12-19.ab1; FN3815-2003-02-11.ab1; FN3643-2002-09-18.ab1; 356_FN519_SB27_T7.ab1; 356_FN535_SB29_T7.ab1; 356_FN563_SB81_T7.ab1; 356_FN590_SB24_T7.ab1; 356_FN505_SB49_T7.ab1; FN3635-2002-09-18.ab1; 356_FN523_SB76_T7.ab1; 356_FN582_SB23_T7.ab1; 356_FN586_SB71_T7.ab1; 356_FN585_SB59_T7.ab1; 356_FN587_SB84_T7.ab1; FN3658-2002-09-18.ab1; 357_FN587rerun_SB84_T7.ab1; FN3656-2002-09-18.ab1; 356_FN524_SB88_T7.ab1; 356_FN569_SB57_T7.ab1; 356_FN548_SB91_T7.ab1; 356_FN564_SB93_T7.ab1; 356_FN576_SB46_T7.ab1; 356_FN581_SB11_T7.ab1; 356_FN542_SB18_T7.ab1; 356_FN539_SB78_T7.ab1; 356_FN514_SB62_T7.ab1; 356_FN512_SB38_T7.ab1; 356_FN571_SB82_T7.ab1; 356_FN521_SB51_T7.ab1; 356_FN543_SB30_T7.ab1; 356_FN533_SB5_T7.ab1; 356_FN592_SB48_T7.ab1; 337_FN433_SE1_T7.ab1; 356_FN538_SB65_T7.ab1; 356_FN532_SB89_T7.ab1; 354_FN494_SE12_T7.ab1; 354_FN497_SE15_T7.ab1; 354_FN492_SE10_T7.ab1; FN3627-2002-09-18.ab1; FN3688-2002-09-18.ab1; 357_FN531rerun_SB77_T7.ab1; HAV-FN-FN1851-2001-11-28.ab1; HAV-FN-FN2859-2001-12-19.ab1; FN5155-2005-01-17.ab1; FN5915-2005-03-15.ab1; 345_FN469_SD9_T7.ab1; FN3354-2002-03-08.ab1; HAV-FN-FN1844-2001-11-28.ab1; HAV-FN-FN2120-2001-11-30.ab1; HAV-FN-FN2889-2001-12-19.ab1; HAV-FN-FN2376-2001-12-10.ab1; FN3221-2002-03-08.ab1; FN5960-2005-03-15.ab1; 542_FN1735_C05_005.ab1; FN4307-2003-02-19.ab1; FN3425-2002-03-08.ab1; FN6193-2005-04-05.ab1; HAV-FN-FN2376-rerun-2001-12-11.ab1; FN4919-2003-03-12.ab1; 356_FN549_SB7_T7.ab1; HAV-FN-FN2664-rerun-2001-12-16.ab1; HAV-FN-FN2664-2001-12-13.ab1; FN3426-2002-03-08.ab1; FN4564-2003-03-05.ab1; FN3348-2002-03-08.ab1; FN6072-2005-04-05.ab1; FN3267-2002-03-08.ab1; FN3874-2003-02-11.ab1; FN4090-2003-02-18.ab1; FN3881-2003-02-11.ab1; HAV-FN-FN2468-2001-12-10.ab1; FN3401-2002-03-08.ab1; FN4855-2003-03-12.ab1; FN3214-2002-03-08.ab1; HAV-FN-FN1906-2001-11-30.ab1;LNO2496-2007-02-14.ab1; FN4644-2003-03-06.ab1; FN3276-rerun-2002-03-12.ab1; FN4644rerun-2003-03-07.ab1; FN3263-2002-03-08.ab1;383_FN825_ME125_T3.ab1;LNO3333-2007-02-19.ab1 | vitellogenin 2 [*Lepeophtheirus salmonis*] | SB, ME, LNO, HA, LNO | Egg york protein | -4.0 |
| 101 | LF396, LNO3760 | LF396-2007-01-10.ab1; LNO3760-2007-02-20.ab1 | Juvenile hormone-inducible protein putative - *Aedes aegypti* (Yellowfever mosquito) | LF, LNO | Juvenile hormone-inducible protein | -4.0 |
| 102 | FN6180 | FN6180-2005-04-05.ab1 | no hit | ME | no hit | -4.0 |
| 103 | CL2590 Contig1 | zslaa0_007168.z1.scf; zslaa0_017103.z1.scf | similar to Probable cytochrome P450 304a1 (CYPCCCIVA1) - *Tribolium castaneum* | LNO | unknown function | -4.0 |
| 104 | LNC1004 | LNC1004-2007-12-28.ab1 | >gnl|BL_ORD_ID|2173285 predicted protein [*Nematostella vectensis*] | LNC | unknown function | -3.9 |
| 105 | FN1039* | 470_FN-1039_G05_052.ab1 | Cathepsin L cysteine protease ICP1 - *Ichthyophthirius multifiliis* (White spot Ich) | FB | cysteine-type peptidase activity | -3.9 |
| 106 | CL3808 Contig1 | LNO1745-2007-02-08.ab1; LIT302-2006-12-18.ab1 | no hit | L1T, LNO | no hit | -3.9 |
| 107 | CL1423 Contig1 | zslaa0_014190.z1.scf; zslaa0_018457.z1.scf; zslaa0_010037.z1.scf; LNC420-2007-12-21.ab1 | Troponin C isoform 1 - *Balanus nubilis* (Giant barnacle) | LNO, LNC | tropomyosin (muscle) | -3.9 |
| 108 | FN1168 | 481_FN1168_D09_074.ab1 | similar to cytochrome P450 family 2 subfamily J polypeptide 2 - *Canis familiaris* | FB | fatty acid, steroid metabolism | -3.9 |
| 109 | CL1932 Contig1 | LNC408-2007-12-21.ab1; LNC1010-2007-12-28.ab1; LNC1184-2008-01-17.ab1 | no hit | LNC | no hit | -3.8 |
| 110 | CL5 Contig7 | zslaa0_012317.z1.scf; zslaa0_015618.z1.scf; HA850-2005-02-25.ab1; LF214-2007-01-09.ab1; LPU1178-2007-08-29.ab1; LF385-2007-01-10.ab1; HA111-2004-07-28.ab1; LF786-2007-03-16.ab1 | *Lepeophtheirus salmonis* clone LS0008 hypothetical protein mRNA, partial cds | LF, LPU, HA, LNO | hypothetical protein [*Lepeophtheirus salmonis*] | -3.8 |
| 111 | CL2105 Contig1 | HAV-FN-FN2701-2001-12-19.ab1; L1T_1170-2007-01-15.ab1; LNC1427-2007-12-21.ab1 | Moesin/ezrin/radixin - *Aedes aegypti* (Yellowfever mosquito) | ME, L1T, LNC | embryo development | -3.8 |
| 112 | LNO2337 | LNO2337-2007-02-27.ab1 | *Lepeophtheirus salmonis* clone FN2958 hypothetical protein mRNA, partial cds | LNO | hypothetical protein [*Lepeophtheirus salmonis*] | -3.7 |
| 113 | CL3422 Contig1 | LNC2084-2008-01-15.ab1; LNC2286-2008-01-15.ab1 | similar to CG8129-PB isoform B - *Apis mellifera* | LNC | unknown function | -3.7 |
| 114 | CL1484 Contig1* | zslaa0_008182.z1.scf; FN5449-2005-02-10.ab1; LPU1059-2007-08-31.ab1; L1T1588-2007-02-26.ab1 | 32 kDa ferritin subunit - *Galleria mellonella* (Wax moth) | ME, L1T, LPU, LNO | iron storage | -3.7 |
| 115 | CL2883 Contig1 | zslaa0_007414.z1.scf; LNO3176-2007-02-27.ab1 | Na/Pi cotransport system protein - *Pseudopleuronectes americanus* (Winter flounder) (Pleuronectesamericanus) | LNO | phosphate transport | -3.6 |
| 116 | CL1876 Contig1 | zslaa0_007514.z1.scf; FN3042-2002-02-18.ab1; LNC2638-2008-01-15.ab1 | similar to CG7340-PB isoform B - *Tribolium castaneum* | ME, LNO, LNC | hydrolase activity | -3.6 |
| 117 | CL412 Contig1 | zslaa0_007147.z1.scf; zslaa0_016761.z1.scf; zslaa0_008771.z1.scf; zslaa0_015553.z1.scf; zslaa0_012494.z1.scf; L1T538-2006-12-21.ab1; LNO1779-2007-02-08.ab1; LNO3206-2007-02-15.ab1; LNO2267-2007-02-27.ab1; FN3492-rerun-2002-03-12.ab1; FN3492-2002-03-08.ab1 | Homolog of *Homo sapiens* "Aspartate aminotransferase cytoplasmic - *Takifugu rubripes* | ME, L1T, LNO | Amino acid metabolic | -3.6 |
| 118 | FN1227 | 484_FN1227-rerun_D06_046.ab1 | no hit | FB | no hit | -3.6 |
| 119 | zslaa0_013112 | zslaa0_013112.z1.scf | no hit | LNO | no hit | -3.6 |
| 120 | zslaa0_017772 | zslaa0_017772.z1.scf | no hit | LNO | no hit | -3.6 |
| 121 | CL586 Contig1 | zslaa0_002207.z1.scf; zslaa0_002301.z1.scf; zslaa0_008677.z1.scf; zslaa0_007274.z1.scf; zslaa0_010572.z1.scf; LNO2726-2007-03-06.ab1; LNC2921-2008-01-16.ab1; LNO1657-2007-02-15.ab1 | Vanin-like protein 1 precursor, putative [*Aedes aegypti*] | LNO, LNC | oxidative stress response | -3.5 |
| 122 | LNC1515 | LNC1515-2008-01-11.ab1 | AGAP010023-PA [*Anopheles gambiae str*. PEST]; similar to dumpy CG33196-PB (*Apis melifera*) | LNC | embryonal development | -3.5 |
| 123 | CL770 Contig1 | zslaa0_007076.z1.scf; zslaa0_000577.z1.scf; zslaa0_003720.z1.scf; zslaa0_003135.z1.scf; LNO1471-2007-02-14.ab1; LNO2015-2007-02-20.ab1; LNO2102-2007-03-06.ab1 | similar to CG8560-PA - *Tribolium castaneum* | LNO | Carboxy-peptidase | -3.5 |
| 124 | CL1968 Contig1 | zslaa0_004205.z1.scf; zslaa0_016716.z1.scf; LNO1437-2007-02-14.ab1 | no hit | LNO | no hit | -3.5 |
| 125 | CL3545 Contig1* | HAV-FN-FN1808-2001-11-28.ab1; FN4684-2003-03-06.ab1 | SJCHGC02838 protein - *Schistosoma japonicum* (Blood fluke) | ME | carbohydrate metabolic process | -3.5 |
| 126 | CL1150 Contig1, LNO3534 | zslaa0_011529.z1.scf; zslaa0_008057.z1.scf; NLG3747-2009-02-17.ab1; zslaa0_009174.z1.scf; LNO3534-2007-03-05.ab1 | Cytosolic nonspecific dipeptidase - Oreochromis niloticus (Nile tilapia) (*Tilapia nilotica*) | LNO, NLG | peptidase | -3.4 |
| 127 | zslaa0_000575 | zslaa0_000575.z1.scf | no hit | LNO | no hit | -3.4 |
| 128 | LF607 | LF607-2007-01-11.ab1 | no hit | LF | no hit | -3.4 |
| 129 | CL860 Contig1* | zslaa0_016822.z1.scf; FN5317-2005-01-26.ab1; LNC2438-2008-01-16.ab1; LF1282-2007-04-23.ab1; 383_FN834_ME134_T3.ab1; L1T1854-2007-01-24.ab1 | Niemann-Pick Type C-2 putative - *Aedes aegypti* (Yellowfever mosquito) | ME, L1T, LF, LNO, LNC | cholesterol homeostasis | -3.3 |
| 130 | LNO666 | LNO666-2007-02-06.ab1 | Synaptic glycoprotein SC2 [*Caligus rogercresseyi*] | LNO | lipid metabolism | -3.3 |
| 131 | zslaa0_016166, CL5 Contig8 | zslaa0_016166.z1.scf; zslaa0_014841.z1.scf; PU842-2005-03-14.ab1; zslaa0_006533.z1.scf; zslaa0_015088.z1.scf; zslaa0_008444.z1.scf; zslaa0_013451.z1.scf; zslaa0_017205.z1.scf; LF1009-2007-03-30.ab1; LF522-2007-01-10.ab1; LF1278-2007-04-23.ab1; LF557-2007-01-10.ab1; LPA168-2007-05-15.ab1; LF1409-2007-04-23.ab1; LNO2636-2007-03-01.ab1; LPU1289-2007-09-04.ab1; LF1773-2007-05-08.ab1; LF1511-2007-05-08.ab1; LF627-2007-01-11.ab1; LF1371-2007-05-08.ab1; LF479-2007-01-10.ab1; LF521-2007-01-10.ab1; LN01253-2007-02-08.ab1; LF329-2007-01-10.ab1; L1T1482-2007-01-24.ab1; LF1103-2007-05-08.ab1; LPA116-2007-05-15.ab1; LPU1310-2007-09-12.ab1; L1T567-2006-12-21.ab1; LPU470-2007-09-05.ab1; LPU1552-2007-09-04.ab1; L1T543-2006-12-21.ab1; PU757-2004-12-02.ab1; LF902-2007-03-16.ab1; L1T1423-2007-01-24.ab1 | *Lepeophtheirus salmonis* clone LS0027 hypothetical protein mRNA, complete cds | LNO, L1T, PU, LF, LPU, LPA | hypothetical protein [*Lepeophtheirus salmonis*] | -3.3 |
| 132 | LPU1001 | LPU1001-2007-08-31.ab1 | BCS-1 - *Balanus amphitrite* (Barnacle) | LPU | cuticle | -3.3 |
| 133 | CL36 Contig4 | zslaa0_012720.z1.scf; zslaa0_013176.z1.scf; zslaa0_017731.z1.scf; zslaa0_001627.z1.scf; zslaa0_010262.z1.scf; zslaa0_008264.z1.scf; LPU1040-2007-08-31.ab1; zslaa0_008051.z1.scf; LF1320-2007-05-08.ab1; LNO2272-2007-02-27.ab1; LNO2549-2007-03-01.ab1; LNO375-2007-01-12.ab1; L1T174-2006-12-01.ab1; L1T_1157-2007-01-15.ab1; PU784-2004-12-02.ab1; L1T167-2006-12-01.ab1 | Matrix metalloproteinase 9 - *Pseudacris regilla* (Pacific treefrog) | L1T, PU, LF, LPU, LNO | reproduction | -3.3 |
| 134 | zslaa0_010914 | zslaa0_010914.z1.scf | Myosin heavy chain type II - *Empusa sp*. IRT-2002 | LNO | muscle myosin complex | -3.3 |
| 136 | CL1538 Contig1* | zslaa0_011236.z1.scf; zslaa0_011103.z1.scf; zslaa0_019239.z1.scf; FN4273-2003-02-18.ab1 | Cluster: Phospholipase A2 isozymes PA3A/PA3B/PA5 - *Heloderma suspectum* (Gila monster) | ME, LNO | lipid degradation | -3.3 |
| 137 | LNO1712 | LNO1712-2007-02-08.ab1 | similar to CG31216-PA isoform 2 - *Apis mellifera* | LNO | unknown function | -3.2 |
| 138 | CL3619 Contig1 | LPU1078-2007-08-31.ab1; LNC2489-2008-01-16.ab1 | no hit | LPU, LNC | no hit | -3.2 |
| 139 | CL309 Contig1 | zslaa0_008185.z1.scf; zslaa0_017039.z1.scf; zslaa0_014103.z1.scf; zslaa0_012370.z1.scf; zslaa0_012937.z1.scf; zslaa0_011512.z1.scf; zslaa0_016287.z1.scf; LNO442-2007-02-08.ab1; NLG1424-2007-08-22.ab1; LNO1855-2007-02-16.ab1; LNC3262-2009-02-09.ab1; LNO2892-2007-03-07.ab1; FN3244-2002-03-08.ab1 | Saccharopine dehydrogenase domain-containing protein - *Aedes aegypti* (Yellowfever mosquito) | ME, LNO, LNC, NLG | Amino acid biosynthesis | -3.2 |
| 140 | CL16 Contig2, LPU639* | LF469-2007-01-10.ab1; zslaa0_004144.z1.scf; zslaa0_008615.z1.scf; zslaa0_016233.z1.scf; FN4723-2003-03-06.ab1; zslaa1_010122.z1.scf; zslaa0_011152.z1.scf; zslaa0_014150.z1.scf; zslaa0_011836.z1.scf; zslaa0_015825.z1.scf; zslaa0_004219.z1.scf; zslaa0_000525.z1.scf; 481_FN1169_E09_067.ab1; zslaa0_007595.z1.scf; zslaa0_010790.z1.scf; zslaa0_002105.z1.scf; LNO1746-2007-02-08.ab1; HA853-2005-02-25.ab1; FN5419-2005-02-10.ab1; FN5577-2005-02-10.ab1; LF143-2007-01-09.ab1; LNO2624-2007-03-01.ab1; FN3367-2002-03-08.ab1; 512_FN1494_F12_095.ab1; FN4723rerun-2003-03-07.ab1; FN5318-2005-01-26.ab1; 481_FN1118_B03_025.ab1; FN5277-2005-01-19.ab1; FN5227-2005-01-19.ab1; LF814-2007-02-12.ab1; 519_FN1570_F09_060.ab1; 520_FN1677_E10_056.ab1; 470_FN-1041_A06_053.ab1; 278_FN24_H03_028.ab1; HAV-FN-FN2840-2001-12-19.ab1; LNO3366-2007-02-19.ab1; 471_FN-949_FB49_T3.ab1; LNO1893-2007-02-16.ab1; 471_FN-974_FB74_T3..ab1; FN5259-2005-01-19.ab1; 471_FN-925_FB25_T3.ab1; LNO1427-2007-02-14.ab1; L1T956-2007-01-11.ab1; FN4688-2003-03-06.ab1; HAV-FN-FN2285-2001-11-30.ab1; LF101-2007-01-09.ab1; LNO1070-2007-02-01.ab1; HAV-FN-FN2374-2001-12-10.ab1; LF1554-2007-05-08.ab1; FN5323-2005-01-26.ab1; FN5707-2005-02-14.ab1; FN5336-2005-01-26.ab1 | Putative serine proteinase - *Tenebrio molitor* (Yellow mealworm) | FB, ME, L1T, LF, HA, LNO | proteinase | -3.2 |
| 141 | CL2697 Contig1 | zslaa0_015149.z1.scf; LNO3192-2007-02-27.ab1 | L-amino-acid oxidase - *Gallus gallus* (Chicken) | LNO | oxidoreductase | -3.2 |
| 142 | CL3792 Contig1 | zslaa0_015740.z1.scf; zslaa0_017287.z1.scf | no hit | LNO | no hit | -3.2 |
| 143 | CL619 Contig1 | zslaa0_015428.z1.scf; HAV-FN-FN2593-2001-12-13.ab1; LNO3259-2007-02-15.ab1; 481_FN1147_G06_040.ab1; NLG937-2007-06-21.ab1; HAV-FN-FN1862-2001-11-28.ab1 | *Lepeophtheirus salmonis* mitochondrion, complete genome | FB, ME, LNO, NLG | hypothetical protein [*Lepeophtheirus salmonis*], mitochndrion | -3.2 |
| 144 | zslaa0_009602* | zslaa0_009602.z1.scf | hypothetical protein isoform 2 - *Gallus gallus*, Gallus gallus finished cDNA, clone ChEST278m1 | LNO | anti coagulation | -3.2 |
| 145 | CL2314 Contig1* | 481_FN1186_F11_091.ab1; LNC811-2007-12-21.ab1; LNO3364-2007-02-19.ab1 | Dehydrogenase/reductase SDR family member 11 precursor - *Homo sapiens* (Human) | FB, LNO, LNC | oxidation-reduction process | -3.2 |
| 146 | CL2742 Contig1 | zslaa0_017182.z1.scf; zslaa0_015343.z1.scf | Multiple coagulation factor deficiency protein 2 homolog precursor - *Mus musculus* (Mouse) | LNO | secretion of coagulation factors, neurogenesis | -3.2 |
| 147 | CL1286 Contig1* | zslaa0_010287.z1.scf; LNO3789-2007-02-20.ab1; LF1996-2007-05-09.ab1; 542_FN1748_H06_027.ab1 | no hit | ME, LF, LNO | no hit | -3.2 |
| 148 | CL2419 Contig1 | zslaa0_009186.z1.scf; zslaa0_016126.z1.scf | no hit | LNO | no hit | -3.2 |
| 149 | CL64 Contig1* | LPU1992-2007-09-05.ab1; LPU860-2007-08-29.ab1; FN4336-2003-02-19.ab1; FN4673-2003-03-06.ab1; HAV-FN-FN2110-2001-11-30.ab1; LF1718-2007-05-08.ab1; 471_FN-980_FB80_T3..ab1; 471_FN-962_FB62_T3.ab1; HAV-FN-FN2521-rerun-2001-12-16.ab1; HAV-FN-FN2521-2001-12-13.ab1; FN3892-2003-02-11.ab1; 278_FN7_G01_004.ab1; 278_FN15_G02_008.ab1; HA338-2004-08-13.ab1; FN4438-2003-03-05.ab1; FN4179-2003-02-18.ab1; FN5955-2005-03-15.ab1; 512_FN1480_H10_080.ab1; FN4072-2003-02-18.ab1; FN4072-rerun-2003-02-19.ab1; FN5312-2005-01-26.ab1; 475_FN-1006-rerun_A10_069.ab1; 520_FN1692_D12_079.ab1; FN5578-2005-02-10.ab1; HAV-FN-FN2259-2001-11-30.ab1 | Putative uncharacterized protein - *Dictyostelium discoideum* AX4 | FB, ME, LF, LPU, HA | lipid metabolic process | -3.2 |
| 150 | FN6070* | FN6070-2005-04-05.ab1 | Glutathione peroxidase - *Aedes aegypti* (Yellowfever mosquito) | ME | Stress response | -3.1 |
| 151 | CL941 Contig1* | zslaa0_008961.z1.scf; FN5731-2005-02-14.ab1; zslaa0_010704.z1.scf; LNO2582-2007-03-01.ab1; HAV-FN-FN2158-2001-11-30.ab1; HAV-FN-FN1996-2001-11-30.ab1 | no hit | ME, LNO | no hit | -3.1 |
| 152 | LNO3643 | LNO3643-2007-03-09.ab1 | Homolog of *Scomber japonicus* "Endoplasmic reticulum lumenal L-amino acid oxidase precursor. - *Takifugu rubripes* | LNO | oxidoreductase activity | -3.1 |
| 153 | CL953 Contig1 | zslaa0_016465.z1.scf; HAV-FN-FN2731-2001-12-19.ab1; LF634-2007-01-11.ab1; 278_FN76_D10_078.ab1; LNO1061-2007-02-01.ab1 | no hit | FB, ME, LF, LNO | no hit | -3.1 |
| 154 | CL1632 Contig1 | zslaa0_011547.z1.scf; zslaa0_014167.z1.scf; NLG2195-2007-09-26.ab1; PU233-2004-09-21.ab1 | *Lepeophtheirus salmonis* clone PU676 hypothetical protein mRNA, complete cds | PU, LNO, NLG | hypothetical protein [*Lepeophtheirus salmonis*] | -3.1 |
| 155 | LPU1588 | LPU1588-2007-09-04.ab1 | no hit | LPU | no hit | -3.1 |
| 156 | NLG1620 | NLG1620-2007-08-23.ab1 | transmembrane protein C2orf18 homolog precursor [*Rattus norvegicus*] | NLG | carbohydrate transport | -3.1 |
| 157 | CL2931 Contig1, CL335 Contig1 | zslaa0_015806.z1.scf; FN4941-2003-03-12.ab1; zslaa0_015083.z1.scf; zslaa0_013527.z1.scf; zslaa0_011242.z1.scf; zslaa0_017001.z1.scf; zslaa0_012691.z1.scf; zslaa0_014675.z1.scf; zslaa0_014069.z1.scf; LNO3343-2007-02-19.ab1; LNO3422-2007-03-05.ab1; LNO2965-2007-03-07.ab1; LNO2660-2007-03-01.ab1; LNO3814-2007-03-15.ab1 | Troponin I - Pontastacus leptodactylus (Narrow-fingered crayfish) (*Astacusleptodactylus*) | ME, LNO | organ development | -3.1 |
| 158 | CL3625 Contig1 | zslaa1_010176.z1.scf; zslaa0_000854.z1.scf | similar to fumble CG5725-PE isoform E - *Apis mellifera* | LNO | unknown function | -3.1 |
| 159 | CL3221 Contig1* | zslaa0_015342.z1.scf; zslaa0_011678.z1.scf | Chitinase - *Araneus ventricosus* | LNO | Chitin degradation | -3.1 |
| 160 | CL1170 Contig1 | LNO973-2007-02-13.ab1; FN4740-2003-03-06.ab1; LNC3287-2009-02-09.ab1; FN3470-2002-03-08.ab1 | mitochondrial uncoupling protein 2 [*Dicrostonyx groenlandicus*] (northern collared lemming) | ME, LNO, LNC | mitochondrial transport | -3.1 |
| 161 | CL861 Contig1* | zslaa0_007430.z1.scf; zslaa0_013747.z1.scf; zslaa0_014814.z1.scf; zslaa0_016181.z1.scf; LF1207-2007-04-23.ab1; LNC117-2007-11-22.ab1 | no hit | LF, LNO, LNC | no hit | -3.1 |
| 162 | CL5 Contig3, LPU345 | HA312-2004-08-13.ab1; LF1816-2007-05-08.ab1 | *Lepeophtheirus salmonis* clone LS0063 hypothetical protein mRNA, complete cds | LF, HA, LPU | hypothetical protein [*Lepeophtheirus salmonis*] | -3.1 |
| 163 | LNO3885 | LNO3885-2007-03-15.ab1 | >gnl|BL_ORD_ID|2909833 uncharacterized conserved protein [*Sphaerobacter thermophilus* DSM 20745] | LNO | unknown function | -3.0 |
| 164 | zslaa0_005308 | zslaa0_005308.z1.scf | similar to CG7144-PA - *Tribolium castaneum* | LNO | metabolic | -3.0 |
| 165 | CL1092 Contig1 | zslaa0_012850.z1.scf; zslaa1_002743.z1.scf; zslaa0_001925.z1.scf; LPU1506-2007-09-04.ab1 | similar to CG2010-PA isoform A isoform 1 - *Apis mellifera* | LPU, LNO | unknown function | -3.0 |
| 166 | L1T949 | L1T949-2007-01-11.ab1 | no hit | L1T | no hit | -3.0 |
| 167 | CL557 Contig1* | zslaa0_007133.z1.scf; zslaa0_007508.z1.scf; zslaa0_018283.z1.scf; zslaa0_008571.z1.scf; FN4575-2003-03-05.ab1; FN3761-2003-01-16.ab1; L1T1542-2007-02-26.ab1; HA973-2005-03-02.ab1 | Chromosome undetermined SCAF14677 whole genome shotgun sequence - *Tetraodon nigroviridis* (Green puffer) | ME, L1T, HA, LNO | serine-type endopeptidase inhibitor | -3.0 |
| 168 | CL276 Contig1 | zslaa0_012406.z1.scf; zslaa0_012237.z1.scf; zslaa0_017004.z1.scf; zslaa0_012628.z1.scf; zslaa0_015628.z1.scf; zslaa1_002750.z1.scf; L1T926-2007-01-11.ab1; LNO3747-2007-02-20.ab1; LPU2014-2007-09-06.ab1; L1T1417-2007-01-24.ab1; LNO3018-2007-02-19.ab1; LNC2617-2008-01-15.ab1 | hypothetical protein - *Ornithorhynchus anatinus* | L1T, LPU, LNO, LNC | unknown function | -3.0 |
| 169 | LF506 | LF506-2007-01-10.ab1 | Muscle lim protein - *Aedes aegypti* (Yellowfever mosquito) | LF | embryonic development (regulator of myogenesis) | -2.9 |
| 170 | CL2036 Contig1 | NLG2325-2007-09-14.ab1; LPU1192-2007-08-29.ab1; zslaa0_009869.z1.scf | similar to CG14590-PA - *Tribolium castaneum* | LPU, LNO, NLG | metabolic | -2.9 |
| 171 | CL2683 Contig1* | zslaa0_013638.z1.scf; LNO3570-2007-03-05.ab1 | ENSANGP00000020429 - *Anopheles gambiae str.* PEST | LNO | unknown | -2.9 |
| 172 | zslaa0_002854 | zslaa0_002854.z1.scf | no hit | LNO | no hit | -2.9 |
| 173 | CL2326 Contig1* | L1T1384-2007-01-23.ab1; LNO4023-2007-03-09.ab1; NLG2387-2007-09-14.ab1 | similar to Tetraspanin 2A CG11415-PA - *Apis mellifera* | L1T, LNO, NLG | brain development | -2.9 |
| 174 | LNC2764 | LNC2764-2008-01-16.ab1 | Viral A-type inclusion protein repeat - *Entamoeba histolytica* HM-1:IMSS | LNC | unknown function | -2.9 |
| 175 | CL2942 Contig1 | zslaa0_004217.z1.scf; zslaa0_016445.z1.scf | no hit | LNO | no hit | -2.9 |
| 176 | CL3309 Contig1 | HAV-FN-FN2221-2001-11-30.ab1; HAV-FN-FN2221-rerun-2001-12-03.ab1 | no hit | ME | no hit | -2.9 |
| 177 | LNC150 | LNC150-2007-11-22.ab1 | Transport and Golgi organization 5 [*Drosophila melanogaster*] | LNC | intra-Golgi protein transport | -2.9 |
| 178 | CL301 Contig1* | zslaa0_014966.z1.scf; zslaa0_005526.z1.scf; LF1163-2007-05-08.ab1; zslaa0_011889.z1.scf; zslaa0_015446.z1.scf; LNC3004-2008-01-16.ab1; LNC931-2007-12-28.ab1; L1T1075-2007-01-02.ab1; FN5490-2005-02-10.ab1; FN3467-2002-03-08.ab1; FN4003-2003-02-18.ab1; HAV-FN-FN2302-2001-12-10.ab1 | similar to Neprilysin 1 CG5905-PA isoform A isoform 1 - *Apis mellifera* | ME, L1T, LF, LNO, LNC | proteolysis | -2.9 |
| 179 | CL1617 Contig1 | zslaa0_011583.z1.scf; zslaa0_014383.z1.scf; zslaa0_003654.z1.scf; LNO3713-2007-02-20.ab1 | Transmembrane protein 49 [*Caligus rogercresseyi*] | LNO | Stress response | -2.9 |
| 180 | CL1171 Contig1 | zslaa0_009209.z1.scf; zslaa0_016021.z1.scf; L1T1865-2007-01-24.ab1; LNO3675-2007-03-09.ab1; NLG581-2007-06-04.ab1 | ENSANGP00000003319 - *Anopheles gambiae str*. PEST | L1T, LNO, NLG | metabolic | -2.9 |
| 181 | CL2649 Contig1 | zslaa0_008570.z1.scf; LNC1211-2007-12-21.ab1 | Muscle lim protein - Aedes aegypti (Yellowfever mosquito) | LNO, LNC | embryonic development (regulator of myogenesis) | -2.9 |
| 182 | LNO1419 | LNO1419-2007-02-14.ab1 | sugar transport protein-like [*Oryza sativa* Japonica Group] | LNO | sugar transport | -2.9 |
| 183 | NLG1076 | NLG1076-2007-06-22.ab1 | no hit | NLG | no hit | -2.8 |
| 184 | zslaa0_016213 | zslaa0_016213.z1.scf | similar to Uncharacterized MFS-type transporter C19orf28 homolog [*Ciona intestinalis*] | LNO | sugar transport | -2.8 |
| 185 | CL256 Contig1, zslaa0_011121 | zslaa0_006779.z1.scf; zslaa0_015261.z1.scf; zslaa0_015185.z1.scf; zslaa0_001361.z1.scf; zslaa0_012004.z1.scf; zslaa0_004566.z1.scf; zslaa0_007045.z1.scf; zslaa0_000396.z1.scf; zslaa0_016089.z1.scf; LNO3655-2007-03-09.ab1; LNO3057-2007-02-19.ab1; LNO1692-2007-02-15.ab1; LNO3081-2007-02-19.ab1; zslaa0_011121.z1.scf | *Lepeophtheirus salmonis* Atlantic form clone lsaA-evv-512-310  Carboxypeptidase B putative mRNA, complete cds | LNO | Carboxy-peptidase | -2.8 |
| 186 | LNC2696 | LNC2696-2008-01-15.ab1 | Putative uncharacterized protein - *Aedes aegypti* (Yellowfever mosquito) | LNC | unknown function | -2.8 |
| 187 | LPU1929 | LPU1929-2007-09-05.ab1 | Innexin inx2 - *Drosophila melanogaster* (Fruit fly) | LPU | unknown function | -2.8 |
| 188 | LNC2822* | LNC2822-2008-01-15.ab1 | no hit | LNC | no hit | -2.8 |
| 189 | CL3344 Contig1 | LNC1889-2008-01-14.ab1; LNO1826-2007-02-16.ab1 | Neuronal acetylcholine receptor subunit alpha-3 precursor [*Caligus clemensi*] | LNO, LNC | neurotransmitter receptor activity | -2.7 |
| 190 | CL3349 Contig1* | LF259-2007-01-09.ab1; LNC1638-2008-01-14.ab1 | CG6982-PA - *Drosophila melanogaster* (Fruit fly) | LF, LNC | establishment or maintenance of cell polarity | -2.7 |
| 191 | CL1013 Contig1 | zslaa0_012084.z1.scf; LNC3101-2008-12-22.ab1; LNO3783-2007-02-20.ab1; LNC218-2007-12-18.ab1; L1T1875-2007-01-24.ab1 | twitchin [*Mytilus galloprovincialis*] | L1T, LNO, LNC | muscle protein | -2.7 |
| 192 | CL1881 Contig1 | zslaa0_009823.z1.scf; zslaa0_012367.z1.scf; zslaa0_014010.z1.scf | Hexokinase 3 - *Anopheles arabiensis* (Mosquito) | LNO | glycolysis | -2.7 |
| 193 | CL2205 Contig1 | zslaa0_006223.z1.scf; LNC2702-2008-01-16.ab1; LPA189-2007-05-15.ab1 | Tropomyosin-2 - *Bombyx mori* (Silk moth) | LPA, LNO, LNC | muscle contraction | -2.7 |
| 194 | CL3601 Contig1* | zslaa0_013303.z1.scf; zslaa0_004206.z1.scf | *Gasterosteus aculeatus* Rhesus-associated glycoprotein mRNA, complete cds | LNO | Ammonia transport | -2.7 |
| 195 | CL643 Contig1 | zslaa0_008115.z1.scf; zslaa0_002017.z1.scf; LNO3283-2007-02-15.ab1; LF122-2007-01-09.ab1; zslaa0_009690.z1.scf; LNC1847-2008-01-14.ab1 | Putative uncharacterized protein - *Stenotrophomonas maltophilia* R551-3 | LF, LNO, LNC | succinate-CoA ligase (ADP-forming) activity | -2.7 |
| 196 | zslaa0_010985 | zslaa0_010985.z1.scf | no hit | LNO | no hit | -2.7 |
| 197 | CL2113 Contig1 | LNC3701-2009-03-06.ab1; NLG2220-2007-09-10.ab1; LNC3208-2009-02-09.ab1 | no hit | LNC, NLG | no hit | -2.7 |
| 198 | zslaa0_008589 | zslaa0_008589.z1.scf | no hit | LNO | no hit | -2.7 |
| 199 | CL1839 Contig1 | zslaa0_014429.z1.scf; NLG3028-2007-09-26.ab1; LNC191-2007-11-22.ab1 | ENSANGP00000016715 - *Anopheles gambiae str.* PEST | LNO, LNC, NLG | unknown function | -2.7 |
| 200 | CL1067 Contig1,2 | L1T563-2006-12-21.ab1; zslaa0_012225.z1.scf; LPU1568-2007-09-04.ab1; zslaa0_011332.z1.scf; LPU319-2007-08-29.ab1 | CG6188-PA - *Drosophila melanogaster* (Fruit fly) | L1T, LPU, LNO | methionine metabolic process | -2.7 |
| 201 | zslaa0_002323 | zslaa0_002323.z1.scf | *Lepeophtheirus salmonis* clone LS0033 glyceraldehyde-3-phosphate dehydrogenase mRNA, complete cds | LNO | hypothetical protein [*Lepeophtheirus salmonis*] | -2.6 |
| 202 | LN01263 | LN01263-2007-02-08.ab1 | ENSANGP00000018278 - *Anopheles gambiae str.* PEST | LNO | unknown function | -2.6 |
| 203 | CL1496 Contig1 | LPU678-2007-09-18.ab1; L1T1801-2007-01-24.ab1; LPU428-2007-09-05.ab1; LPU1383-2007-09-12.ab1 | similar to F38B2.4 - *Tribolium castaneum* | L1T, LPU | nucleobase, nucleoside, nucleotide and nucleic acid metabolic process | -2.6 |
| 204 | LNO596 | LNO596-2007-02-13.ab1 | Serine protease inhibitor serpin 1b - *Mamestra configurata* (bertha armyworm) | LNO | serine protease inhibitor | -2.6 |
| 205 | CL718 Contig1* | zslaa0_003521.z1.scf; zslaa1_002790.z1.scf; L1T514-2006-12-21.ab1; zslaa0_015482.z1.scf; LPU1321-2007-09-12.ab1; L1T166-2006-12-01.ab1; LPU373-2007-08-29.ab1 | Cation-transporting ATPase - *Procambarus clarkii* (Red swamp crayfish) | L1T, LPU, LNO | ion transport | -2.6 |
| 206 | CL1346 Contig1 | zslaa0_014305.z1.scf; zslaa0_011530.z1.scf; NLG2702-2007-09-17.ab1; LNC2888-2008-01-15.ab1 | similar to Sphingosine-1-phosphate lyase CG8946-PA isoform A - *Apis mellifera* | LNO, LNC, NLG | unknown function | -2.6 |
| 207 | CL1349 Contig1 | NLG3266-2008-12-22.ab1; LNC2457-2008-01-16.ab1 | no hit | LNC, NLG | no hit | -2.6 |
| 208 | CL957 Contig1 | zslaa0_005280.z1.scf; zslaa0_016440.z1.scf; LNO2944-2007-03-07.ab1; LNC1360-2007-12-21.ab1; LNC2367-2008-01-15.ab1; LNC1634-2008-01-14.ab1 | similar to CG6398-PA - *Tribolium castaneum* | LNO, LNC | unknown function | -2.6 |
| 209 | CL2030 Contig1 | zslaa0_012964.z1.scf; LPU1531-2007-09-04.ab1 | no hit | LPU, LNO | no hit | -2.6 |
| 210 | L1T451 | L1T451-2006-12-19.ab1 | similar to CG9297-PA isoform A - *Apis mellifera* | L1T | calcium signalling | -2.6 |
| 211 | LNO261* | LNO261-2007-01-02.ab1 | no hit | LNO | no hit | -2.6 |
| 212 | CL1749 Contig1 | zslaa0_000847.z1.scf; NLG1774-2007-08-23.ab1; LNO4-2006-12-04.ab1 | similar to CG17086-PA - *Tribolium castaneum* | LNO, NLG | transcription repressor | -2.6 |
| 213 | LNC2705* | LNC2705-2008-01-16.ab1 | no hit | LNC | no hit | -2.6 |
| 214 | CL3271 Contig1 | LNO4036-2007-03-09.ab1; NLG1519-2007-08-23.ab1 | similar to CG5994-PA - *Tribolium castaneum* | LNO, NLG | transcription repressor (Negative elongation factor E) | -2.6 |
| 215 | zslaa0_010489 | zslaa0_010489.z1.scf | delta protein [*Parhyale hawaiensis*] | LNO | cell differentiation | -2.5 |
| 216 | LF395 | LF395-2007-01-10.ab1 | similar to inhibitor of apoptosis 2 protein [*Tribolium castaneum*] | LF | inhibitor of apoptosis | -2.5 |
| 217 | NLG1163 | NLG1163-2007-08-03.ab1 | no hit | NLG | no hit | -2.5 |
| 218 | CL3327 Contig1* | LNO1896-2007-02-16.ab1; LNO2521-2007-03-01.ab1 | similar to CG7378-PA - *Apis mellifera* | LNO | protein dephosphorylation | -2.5 |
| 219 | LNO3588 | LNO3588-2007-03-05.ab1 | COG0076: Glutamate decarboxylase and related PLP-dependent proteins - *Nostoc punctiforme* PCC 73102 | LNO | Stress response | -2.5 |
| 220 | CL75 Contig1* | zslaa0_001905.z1.scf; zslaa0_002116.z1.scf; L1T1339-2007-01-23.ab1; 494_FN1359-rerun_G07_052.ab1; 491_FN1359_C08_054.ab1; 519_FN1528_D04_015.ab1; HAV-FN-FN2857-rerun-2001-12-20.ab1; HAV-FN-FN2857-2001-12-19.ab1; FN4194-2003-02-18.ab1; HAV-FN-FN2647-2001-12-13.ab1; L1T1437-2007-01-24.ab1; 472_FN-1088_D02_026.ab1; LPU1271-2007-09-04.ab1; L1T_1247-2007-01-15.ab1; FN3912-2003-02-11.ab1; PU492-2004-09-30.ab1; 364_FN790_ME90_T3.ab1; 475_FN-970-rerun_G11_084.ab1; 482_FN1286_F11_091.ab1; FN4038-2003-02-18.ab1; HAV-FN-FN2770-2001-12-19.ab1; L1T1412-2007-01-24.ab1 | Cellular retinoic acid/retinol binding protein - *Metapenaeus ensis* (Greasyback shrimp) (Sand shrimp) | FB, ME, L1T, PU, LPU, LNO | lipid binding | -2.5 |
| 221 | FN1348 | 491_FN1348_H06_048.ab1 | no hit | FB | no hit | -2.5 |
| 222 | LNC2304 | LNC2304-2008-01-15.ab1 | no hit | LNC | no hit | -2.5 |
| 223 | L1T1001 | L1T1001-2007-01-02.ab1 | no hit | L1T | no hit | -2.5 |
| 224 | zslaa0_015685 | zslaa0_015685.z1.scf | no hit | LNO | no hit | -2.5 |
| 225 | FN4496 | FN4496-2003-03-05.ab1 | 60S ribosomal protein L17 [*Lepeophtheirus salmonis*] | ME | ribosome | -2.5 |
| 226 | CL2552 Contig1 | zslaa0_010449.z1.scf; LNC1996-2008-01-15.ab1 | no hit | LNO, LNC | no hit | -2.5 |
| 227 | LNO1410 | LNO1410-2007-02-14.ab1 | Acetyl-CoA acetyltransferase - *Sphingomonas wittichii* RW1 | LNO | metabolism | -2.5 |
| 228 | LNC1495* | LNC1495-2007-12-21.ab1 | Hexokinase - *Aedes aegypti* (Yellowfever mosquito) | LNC | glycolysis | -2.5 |
| 229 | LNC2251 | LNC2251-2008-01-15.ab1 | ENSANGP00000020045 - *Anopheles gambiae str.* PEST | LNC | unknown function | -2.5 |
| 230 | zslaa0_004429 | zslaa0_004429.z1.scf | no hit | LNO | no hit | -2.5 |
| 231 | NLG821 | NLG821-2007-06-14.ab1 | similar to CG4898-PE isoform E - *Tribolium castaneum* | NLG | muscle contraction | -2.5 |
| 232 | LNO339 | LNO339-2007-01-12.ab1 | similar to dehydrodolichyl diphosphate synthase *- Apis mellifera* | LNO | Catalyzes cis-prenyl chain elongation to produce the polyprenyl backbone of dolichol | -2.5 |
| 233 | CL651 Contig1 | zslaa0_016815.z1.scf; zslaa0_004509.z1.scf; zslaa0_005380.z1.scf; zslaa0_002828.z1.scf; zslaa0_017748.z1.scf; LNO255-2007-01-02.ab1; LNC3547-2009-02-10.ab1; LF1058-2007-03-30.ab1 | Chromosome undetermined scaffold_122 whole genome shotgun sequence - *Paramecium tetraurelia* | LF, LNO, LNC | unknown function | -2.5 |
| 234 | CL921 Contig1 | zslaa0_016239.z1.scf; zslaa0_013173.z1.scf; zslaa0_007320.z1.scf; zslaa0_007930.z1.scf; LNO3683-2007-03-09.ab1; LNC3852-2009-03-06.ab1 | CD63 antigen [*Lepeophtheirus salmonis*] | LNO, LNC | signal transduction | -2.5 |
| 235 | CL304 Contig1 | zslaa0_011514.z1.scf; zslaa0_016468.z1.scf; zslaa0_013919.z1.scf; zslaa0_016706.z1.scf; zslaa0_011105.z1.scf; zslaa0_015511.z1.scf; LNO311-2007-01-12.ab1; LNO2303-2007-02-27.ab1; LNO2926-2007-03-07.ab1; LNO1975-2007-02-19.ab1; NLG3627-2009-02-09.ab1; LNC1725-2008-01-24.ab1 | Beta-taxilin (Muscle-derived protein 77) (hMDP77). - *Xenopus tropicalis* | LNO, LNC, NLG | Promotes neurite-outgrowth (Specifically expressed in skeletal and cardiac muscle) | -2.4 |
| 236 | zslaa0_013696 | zslaa0_013696.z1.scf | no hit | LNO | no hit | -2.4 |
| 237 | NLG2655 | NLG2655-2007-09-10.ab1 | similar to CG3304-PA isoform A - *Apis mellifera* | NLG | unknown function | -2.4 |
| 238 | CL3070 Contig1 | LNC240-2007-12-18.ab1; LNO2725-2007-03-06.ab1 | 19.8 kDa small heat shock protein - *Choristoneura fumiferana* (Spruce budworm) | LNO, LNC | Stress response | -2.4 |
| 239 | CL3261 Contig1 | LNC2628-2008-01-15.ab1; LNC1944-2008-01-15.ab1 | Putative uncharacterized protein *- Aedes aegypti* (Yellowfever mosquito) | LNC | unknown function | -2.4 |
| 240 | NLG1791 | NLG1791-2007-08-23.ab1 | NADH dehydrogenase 1 beta subcomplex subunit 2 mitochondrial precursor [*Lepeophtheirus salmonis*] | NLG | mitochondrial electron transport | -2.4 |
| 241 | CL3597 Contig1 | zslaa0_012031.z1.scf; LNC2990-2008-01-16.ab1 | no hit | LNO, LNC | no hit | -2.4 |
| 242 | LNC1817 | LNC1817-2008-01-14.ab1 | no hit | LNC | no hit | -2.4 |
| 243 | zslaa0_005503 | zslaa0_005503.z1.scf | no hit | LNO | no hit | -2.4 |
| 244 | FN4707 | FN4707-2003-03-06.ab1 | Acetyl-CoA C-acetyltransferase - *Rhodospirillum rubrum* (strain ATCC 11170 / NCIB 8255) | ME | metabolism | -2.4 |
| 245 | LNC1850 | LNC1850-2008-01-14.ab1 | no hit | LNC | no hit | -2.4 |
| 246 | CL220 Contig2 | zslaa0_010815.z1.scf; zslaa0_014434.z1.scf; zslaa0_002163.z1.scf; zslaa0_016305.z1.scf; zslaa0_016328.z1.scf; zslaa0_011665.z1.scf; zslaa0_015320.z1.scf; LNO3234-2007-02-15.ab1 | similar to sphingomyelin phosphodiesterase 2 neutral membrane (neutral sphingomyelinase) - *Monodelphis domestica* | LNO | Esterase | -2.4 |
| 247 | NLG1320 | NLG1320-2007-08-22.ab1 | no hit | NLG | no hit | -2.4 |
| 248 | LNO2140 | LNO2140-2007-03-06.ab1 | similar to Zinc/iron regulated transporter-related protein 1 CG9428-PA - *Apis mellifera* | LNO | zink iron transporter | -2.4 |
| 249 | CL643 Contig2 | zslaa1_010142.z1.scf; LNC2537-2008-01-15.ab1 | Putative uncharacterized protein - *Stenotrophomonas maltophilia* R551-3 | LNO, LNC | carbohydrate metabolism, tricarboxylic acid cycle | -2.4 |
| 250 | CL2206 Contig1 | NLG3096-2007-09-26.ab1; NLG563-2007-06-04.ab1; NLG4342-2009-03-05.ab1 | similar to myosin Va - *Tribolium castaneum* | NLG | myosin | -2.4 |
| 251 | zslaa0_002215 | zslaa0_002215.z1.scf | Putative uncharacterized protein - *Caenorhabditis elegans* | LNO | transferase activity | -2.4 |
| 252 | CL815 Contig1, LNO1 | LNO1-2006-12-04.ab1 | Serine/threonine-protein phosphatase PP1-beta catalytic subunit - *Homo sapiens* (Human) | FB, LNO, NLG | essential for cell division, metabolism | -2.3 |
| 253 | LNC1659 | LNC1659-2008-01-14.ab1 | Lysosomal alpha-mannosidase - *Aedes aegypti* (Yellowfever mosquito) | LNC | carbohydrate metabolism | -2.3 |
| 254 | CL1752 Contig1 | zslaa0_011185.z1.scf; LPU881-2007-08-29.ab1; LF113-2007-01-09.ab1 | Transmembrane protein nessy [*Caligus clemensi*] | LF, LPU, LNO | germ cell development | -2.3 |
| 255 | CL821 Contig1 | zslaa0_009158.z1.scf; zslaa0_016690.z1.scf; zslaa0_011274.z1.scf; LNO748-2007-01-25.ab1; 520_FN1689_A12_070.ab1; LNO3558-2007-03-05.ab1 | similar to protein phosphatase 2A regulatory subunit B isoform b - *Tribolium castaneum* | FB, LNO | Among others negative control of cell growth and division | -2.3 |
| 256 | CL2817 Contig1 | zslaa0_005314.z1.scf; zslaa0_007319.z1.scf | similar to CG1333-PB isoform B - *Tribolium castaneum* | LNO | oxidoreductase activity | -2.3 |
| 257 | L1T_1265 | L1T_1265-2007-01-15.ab1 | >gnl|BL_ORD_ID|670936 GI18810 [*Drosophila mojavensis*] | L1T | (lipid) transport | -2.3 |
| 258 | CL3447 Contig1 | LNC378-2007-12-18.ab1; NLG4226-2009-02-24.ab1 | similar to Zinc/iron regulated transporter-related protein 1 CG9428-PA - *Apis mellifera* | LNC, NLG | unknown function | -2.3 |
| 259 | LNO3315 | LNO3315-2007-02-19.ab1 | Syntaxin-8 [*Lepeophtheirus salmonis*] | LNO | cell communication | -2.3 |
| 260 | LN01295 | LN01295-2007-02-08.ab1 | no hit | LNO | no hit | -2.3 |
| 261 | zslaa0_011769 | zslaa0_011769.z1.scf | Sphingomyelin phosphodiesterase 2 [*Lepeophtheirus salmonis*] | LNO | signal transduction | -2.3 |
| 262 | CL3180 Contig1 | NLG1449-2007-08-22.ab1; NLG2452-2007-09-14.ab1 | adiponectin receptor [*Aedes aegypti*] | NLG | glucose and fatty acid metabolism | -2.3 |
| 263 | CL220 Contig1 | zslaa0_008231.z1.scf; LNO1802-2007-02-16.ab1; zslaa0_002324.z1.scf; NLG2613-2007-09-10.ab1 | similar to sphingomyelin phosphodiesterase 2 neutral membrane (neutral sphingomyelinase) *- Monodelphis domestica* | LNO, NLG | signal transduction | -2.3 |
| 264 | LNC1486, LNC1271 | LNC1486-2007-12-21.ab1, LNC1271-2007-12-21.ab1 | Sterol regulatory element binding protein 1 - *Gallus gallus* (Chicken) | LNC | fatty acid metabolism | -2.3 |
| 265 | CL137 Contig1 | zslaa0_001616.z1.scf; zslaa0_011817.z1.scf; zslaa0_003725.z1.scf; zslaa0_004575.z1.scf; zslaa0_005510.z1.scf; zslaa0_008792.z1.scf; zslaa0_005545.z1.scf; zslaa0_004508.z1.scf; zslaa0_016682.z1.scf; HA513-2005-02-14.ab1; LNC3953-2009-03-05.ab1; LNO2340-2007-02-27.ab1; LPU440-2007-09-05.ab1; LNO1774-2007-02-08.ab1; LNC2688-2008-01-15.ab1; LNO1909-2007-02-19.ab1; NLG4366-2009-03-05.ab1; LNO2158-2007-03-06.ab1; LNC2270-2008-01-15.ab1 | ADP/ATP translocase 1 [*Lepeophtheirus salmonis*] | LPU, HA, LNO, LNC, NLG | transporter | -2.3 |
| 266 | LNO2377 | LNO2377-2007-02-27.ab1 | hypothetical protein - *Gallus gallus* | LNO | unknown function | -2.3 |
| 267 | FN6178 | FN6178-2005-04-05.ab1 | Phosphotyrosyl phosphatase activator - *Aedes aegypti* (Yellowfever mosquito) | ME | phosphatase regulation | -2.3 |
| 268 | zslaa0_010222 | zslaa0_010222.z1.scf | Signal peptidase complex subunit 3 [*Lepeophtheirus salmonis*] | LNO | signal peptide processing | -2.3 |
| 269 | CL2246 Contig1 | NLG440-2007-06-04.ab1; LNC1903-2008-01-15.ab1; LNC3489-2009-02-09.ab1 | AMP dependent coa ligase - *Aedes aegypti* (Yellowfever mosquito) | LNC, NLG | metabolic | -2.3 |
| 270 | L1T1670 | L1T1670-2007-01-12.ab1 | no hit | L1T | no hit | -2.3 |
| 271 | CL2973 Contig1 | zslaa0_018490.z1.scf; HA164-2004-07-28.ab1 | similar to Protein C14orf166 - *Tribolium castaneum* | HA, LNO | unknown function | -2.3 |
| 272 | NLG2277 | NLG2277-2007-09-10.ab1 | Sphingomyelin phosphodiesterase 2 [*Lepeophtheirus salmonis*] | NLG | signal transduction | -2.2 |
| 273 | LNO1628 | LNO1628-2007-02-15.ab1 | Nogo-B receptor [*Lepeophtheirus salmonis*] | LNO | embryonal development | -2.2 |
| 274 | NLG4743 | NLG4743-2009-03-05.ab1 | similar to CG7004-PA isoform A - *Tribolium castaneum* | NLG | unknown function | -2.2 |
| 275 | CL1319 Contig1* | LNO902-2007-02-13.ab1; HA336-2004-08-13.ab1; FN4958-2003-03-12.ab1; HA627-2005-02-24.ab1 | Ornithine aminotransferase mitochondrial precursor - *Drosophila melanogaster* (Fruit fly) | ME, HA, LNO | Amino acid biosynthesis | -2.2 |
| 276 | CL1824 Contig1 | zslaa0_003745.z1.scf; NLG3154-2007-09-27.ab1; FN3689-2002-09-18.ab1 | Androgen-induced protein 1 [*Caligus clemensi*] | SB, LNO, NLG | androgen induced, unknown function | -2.2 |
| 277 | LNC2065 | LNC2065-2008-01-15.ab1 | similar to lethal (1) G0334 CG7010-PC isoform C isoform 1 - *Apis mellifera* | LNC | embryonic development | -2.2 |
| 278 | LNC1664* | LNC1664-2008-01-14.ab1 | *Caligus clemensi* clone ccle-evs-509-225 Translation initiation factor eIF-2B subunit alpha/beta/delta-like protein putative mRNA, complete cds | LNC | Translation initiation (among others negative regulation of translational initiation in response to stress) | -1.6 |

# Supplementary table 4. GO annotation enriched in up- or down-regulated clusters respectively. GO annotation

| A) Cellular component | | |
| --- | --- | --- |
| **Go term** |  |  |
| **Enriched in down-regulated clusters/singletons:** | **Enrichment** | **Exact test p-value** |
| integral to membrane | 2.0 | 9.61E-07 |
| membrane part | 1.8 | 3.50E-05 |
| membrane | 1.6 | 1.08E-04 |
| lysosome | 3.7 | 0.002 |
| endomembrane system | 3.2 | 0.003 |
| nuclear lamina | 35.7 | 0.004 |
| apical part of cell | 23.8 | 0.007 |
| vacuole | 2.9 | 0.009 |
| **Enriched in up-regulated clusters/singletons:** | **Enrichment** | **Exact test p-value** |
| ribosome | 3.7 | 2.65E-12 |
| ribonucleoprotein complex | 2.4 | 1.15E-08 |
| cytoplasmic part | 1.6 | 1.05E-07 |
| extracellular region | 2.4 | 3.87E-07 |
| non-membrane-bound organelle | 2.1 | 4.33E-07 |
| chloroplast | 2.8 | 7.35E-07 |
| cytoplasm | 1.4 | 9.14E-07 |
| plastid | 2.8 | 9.86E-07 |
| extracellular matrix part | 12.0 | 1.05E-06 |
| collagen | 13.9 | 2.01E-06 |
| basement membrane | 13.0 | 5.29E-05 |
| ribosomal subunit | 4.0 | 2.95E-04 |
| large ribosomal subunit | 5.8 | 6.00E-04 |
| amyloplast | 8.7 | 9.02E-04 |
| intracellular non-membrane-bound organelle | 1.5 | 9.72E-04 |
| intracellular organelle | 1.2 | 0.004 |
| organelle | 1.2 | 0.004 |
| extracellular matrix | 2.4 | 0.005 |
| organelle inner membrane | 3.5 | 0.007 |
| B) Molecular function | | |
| **Enriched in down-regulated clusters/singletons:** | **Enrichment** | **Exact test p-value** |
| acyltransferase activity | 3.5 | 1.89E-05 |
| transferase activity, transferring groups other than amino-acyl groups | 3.4 | 2.47E-05 |
| transferase activity, transferring acyl groups | 3.3 | 3.01E-05 |
| lipid transporter activity | 7.9 | 2.32E-04 |
| CoA-ligase activity | 10.5 | 2.61E-04 |
| acid-thiol ligase activity | 10.5 | 2.61E-04 |
| transferase activity, transferring acyl groups, acyl groups converted into alkyl on transfer | 13.0 | 5.82E-04 |
| chymosin activity | 26.8 | 6.47E-04 |
| ligase activity, forming carbon-sulfur bonds | 7.4 | 0.001 |
| renin activity | 21.4 | 0.001 |
| cathepsin E activity | 21.4 | 0.001 |
| cathepsin D activity | 21.4 | 0.001 |
| oxidoreductase activity | 1.7 | 0.002 |
| exopeptidase activity | 3.4 | 0.002 |
| pepsin A activity | 15.3 | 0.002 |
| catalytic activity | 1.3 | 0.002 |
| vitamin binding | 3.2 | 0.003 |
| carboxypeptidase activity | 4.6 | 0.003 |
| secondary active transmembrane transporter activity | 2.9 | 0.003 |
| cofactor binding | 2.1 | 0.004 |
| L-amino-acid oxidase activity | 35.7 | 0.004 |
| ecdysteroid hormone receptor activity | 35.7 | 0.004 |
| retinoic acid receptor activity | 35.7 | 0.004 |
| ATP citrate synthase activity | 35.7 | 0.004 |
| glycine N-methyltransferase activity | 35.7 | 0.004 |
| phosphatase activator activity | 35.7 | 0.004 |
| NAD(P)+ transhydrogenase (AB-specific) activity | 35.7 | 0.004 |
| metal ion binding | 1.4 | 0.004 |
| pyridoxal phosphate binding | 4.2 | 0.004 |
| ion binding | 1.4 | 0.005 |
| carboxypeptidase B activity | 10.7 | 0.005 |
| sucrose alpha-glucosidase activity | 23.8 | 0.007 |
| sodium-dependent phosphate transmembrane transporter activity | 23.8 | 0.007 |
| thyroid hormone receptor activity | 23.8 | 0.007 |
| alkaline phosphatase activity | 23.8 | 0.007 |
| NAD(P) transhydrogenase activity | 23.8 | 0.007 |
| beta-fructofuranosidase activity | 23.8 | 0.007 |
| oxidoreductase activity, acting on NADH or NADPH, NAD or NADP as acceptor | 23.8 | 0.007 |
| aminoacylase activity | 23.8 | 0.007 |
| steroid dehydrogenase activity | 8.9 | 0.007 |
| carboxypeptidase A activity | 8.2 | 0.009 |
| **Enriched in up-regulated clusters/singletons:** | **Enrichment** | **Exact test p-value** |
| structural constituent of ribosome | 3.7 | 2.30E-12 |
| structural molecule activity | 2.5 | 3.19E-10 |
| chitin binding | 4.5 | 4.45E-08 |
| polysaccharide binding | 3.9 | 6.90E-08 |
| pattern binding | 3.9 | 8.02E-08 |
| rRNA binding | 4.6 | 2.54E-06 |
| extracellular matrix structural constituent | 8.2 | 3.53E-05 |
| carbohydrate binding | 2.2 | 4.19E-05 |
| isomerase activity | 2.1 | 8.08E-04 |
| pyridoxal phosphate binding | 3.4 | 0.002 |
| betaine-aldehyde dehydrogenase activity | 7.2 | 0.002 |
| intramolecular transferase activity, phosphotransferases | 7.2 | 0.002 |
| aldehyde dehydrogenase (NAD) activity | 7.2 | 0.002 |
| aldehyde dehydrogenase [NAD(P)+] activity | 7.2 | 0.002 |
| intramolecular transferase activity | 5.2 | 0.002 |
| amino acid kinase activity | 8.7 | 0.003 |
| hydrolase activity, acting on carbon-nitrogen (but not peptide) bonds | 2.6 | 0.004 |
| retinal dehydrogenase activity | 7.7 | 0.004 |
| formyltetrahydrofolate dehydrogenase activity | 7.7 | 0.004 |
| glyceraldehyde-3-phosphate dehydrogenase (NADP+) activity | 7.7 | 0.004 |
| catalytic activity | 1.2 | 0.005 |
| magnesium ion binding | 2.0 | 0.005 |
| protease inhibitor activity | 2.6 | 0.006 |
| RNA binding | 1.6 | 0.006 |
| arginine kinase activity | 10.4 | 0.007 |
| guanidinoacetate kinase activity | 10.4 | 0.007 |
| oxidoreductase activity, acting on the CH-NH group of donors, NAD or NADP as acceptor | 4.6 | 0.008 |
| glycine hydroxymethyltransferase activity | 5.8 | 0.009 |
| hydroxymethyl-, formyl- and related transferase activity | 5.8 | 0.009 |
| C) Biological process | | |
| **Enriched in down-regulated clusters/singletons:** | **Enrichment** | **Exact test p-value** |
| cellular lipid metabolic process | 3.3 | 2.07E-06 |
| lipid metabolic process | 2.7 | 7.05E-05 |
| lipid biosynthetic process | 3.4 | 1.98E-04 |
| lipid transport | 6.4 | 2.23E-04 |
| digestion | 3.8 | 2.51E-04 |
| antigen processing and presentation of peptide antigen via MHC class II | 21.4 | 0.001 |
| antigen processing and presentation of exogenous peptide antigen | 21.4 | 0.001 |
| antigen processing and presentation of peptide or polysaccharide antigen via MHC class II | 21.4 | 0.001 |
| antigen processing and presentation of peptide antigen | 21.4 | 0.001 |
| multicellular organismal process | 2.0 | 0.001 |
| regulation of biological quality | 2.3 | 0.001 |
| female pregnancy | 17.8 | 0.001 |
| steroid metabolic process | 5.0 | 0.002 |
| antigen processing and presentation of exogenous antigen | 15.3 | 0.002 |
| fatty acid metabolic process | 3.1 | 0.003 |
| rostrocaudal neural tube patterning | 35.7 | 0.004 |
| midbrain-hindbrain boundary development | 35.7 | 0.004 |
| regulation of defense response to virus | 35.7 | 0.004 |
| regulation of response to biotic stimulus | 35.7 | 0.004 |
| sphingolipid metabolic process | 10.7 | 0.005 |
| regulation of body fluid levels | 3.2 | 0.005 |
| hemostasis | 3.2 | 0.005 |
| blood coagulation | 3.2 | 0.005 |
| membrane lipid metabolic process | 4.0 | 0.005 |
| antigen processing and presentation | 9.7 | 0.006 |
| wound healing | 3.1 | 0.006 |
| response to external stimulus | 2.6 | 0.007 |
| regulation of defense response | 23.8 | 0.007 |
| response to wounding | 2.7 | 0.007 |
| fatty acid biosynthetic process | 3.6 | 0.008 |
| cholesterol metabolic process | 8.2 | 0.009 |
| organic acid biosynthetic process | 3.6 | 0.009 |
| carboxylic acid biosynthetic process | 3.6 | 0.009 |
| **Enriched in up-regulated clusters/singletons:** | **Enrichment** | **Exact test p-value** |
| cellular biosynthetic process | 2.2 | 4.86E-12 |
| translation | 2.6 | 2.21E-10 |
| cellular carbohydrate metabolic process | 3.0 | 3.33E-10 |
| cellular polysaccharide metabolic process | 4.0 | 1.04E-09 |
| carbohydrate metabolic process | 2.4 | 2.01E-09 |
| polysaccharide metabolic process | 3.6 | 2.46E-09 |
| chitin metabolic process | 4.4 | 3.23E-08 |
| N-acetylglucosamine metabolic process | 4.3 | 5.41E-08 |
| glucosamine metabolic process | 4.3 | 5.41E-08 |
| amino sugar metabolic process | 4.2 | 6.39E-08 |
| biosynthetic process | 1.6 | 8.31E-08 |
| macromolecule biosynthetic process | 1.8 | 1.04E-06 |
| metabolic process | 1.3 | 1.16E-06 |
| cellular metabolic process | 1.3 | 2.90E-06 |
| primary metabolic process | 1.3 | 4.94E-06 |
| anion transport | 4.2 | 1.42E-05 |
| inorganic anion transport | 4.7 | 1.93E-05 |
| cellular macromolecule metabolic process | 1.4 | 3.34E-05 |
| phosphate transport | 4.8 | 7.47E-05 |
| macromolecule metabolic process | 1.3 | 1.07E-04 |
| gene expression | 1.5 | 1.61E-04 |
| heterocycle metabolic process | 3.3 | 2.69E-04 |
| nucleotide catabolic process | 9.6 | 6.29E-04 |
| biogenic amine biosynthetic process | 6.1 | 0.001 |
| glycine betaine biosynthetic process from choline | 7.9 | 0.001 |
| choline metabolic process | 7.9 | 0.001 |
| glycine betaine biosynthetic process | 7.9 | 0.001 |
| glycine betaine metabolic process | 7.9 | 0.001 |
| biogenic amine metabolic process | 4.2 | 0.001 |
| amino acid derivative biosynthetic process | 5.8 | 0.001 |
| ethanolamine and derivative metabolic process | 7.2 | 0.002 |
| betaine biosynthetic process | 7.2 | 0.002 |
| betaine metabolic process | 7.2 | 0.002 |
| folic acid and derivative metabolic process | 5.5 | 0.002 |
| ion transport | 1.8 | 0.002 |
| G-protein coupled receptor protein signaling pathway | 2.4 | 0.003 |
| nucleobase, nucleoside and nucleotide metabolic process | 2.0 | 0.003 |
| group transfer coenzyme metabolic process | 5.0 | 0.003 |
| amino acid derivative metabolic process | 3.3 | 0.003 |
| cellular polysaccharide biosynthetic process | 3.6 | 0.004 |
| nucleoside metabolic process | 2.8 | 0.004 |
| sulfur compound biosynthetic process | 3.9 | 0.004 |
| 10-formyltetrahydrofolate catabolic process | 7.7 | 0.004 |
| folic acid and derivative catabolic process | 7.7 | 0.004 |
| 10-formyltetrahydrofolate metabolic process | 7.7 | 0.004 |
| polysaccharide biosynthetic process | 3.8 | 0.005 |
| biological adhesion | 2.0 | 0.005 |
| cell adhesion | 2.0 | 0.005 |
| nitrogen compound metabolic process | 1.7 | 0.007 |
| glucan metabolic process | 4.8 | 0.007 |
| glycogen metabolic process | 4.8 | 0.007 |

# Supplementary table 5. Genes involved in fatty acid/ lipid metabolism and transport

| **Cluster/ singleton** | **Hit UniRef/ GenBank** | **Known function found in other organism** | **Originating from library** | **Times regulated** |
| --- | --- | --- | --- | --- |
| **Up-regulated** | | | | |
| CL279 Contig2 | Prosaposin | involved in glycolipid transport and acts probably by isolating the lipid substrate from the membrane surroundings, thus making it more accessible to the soluble degradative enzymes “ “ | preadult | +2.4 |
| CL279 Contig1 | similar to prosaposin partial | “ “ | copepodit, adult female without T1, L1T,LNO | +2.3 |
| **Down-regulated** | | | | |
| zslaa0_008831 | Delta5 fatty acid desaturase A | Specific for desaturation of the 5 position in C16 and C18 fatty acids, Fatty acid biosynthesis | LNO | -1.4272 E+121 |
| CL558 Contig1/2 | Elongation of very long chain fatty acids protein AAEL008004 | Fatty acid biosynthesis, integral to membrane | LNO | -1.33324 E+13 |
| CL2325 Contig1 | Succinyl-CoA:3-ketoacid-coenzyme A transferase 1 mitochondrial precursor | Key enzyme for ketone body catabolism; transfers the CoA moiety from succinate to acetoacetate; mitochondrial matrix | LNO, copepodit | -29.8 |
| LNO2518 | Long-chain-fatty-acid-CoA ligase ACSBG1 | Mediates activation of long-chain fatty acids for both synthesis of cellular lipids, and degradation via beta-oxidation.  mouse: In brain, it is present in cerebral cortical and cerebellar neurons and in steroidogenic cells of the adrenal gland, testis and ovary (at protein level). Fatty acid metabolism | LNO | -5.2 |
| PU822 | scavenger receptor class B, member 2/1 | cell adhesion, surface protein capable of binding and internalization of modified lipoproteins, integral to membrane.  (SCARB1: receptor for HDL, Facilitates the flux of free and esterified cholesterol between the cell surface and extracellular donors and acceptors, turtle: seems to be up regulated during egg development; SCARB2: May act as a lysosomal receptor, belongs to the CD36 family) | preadult | -5.2 |
| CL1748 Contig1 | 15-hydroxyprostaglandin dehydrogenase | mouse: Prostaglandin inactivation. Contributes to the regulation of events that are under the control of prostaglandin levels. Catalyzes the NAD-dependent dehydrogenation of lipoxin A4 to form 15-oxo-lipoxin A4 Fatty acid metabolism | LNO | -4.3 |
| CL3426 Contig1 | Acetoacetyl-CoA synthetase | Activates acetoacetate to acetoacetyl-CoA. May be involved in utilizing ketone body for the fatty acid-synthesis during adipose tissue development, rat: cellular response to cholesterol, cellular response to glucose stimulus | Egg, LNO | -4.1 |
| CL1136 Contig1 | Lysophosphatidylcholine acyltransferase 2-B | Phospholipid biosynthesis, integral to membrane | LNO, copepodit | -4.0 |
| FN1168 | cytochrome P450 2J2 | human: metabolizes arachidonic acid predominantly via a NADPH-dependent olefin epoxidation to all four regioisomeric cis-epoxyeicosatrienoic acids, epoxygenase P450 pathway, linoleic acid metabolic process | Adult female without T1 | -3.9 |
| LNO666 | Synaptic glycoprotein SC2 | lipid metabolic process, steroidsynthesis, integral to membrane | LNO | -3.3 |
| CL75 Contig1 | Cellular retinoic acid/retinol binding protein | lipid transport | LNO, egg, preadult, young adult female | -2.6 |
| L1T_1265 | Cytoplasmic phosphatidylinositol transfer protein 1 | mouse: Phosphatidylinositol transfer proteins mediate the monomeric transport of lipids by shielding a lipid from the aqueous environment and binding the lipid in a hydrophobic cavity; may play a role in the phosphoinositide-mediated signaling in the neural development; lipid transport | L1T | -2.3 |
| LNC1486 | Sterol regulatory element binding protein 1 | Cholesterol metabolism: Transcriptional activator required for lipid homeostasis. Regulates transcription of the LDL receptor gene as well as the fatty acid and to a lesser degree the cholesterol synthesis pathway. Binds to the sterol regulatory element 1 | copepodit | -2.3 |
| CL3180 Contig1 | adiponectin receptor | Receptor for globular and full-length adiponectin, an essential hormone that modulates a number of metabolic processes, including glucose regulation and lipid metabolism. Mediates increased AMPK and PPAR-α ligand activities, as well as fatty acid oxidation and glucose uptake by adiponectin. It also regulates cholesterol synthesis via phosphorylation and inactivation of hormone-sensitive lipase and hydroxymethylglutaryl-CoA reductase; integral to membrane | egg | -2.3 |

# Supplementary table 6. Genes involved in development

| **cluster/ singleton** | **Uniref hit** | **Function found in other organism** | **found in library** | **Mean Fold Change** |
| --- | --- | --- | --- | --- |
| **Up-regulated** | | | | |
| CL3400Contig1 | neuroparsin | a multifunctional neuro hormone which is among others is known to inhibite the effects of juvenile hormone in locusta and to induce an increase in hemolymph lipid and trehalose levels; interacts with the 20E to enhance neurite growth.  In locusts neuroparsin inhibits vitellogenesis | Adult male | +1348 |
| CL937Contig1 | similar to Collagen alpha-1(XI) chain precursor isoform 1 | Among others embryonic skeletal system morphogenesis and other morphogenesis | preadult, L1T, LNO | +27.0 |
| LNO3402 | spermine synthase | highly specific aminopropyltransferase; Gyro mice, which have an X-chromosomal deletion including the spermine synthase (SMS) gene, lack all spermine and have a greatly reduced size, sterility, deafness, neurological abnormalities, and a tendency to sudden death. Mutations in the human SMS lead to a rise in spermidine and reduction of spermine causing Snyder-Robinson syndrome, characterized by mental retardation, skeletal defects, hypotonia, and movement disorders. | LNO | +16.4 |
| zslaa0_013180 | Spermine synthase | ”-” | LNO | +15.2 |
| CL456Contig1 | similar to CG4300-PB isoform B | spermidine synthase; spermine biosynthetic process (spermine:a polybasic amine found in human sperm, in ribosomes and in some viruses and involved in nucleic acid packaging) | preadult, LNO | +15.0 |
| zslaa0_018437 | Integrin beta-PS precursor | Among others: central nervous system development | LNO | +11.6 |
| LNO1878 | similar to Dermis expressed 1 -=Twist2 | Involved in postnatal glycogen storage and energy metabolism; Among others: Inhibits the premature or ectopic differentiation of preosteoblast cells during osteogenesis  (Mainly nuclear during embryonic development. Cytoplasmic in adult tissues)  Counteracting the proapoptotic effects of myc oncogene  during osteoblast development, | LNO | +9.65 |
| LNC203 | Hypoxanthine phosphoribosyltransferase 1 | among others: cerebral cortex neuron differentiation | copepodit | +5.8 |
| CL2951Contig | Laminin A chain | mediate the attachment, migration and organization of cells into tissues during embryonic development by interacting with other extracellular matrix components | L1T, LNO | +4.9 |
| CL42Contig3 | similar to CG33950-PD isoform D | Trol-mediated FGF signaling (required for initiation of neuroblast proliferation) | egg, preadult, LNO | +4.8 |
| zslaa0_009859 | Spondin, Contains 1 BPTI/Kunitz inhibitor domain (bad hit) | multicellular organismal development | LNO | +4.2 |
| CL302Contig1 | similar to LEThal family member (let-2) | tumor suppressor protein lethal (2) giant larvae; epithelial cell polarity during development; is required to prevent ectopic activation of the signal protein Notch in developmental processes in Drosophila. | copepodit, preadult, L1T, LNO | +2.9 |
| LF171 | similar to CG33653-PA isoform A | Strongly expressed in neural-specific cells at the onset of early stages of neuronal differentiation;  [positive regulation of calcium ion-dependent exocytosis](http://www.ebi.ac.uk/ego/DisplayGoTerm?id=GO:0045956).  Disruption phenotype drosophila: locomotory deficits and complete embryonic lethality. | preadult | 2.92 |
| CL3163Contig1 | Short gastrulation | zygotic determination of anterior/posterior axis | egg, copepodit | +2.9 |
| zslaa0_009245 | GATA-4 transcription factor | represses yolk protein precursor genes in the mosquito Aedes aegypti via interaction with the CtBP corepressor  Controlling Adipogenesis  Embryonic morphogenesis and differentiation | LNO | +2.9 |
| CL2344Contig1 | similar to sprouty-related EVH1 domain containing 2 | important for multicellular organismal development by inhibiting growth-factor-mediated activation of MAP kinase | adult female without T1 | +2.9 |
| CL2253Contig1 | similar to Tramtrack protein beta isoform (Tramtrack p69) (Fushi tarazu repressor protein) | neuron development;  Fushi tarazu repressor protein, promoting glial differentiation and suppressing neuronal development, involved in nervous system development in drosophila. | copepodit, LNO | +2.8 |
| LF747 | Cofilin/actin-depolymerizing factor homolog | drosophila: many of which group under: anatomical structure development; establishment of planar polarity; cell division; cellular process involved in reproduction; leg morphogenesis; regulation of cellular component organization; protein complex biogenesis; central nervous system development; female gonad development; actin filament-based process | preadult | +2.7 |
| NLG1386 | Innexin inx2 | morphogenesis of embryonic epithelium | egg | +2.6 |
| zslaa0_008243 | Broad-complex core protein isoform 6 | Among others: ecdysone-mediated induction of salivary gland cell autophagic cell death | LNO | +2.6 |
| NLG1079 | PIWI | May play a role in the development of neurons and oligodendrogalia in the CNS  Mediates a somatic signaling mechanism required for the maintenance of germline stem cells to produce and maintain a daughter germline stem cell | egg | +2.5 |
| CL2668Contig1 | Cytochrome P450 CYP315A1 | central nervous system development, pathway steroid biosynthesis, ecdysteroid biosynthesis | LNO | +2.5 |
| LPU407 | Cofilin/actin-depolymerizing factor homolog | See LF747 | preadult | +2.4 |
| **Down-regulated** | | | | |
| a) FN486  =b) | embryo cathepsin L-associated protein | Egg yolk | adult female without T1, | -6.3694 E+30 |
| b) CL1Contig20 | embryo cathepsin L-associated protein | Egg yolk | adult female without T1, LNO | -1.55022 E+24 |
| zslaa0_009321 | Transmembrane protein nessy | controlled by Hox proteins during Drosophila embryogenesis; germ cell migration; sperm individualization | LNO | -1094784676 |
| FN4981 | Transmembrane protein nessy | Germ cell migration | adult female without T1, | -317240122 |
| CL1124Contig1 | similar to steroid dehydrogenase | involved in steroidogenesis | LNO | -120000000 |
| zslaa0_008224 | Similar to to vitellogenic carboxypeptidase, a serine carboxypeptidase expressed in mosquito ovaries | May play a role in activating hydrolytic enzymes that are involved in the degradation of yolk proteins in developing embryos or may function as an exopeptidase in the degradation of vitellogenin | LNO | -3768346 |
| CL3565Contig1 | Longitudinals lacking protein-like | required for axon growth and guidance in the central and peripheral nervous systems; apoptosis | LNO | -9.5 |
| CL19Contig2 | Phospholipid-hydroperoxide glutathione peroxidase | Protects cells against membrane lipid peroxidation and cell death; Essential for embryonic development | preadult, adult female without T1, L1T,LNO | -9.2 |
| CL449Contig1 | granulin-like | may play a role in inflammation, wound repair, and tissue remodeling  cell growth (different members of the granulin protein family may act as inhibitors, stimulators, or have dual actions on cell growth). | L1T,LNO | -7.0 |
| LF396, LNO3760 | Juvenile hormone-inducible protein putative |  | preadult, | -5.4 |
| CL1127Contig1 | 7-dehydrocholesterol reductase | cholesterol production | LNO | -5.2 |
| CL1062Contig1  (FN462, FN4787, CL1933Contig1, CL1582Contig1, CL3Contig1, CL1062Contig1, FN825, LNO3333) | vitellogenin 2 | Egg yolk | adult female without T1, LNO | -4.9 |
| CL2946Contig1 | RXR-like protein |  | chalimus,adult female without T1, | -4.4 |
| CL762Contig1 | Ultraspiracle nuclear receptor |  | egg,LNO | -4.2 |
| CL2105Contig1 | Moesin/ezrin/radixin | Regulator of the Hippo/SWH (Sav/Wts/Hpo) signaling pathway-plays a pivotal role in organ size control and tumor suppression by restricting proliferation and promoting apoptosis | copepodit, adult female without T1, L1T, | -3.8 |
| LNC1515 | similar to dumpy CG33196-PB | Required to orient neuroblasts QL and QR correctly on the anterior/posterior axis | copepodit, | -3.5 |
| CL2649Contig1 | Muscle lim protein | Positive regulator of myogenesis | copepodit,LNO | -2.9 |
| CL335Contig1  (CL2931Contig1, CL335Contig1) | Troponin I | Involved in the development and maintenance of muscle and nervous system | LNO | -2.7 |
| zslaa0_010489 | GA13884-PA - | Cell communication  EGF-like domain | LNO | -2.5 |
| CL1752Contig1 | Transmembrane protein nessy | See zslaa0_009321 | preadult,LNO | -2.3 |
| LNC2065 | similar to lethal (1) G0334 CG7010-PC isoform C isoform 1 | Sex specific differentiation | copepodit, | -2.2 |
| LNO1628 | Nogo-B receptor | Acts as a specific receptor for the N-terminus of Nogo-B, a neural and cardiovascular regulator; regulate vascular remodeling and angiogenesis | LNO | -2.2 |

# Supplementary table 7. Genes involved in apoptosis

| **cluster/ singleton** | **hit UniRef** | **Function found in other organism** | **found in library** | **Mean Fold Change** |
| --- | --- | --- | --- | --- |
| **Up-regulated** | | | | |
| LF1306 | Coagulation factor VII | negative regulation of apoptosis | preadult | +3.9 |
| CL3404Contig1 | similar to 24 dehydrocholesterol reductase | among others: anti-apoptosis, male genitalia development, skin development, sterol biosynthesis | egg, copepodit | +3.7 |
| CL1562Contig1 | similar to Glutamate-cysteine ligase catalytic subunit (GCS heavy chain) isoform 1 | Among others anti-apoptosis | copepodit, LNO | +2,6 |
| NLG2553 | similar to ADAM metallopeptidase with thrombospondin type 1 motif 9 preproprotein | Among others: apoposis inhibitor | egg | +3.0 |
| CL246Contig1 | Sequestosome-1 | May be involved in cell differentiation, apoptosis, immune response and regulation of K+ channels. | egg, copepodit, adult female without T1, adult male, LNO | +2.7 |
| HA575 | Mekk1 CG7717-PB, isoform B | MEKK1 induces apoptosis by dysregulation of MAP kinase pathways but role of MEKK1 in apoptosis contradictory | adult male | +2.5 |
| LNO1018 | Cell death regulatory protein GRIM19* | drosophila: salivary gland cell autophagic cell death; mouse: involved in the interferon/all-trans-retinoic acid (IFN/RA) cell death pathway; functional component of mitochondrial complex I; essential for early embryonic development  (overexpression of GRIM-19 enhances the sensitivity of cells to IFN-RA-induced death) but also interacts with antiapoptotic factor GW112; pleiotropic roles within a cell. | LNO | +2.3 |
| CL161Contig1 | Elongation factor 1-gamma | protein biosynthesis, Drosophila: salivary gland cell autophagic cell death | copepodit, preadult, adult female without T1, LNO | +2.2 |
| **Down-regulated** | | | | |
| zslaa0_008255 | apoptosis-inducing factor 3-like | Induces apoptosis through a caspase dependent pathway | LNO | -4.3 |
| FN-1039 | Cathepsin L cysteine protease ICP1 | overall degradation of proteins in lysosomes; besides essential for female and male fertility in drosophila | adult female without T1 | -3.9 |
| CL2105Contig1 | Moesin/ezrin/radixin | regulator of the Hippo/SWH signaling pathway, a pathway restricting proliferation and promoting apoptosis | copepodit, adult female without T1, L1T | -3.8 |
| CL36Contig4 | Matrix metalloproteinase 9 | collagen degradation | preadult,L1T,LNO | -3.3 |
| CL2697Contig1 | L-amino-acid oxidase - | ability to induce apoptosis | LNO | -3.2 |
| LNO3643 | Endoplasmic reticulum lumenal L-amino acid oxidase precursor | Lysosome | LNO | -3.1 |
| LF395 | inhibitor of apoptosis-2 (dIAP2) | inhibitor of apoptosis | preadult | -2.6 |
| CL220Contig2 | sphingomyelin phosphodiesterase 2 neutral, membrane | Among others induction of apoptosis; ) Neutral sphingomyelinase activity was first described in fibroblasts from patients with Niemann-Pick disease | LNO | -2.4 |

# Supplementary table 8. CYP and ABC-transporter

| **Cluster/singleton** | **CYP no** | **regulated in LsRXR RNAi lice** | **Libraries** |
| --- | --- | --- | --- |
| **Up-regulated** | | | |
| CL2668Contig1 | CYP315A1 = shadow | +2.47 | LNO |
| NLG187 | P450 9f2 | +3.20 | egg |
| CL3275Contig1 | 3A72 | +3.79 | LNO |
| LNC582 | 330A1 | +4.80 | LNC |
| CL1925Contig1 | P450 3A31 | +4.43 | copepodit |
| CL3785Contig1 | P450 3A24 | +4.37 | preadult,LNO |
| **Down-regulated** | | | |
| CL2590Contig1 | 304a1 | -3.96 | LNO |
| FN1168 | P450 2J2 | -3.87 | adult female without T1 |
| **Not regulated** | | | |
| CL2588Contig1 | P450 302a1 =disembodied | Not regulated | LNO |
| CL3108Contig1 | CYP P4502J and hit with daphnia spook | Not regulated | LNO, NLG |
| **Cluster/singleton** | **ABC-transporter** | **regulated in LsRXR RNAi lice** | **Libraries** |
| **Up-regulated** | | | |
| NLG976 | ATP-binding cassette sub-family B (MDR/TAP) member 1A; reacting on xenobiotics and steroids) together with CYP3A | +3.14 | egg |
| **Down-regulated** | | | |
| CL3701Contig1 | ATP-binding cassette sub-family D member 4 | -45.34 | copepodit,LNO |
| **Not regulated** | | | |
| CL1334Contig1 | ATP-binding cassette sub-family B member 8 mitochondrial precursor | Not regulated | LNO, NLG |
| LPU1347 | ATP-binding cassette sub-family A (ABC1) member 1 | Not regulated | preadult |

# Supplementary table 9. Genes regulated in our experiment which have hit with genes from selected tables from Arbeitman 2002

| **table no*** | **flybase no** | **Gene Dmel** | **gene name** | **biological process** | **molecular function** | **cluster/ sinleton** | **times regu-lated** |
| --- | --- | --- | --- | --- | --- | --- | --- |
| **up-regulated** | | | | | | | |
| **Tab 24, female germline** | FBpp0079951 | Elf-PA | Ef1α-like factor | translational termination | translation termination factor activity; GTP binding; translation release factor activity | CL33 Contig1 | 2.4 |
|  | FBgn0003279 | RpL4 | Ribosomal protein L4 | Translation | structural constituent of ribosome | CL174 Contig1 | 2.2 |
|  | FBgn0261606 | RpL27A | Ribosomal protein L27A | mitotic spindle elongation; mitotic spindle organization | structural constituent of ribosome | CL186 Contig1 | 2.3 |
|  | FBgn0000556 | Ef1α48D | Elongation factor 1α48D | determination of adult lifespan | translation elongation factor activity | CL193 Contig1 | 2.2 |
|  | FBgn0005593 | RpL7 | Ribosomal protein L7 | mitotic spindle elongation; mitotic spindle organization | structural constituent of ribosome | CL62 Contig1 | 2.4 |
|  | FBgn0261602 | RpL8 | Ribosomal protein L8 | mitotic spindle elongation; mitotic spindle organization | structural constituent of ribosome | LF1065 | 2.3 |
| **Tab 16, maternal rapidly degraded** | FBpp0070894 | Ubi-p5E-PA | Ubiquitin-5E | ubiquitin-dependent protein catabolic process; protein modification process | protein binding | CL707 Contig1 | 2.3 |
| **Tab 15, maternal rapidly reinduced** | FBpp0290219 | CG32772-PC | - | unknown. | nucleic acid binding | CL1631 Contig1 | 4.2 |
|  | FBpp0072767 | CG8993-PA | - | response to DNA damage stimulus | disulfide oxidoreductase activity | CL1874 Contig1 | 2.3 |
| **Tab 14, maternal core germline** | FBpp0077516 | Uch-PA | biquitin carboxy-terminal hydrolase | protein deubiquitination | ubiquitin thiolesterase activity | CL1896 Contig1 | 2.5 |
|  | FBpp0075508 | 26-29-p-PA | 26-29kD-proteinase | Proteolysis | cysteine-type endopeptidase activity | CL2613 Contig1 | 3.0 |
|  | FBpp0075508 | 26-29-p-PA | 26-29kD-proteinase | Proteolysis | cysteine-type endopeptidase activity | LNO612 | 2.8 |
| **Tab 13, maternal gradual decline** | FBpp0290219 | CG32772-PC | - | Unknown | nucleic acid binding | CL1631 Contig1 | 4.2 |
|  | FBpp0291675 | wnd-PD | wallenda | protein amino acid phosphorylation | protein serine/threonine kinase activity | LNO2936 | 3.4 |
| **Tab 12, strict maternal** | FBpp0082932 | Pxt-PB | Peroxinectin-like | ovarian follicle cell development; prostaglandin biosynthetic process | peroxidase activity | L1T1786 | 3.5 |
|  | FBpp0291408 | Mdr49-PB | Multi drug resistance 49 | response to hypoxia; germ cell migration | ATPase activity, coupled; drug transmembrane transporter activity | NLG976 | 3.1 |
|  | FBpp0110460 | CG17493-PA | - | Unknown | calcium ion binding | zslaa0_01418 | 2.8 |
| **Tab 7, changes during embryogenesis** | FBpp0079951 | Elf-PA | Ef1α-like factor | translational termination | translation termination factor activity; GTP binding; translation release factor activity | CL33 Contig1 | 2.4 |
|  | FBpp0070894 | Ubi-p5E-PA | Ubiquitin-5E | ubiquitin-dependent protein catabolic process; protein modification process | protein binding | CL707 Contig1 | 2.3 |
| **down-regulated** | | | | | | | |
| **Tab 15, maternal rapidly reinduced** | FBgn0000579 | Eno | Enolase | Glycolysis | phosphopyruvate hydratase activity | *FN462* | -4.1 |
|  | FBpp0099765 | Got2-PC | Glutamate oxaloacetate transaminase 2 | synapse assembly; glutamate biosynthetic process; neurotransmitter receptor metabolic process | L-aspartate:2-oxoglutarate aminotransferase activity | CL412 Contig1 | -3.6 |
|  | FBpp0079472 | yip2-PA | yippee interacting protein 2 | fatty acid beta-oxidation | acetyl-CoA C-acyltransferase activity | FN4707 | -2.4 |
|  | FBpp0072135 | Thiolase-PA | Thiolase | fatty acid beta-oxidation | long-chain-3-hydroxyacyl-CoA dehydrogenase activity; acetyl-CoA C-acyltransferase activity | LNO1410 | -2.5 |
| **Tab 14, maternal core germline** | FBpp0087672 | Cyp4p2-PA | Cyp4p2 | oxidation reduction | electron carrier activity | FN1168 | -3.9 |
|  | FBpp0288713 | granny-smith-PE | granny smith | Proteolysis | aminopeptidase activity | CL1876 Contig1 | -3.6 |
|  | FBpp0288713 | granny-smith-PE | granny smith | Proteolysis | aminopeptidase activity | LNC1724 | -3.1 |
|  | FBgn0037298 | CG2604 | - | metabolic process | binding; catalytic activity | CL309 Contig1 | -3.2 |
| **Tab 13, maternal gradual decline** | FBpp0074410 | CG7101-PA | - | Unknown | zinc ion binding; nucleic acid binding | LNO3037 | -108.6 |
| **Tab 12, strict maternal** | FBgn0033236 | CG14764 | - | Unknown | unknown | CL1423 Contig1 | -3.9 |
|  | FBgn0035641 | CG5568 | - | metabolic process | 4-coumarate-CoA ligase activity | CL2246 Contig1 | -2.3 |
| **Tab 7, changes during embryogenesis** | FBpp0072568 | Myo61F-PA | Myosin 61F | determination of left/right symmetry; defense response to bacterium; microvillus organization; mesoderm development | ATPase activity, coupled | zslaa0_01091 | -3.3 |

* adopted from Arbeitman 2002

# 
